# Innervated human cardiac muscle model reveals sympathetic drivers of *KCNH2*-associated arrhythmias

**DOI:** 10.1038/s41467-026-76956-9

**Published:** 2026-09-02

**Authors:** Lennart Valentin Schneider, Michael Gani Setya, Guobin Bao, Daniel Härtter, Aditi Methi, Dennis Krüger, Marie Kristin Schreiber, Ole Jensen, Elisavet Kanari, Kea Aline Schmoll, Narasimha Swamy Telugu, Sadman Sakib, Eun Seo Kim, Aminath Luveysa Fahud, Maham Novin, Aylin Seedorf, Sebastian Diecke, André Fischer, Norman Y. Liaw, Wolfram-Hubertus Zimmermann, Maria-Patapia Zafeiriou

**Affiliations:** 1 Institute of Pharmacology and Toxicology, University Medical Center Göttingen, Göttingen, Germany; 2 DZHK (German Center for Cardiovascular Research), partner site Lower Saxony, Göttingen, Germany; 3 MBExC (Multi-Scale Bioimaging Excellence Cluster), Göttingen, Germany; 4 German Center for Neurodegenerative Diseases, Göttingen, Germany; 5 Department of Clinical Pharmacology, University Medical Center Göttingen, Göttingen, Germany; 6 Max Delbrück Center for Molecular Medicine in the Helmholtz Association (MDC), Technology Platform Pluripotent Stem Cells, Berlin, Germany; 7 Department of Cardiology and Pneumology, University Medical Center, Göttingen, Germany; 8 Fraunhofer Institute for Translational Medicine and Pharmacology (ITMP), Göttingen, Germany; 9 German Center for Child and Adolescent Health (DZKJ), partner site Lower Saxony, Göttingen, Germany; 10 Campus-Institute Data Science (CIDAS), Göttingen, Germany; 11 Else Kröner Fresenius Center for Optogenetic Therapies (EKFZ L2T), Göttingen, Germany; 12 Clinic for Pediatric and Adolescent Medicine, University Medical Center Göttingen, Göttingen, Germany; 13 Collaborative Research Center 1690, University of Göttingen, Göttingen, Germany

**Keywords:** Tissue engineering, Stem-cell biotechnology, Disease model

## Abstract

Cardiac autonomic neurons regulate contractility. Autonomic nervous system dysregulation can cause sympathetic overdrive, leading to heart failure, and fatal arrhythmias. Here, we introduce innervated engineered human myocardium (iEHM), a model of neuro-cardiac junctions, constructed by fusing sympathetic neuronal organoids (SNO) and engineered human myocardium (EHM). Projections of sympathetic neurons formed presynaptic terminals in close proximity to cardiomyocytes and to the extensive vascular network co-developing in iEHM. Contractile responses to optogenetic stimulation of the accordingly engineered neuronal component demonstrated functional neuro-cardiac junctions. Modeling long-QT 2 in iEHM revealed sympathetic neuron hyperactivity and after depolarizations, underscoring a central role for sympathetic drive in *KCNH2*-associated arrhythmias. β-adrenoreceptor blockade was insufficient to rescue the pro-arrhythmic phenotype, while mexiletine targeting both neurons and cardiomyocytes, proved to be more effective. Collectively, our data establishes iEHM as a New Approach Methodology that provides a human-relevant, physiologically integrated model for mechanistic investigations and pharmacological testing.

## Abstract (web summary)

Cardiac autonomic neurons regulate cardiac contractility, and their dysregulation contributes to heart failure and a higher risk of fatal arrhythmias. The study introduces innervated engineered human myocardium (iEHM), as a 3D model to investigate neuro-cardiac communication at structural, molecular, and functional levels.

## Introduction

Sympathetic neurons (SNs) arise from trunk neural crest^1^ under the influence of morphogens such as retinoic acid, bone morphogenetic protein 4 (BMP4), and canonical Wnt activation^1–3^. SNs release noradrenaline (NA), which binds to adrenoreceptors present in cardiac cells (pacemaker or cardiomyocytes (CMs) to modulate contractile performance^4^. In contrast, parasympathetic neurons arising from the cardiac neural crest release acetylcholine (ACh), which negatively regulates pacemaker cell function in the heart and thus reduces cardiac beating rate^4^.

Imbalance of autonomic neuronal regulation is a hallmark of brain and heart diseases (e.g., sudden cardiac death under epilepsy^5^, catecholaminergic polymorphic ventricular tachycardia^6^) as well as heart failure. Sudden unexpected death in epilepsy (SUDEP) is the most frequent cause of death in young adults with uncontrolled epileptic seizures^7^. Genetic analysis in SUDEP patients identified mutations in ion channels, such as hERG encoded by the *KCNH2* gene, as predisposing factors for epilepsy and cardiac long QT syndrome (LQTS)^8–10^. In the heart, *KCNH2* controls delayed inward rectifier potassium channel (*I*_Kr_) activity^11^. *KCNH2* loss-of-function mutations lead to repolarization defects with a prolongation of the cardiac action potential (AP), described as long QT syndrome type 2 (LQT2)^12^. In the brain, *I*_Kr_ loss of function leads to neuronal hyperexcitability, resulting in a high incidence of epileptic seizures^13^. Whether the autonomic nervous system is affected and contributes to cardiac arrhythmias and sudden cardiac death is still poorly understood.

Human tissue engineering in combination with induced pluripotent stem cells (iPSCs) ushered in a new era in human disease modeling. A number of tissue-engineered and organoid models of brain^14–16^ or heart^17–19^ have been developed and employed to model human diseases^16,17,20^. A design principle in heart models is the co-culture of CMs with non-myocytes, such as fibroblasts and endothelial cells^17,21^. Similarly, in brain organoids, neurons co-develop with glial cells^14–16^. Co-culture experiments of CMs and neurons have been performed with a demonstration of cell-cell interactions^2,3,22–24^. Recently, such models have been advanced to the human 3D tissue level^25–27^, allowing neurocardiac interactions at the syncytium level. 3D models with higher complexity and multicellularity may be able to recapitulate neurocardiac co-development. For example, in the developing embryo, sympathetic axons are chemoattracted towards the heart by nerve growth factor (NGF) released from perivascular cells and thus are located close to vessels^28^. NGF is a key player in neuronal homeostasis in the heart^29^, and its dysregulation after myocardial infarction is believed to underlie neuronal remodeling and sympathetic overdrive^30^.

Aiming to generate a 3D neuro-cardiac interface that would allow the study of brain-and-heart-associated pathologies, we patterned our previously established bioengineered neuronal organoid (BENO)^14^ model to a primarily SN identity. We then fused the resulting sympathetic neuronal organoid (SNO) with engineered human myocardium (EHM)^17^ to allow SNs to innervate and functionally interact with the cardiac cells, a tissue which we termed innervated EHM (iEHM). Finally, we functionally characterized the cardiac and neuronal phenotype of two *KCNH2* variants in human iPSC-derived EHM, SNO, and iEHM, as well as the contribution of neuronal dysregulation to arrhythmia predisposition.

## Results

### Generation of a sympathetic neuronal organoid (SNO)

**Fig. 1:**
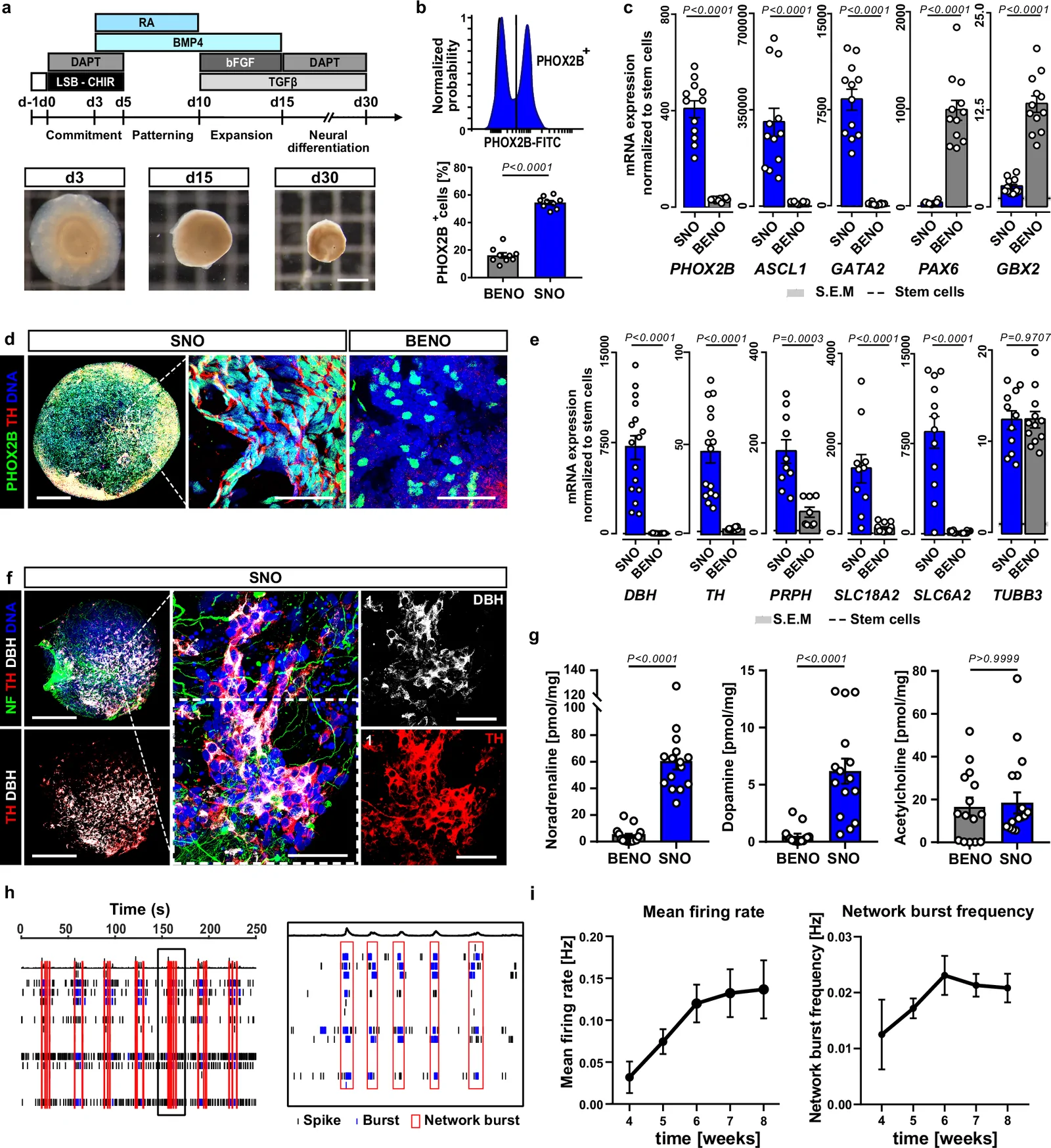
Generation and characterization of SNO. **a** (Top) Schematic of SNO differentiation protocol. (Bottom) Brightfield images showing SNO at day (d) 3, 15 and 30 of development. Bright field imaging shows tissue compaction between d3 and d30, a well-known effect as a result of traction forces generated by expanding cell populations. Scale bar, 1 mm. **b** Flow cytometry analysis of autonomic (PHOX2B^pos^) progenitor cells dissociated from SNO and BENO at d15. 3 independent differentiations, 3 tissues per differentiation (*n* = 9). **c** Expression analysis of autonomic progenitor marker (*PHOX2B*, *ASCL1*, *GATA2*) and cortical marker (*PAX6*, *GBX2*) for SNO and BENO (forebrain organoid) at d15 normalized to *GAPDH* expression. SNO and BENO were normalized to undifferentiated stem cell expression. 3 independent differentiations, 4 tissues per differentiation (*n* = 12). **d** Whole-mount immunofluorescence analysis of d15 SNO and BENO stained for sympathetic progenitor marker PHOX2B (green) and TH (red). Scale bar, 500 µm. (Right) Close-up view of PHOX2B expression in SNO vs BENO. Scale bar, 50 µm. **e** Expression analysis of pan-neural marker *TUBB3*, sympathetic markers (*DBH*, *TH*, *SLC18A2*, *SLC6A2*) and peripheral neuron marker peripherin (*PRPH*) for SNO and BENO at d41, normalized to *GAPDH* expression. SNO and BENO were normalized to the expression of undifferentiated stem cells (dashed line). BENO: 3 independent differentiations, SNO: 4 independent differentiations, 4 tissues per differentiation, *n*(BENO) = 12, *n*(SNO) = 16. *PRPH*: *n*(SNO) = 10 *n*(BENO) = 8, *SLC18A2*: *n*(SNO) = 10 *n*(BENO) = 12, *SLC6A2*: *n*(SNO/BENO) = 12, *TUBB3*: *n*(SNO) = 11 *n*(BENO) = 12. **f** (Left) Whole-mount immunofluorescence analysis of d41 SNO and BENO stained for SN marker DBH (gray), TH (red) and NF (green). Scale bar, 1 mm. (Center) Close-up view depicting SNs co-expressing TH and DBH in SNO. Scale bar, 25 µm. **g** LC-MS/MS analysis of BENO and SNO lysates at d41 normalized to the wet weight of the tissues. 3 independent differentiations, 5 tissues per differentiation (*n* = 15). **h** (Left) Representative raster plot of SNO spontaneous activity at 8 weeks. (Right) Detail of a cluster of network bursts from the same recording (boxed in left panel). **i** Time-course of SNO mean firing rate and network burst frequency from weeks 4–8. *n* = 48 SNO from 3 independent differentiations. All data are presented as mean ± s.e.m. Two-tailed unpaired Student’s t-test or Mann-Whitney was used to test statistical significance in (**b**, **c**, **e**, **g**).

SNO generation was achieved by patterning our previously established brain organoid model^14^, BENO, towards sympathetic trunk fate^2,3^. Embedded in a 3D collagen matrix, human iPSCs were differentiated into neural progenitors via dual SMAD inhibition and subsequently patterned toward a caudal identity using the Wnt activator CHIR99021 and retinoic acid. BMP4 and DAPT were included, as in vivo studies have demonstrated their ability to enhance the generation of migrating neural crest cells^31^. For the identification of an optimal protocol for SNO derivation (Fig. 1a), morphogen concentration, treatment window, and cell number had to be fine-tuned (Supplementary Fig. 1a–i). Optimized SNOs comprised 55 ± 1% PHOX2B-positive cells 15 days after initiation of directed differentiation (Fig. 1b and Supplementary Fig. 2a). When compared to BENOs, SNOs showed significantly greater levels of neural crest markers (*PHOX2B*, *ASCL1*, *GATA2*) and diminished expression of forebrain and hindbrain markers (*PAX6*, *GBX2*, respectively) (Fig. 1c, d and Supplementary Fig. 2b). By day 41, trunk neural crest cells differentiated into peripheral (PRPH^pos^) noradrenergic neurons expressing transcripts encoding critical proteins for catecholamine synthesis and reuptake, such as dopamine β-hydroxylase and tyrosine hydroxylase (DBH, TH) as well as dopamine and NA transporters (*SLC18A2*, *SLC6A2*) (Fig. 1e, f and Supplementary Fig. 3a–c). *PHOX2B* expression remained evident in noradrenergic neurons even in later developmental stages (d93, Supplementary Fig. 3d). Both SNOs and BENOs contained similar levels of pan-neural marker *TUBB3* (Fig. 1e), confirming that the enhanced expression of noradrenergic markers in SNOs reflected patterning to a sympathetic neural fate. Furthermore, transcript and immunofluorescence analyses revealed the presence of cholinergic neurons (*CHAT*, *SLC18A3*), glutamatergic neurons (*SLC17A6*), sensory neurons (*BRN3A*), glia (*SLC1A3*) and mesenchymal cells (*PRRX1*), but the absence of motor (*MNX1*) and GABAergic (*GAD1*) neurons (Supplementary Fig. 4).

On day 40, liquid chromatography analysis of SNOs vs BENOs showed significantly higher levels of sympathetic neurotransmitters NA (60.4 ± 6.2 vs 4.4 ± 1.5 pmol/mg) and its precursor dopamine (6.18 ± 1.09 vs 0.51 ± 0.20 pmol/mg), but no enrichment for acetylcholine (18.41 ± 4.91 vs 16.73 ± 4.19 pmol/mg), confirming a predominantly sympathetic nature of the neurons in SNOs (Fig. 1g and Supplementary Fig. 5). SNOs developed functional neuronal networks of increasing spontaneous activity (mean firing rate) and complexity (network burst frequency) that reached a plateau by 6 weeks of differentiation (Fig. 1h, i). Moreover, they demonstrated a concentration-dependent response to nicotine (EC_50_ 29.4 ± 15.4 µM; Supplementary Fig. 6), a hallmark of postganglionic sympathetic neurons. Together with evidence of acetylcholine synthesis—typically confined to preganglionic neurons—these data indicate sympathetic ganglion formation in SNOs. Transcript and whole-mount immunofluorescence analyses of SNO generated from three different iPSC lines indicated a reproducible protocol leading to similar levels of SN markers and spontaneous network activity (Supplementary Fig. 7).

### Generation and characterization of innervated human cardiac muscle (iEHM)

**Fig. 2:**
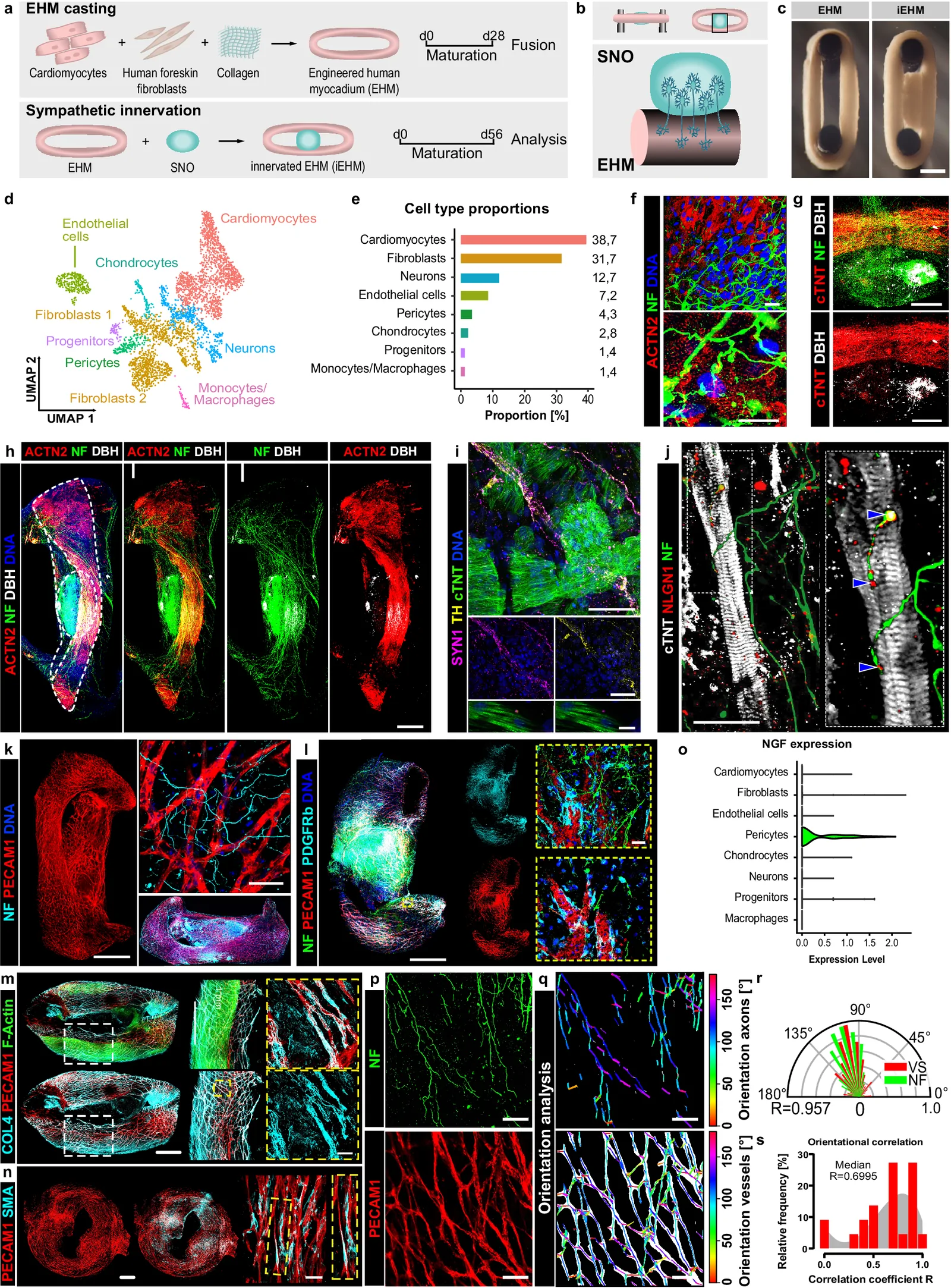
iEHM generation and characterization. **a** A schematic of the iEHM generation workflow. **b** Schematic of sympathetic innervation from the SNO projecting towards the EHM. **c** Brightfield images of EHM and iEHM at 2 weeks of fusion. Scale bar, 1 mm. **d** UMAP embedding and clustering of snRNA-seq profiles from 8-week iEHM (*n* = 3 iEHM, 2 independent differentiations). Representative uniquely per cluster expressed genes are provided in Supplementary Fig. 9. **e** Proportions of the eight major cell types identified in iEHM by snRNA-seq analysis. **f** Whole-mount immunofluorescence (WmIF) close-up view of axons (NF, green) densely innervating CMs (ACTN2, red) of 8-week iEHM. 6 independent experiments. Scale bar, (Top) 20 µm, (Bottom) 10 µm. **g** Detail of the iEHM mid-section showing a cluster of DBH (gray) and NF (green)-positive SN in close proximity to the myocardium (cTNT, red). Scale bar, 500 µm. 3 independent experiments. **h** Overview WmIF images stained for CM marker ACTN2 (red), neural marker NF (green), sympathetic marker DBH (gray) and DNA (blue). Scale bar, 1 mm. 3 independent experiments. **i** IF-staining of sympathetic (TH, yellow) presynaptic structures (SYN1, magenta) distributed throughout the myocardium. Scale bar, 50 µm. (Bottom) Close-up view shows co-localization of SYN1 and TH on CMs (cTNT, green). Scale bar, 5 µm. 3 independent experiments. **j** (Left) IF-staining of iEHM for cTNT (gray), post-synaptic marker NLGN1 (red) and NF (green). Scale bar, 20 µm. (Right) Close-up view showing synaptic bouton-like varicosities co-stained with NLGN1 on the surface of CMs, indicated by blue arrowheads. 2 independent experiments. **k** WmIF of 8-week iEHM stained for endothelial cell marker PECAM1 (red) and NF (cyan). Scale bar, 1 mm. (Top right) Close-up view of vessel-like structures interlaced by neuronal axons. Scale bar, 50 µm. 6 independent experiments. **l** WmIF of 8-week iEHM stained for DNA (blue), PECAM1 (red), NF (green), and PDGFRβ (cyan). Scale bar, 1 mm. ROI: Representative image of pericytes (cyan) lining vascular structures (red), (Top) Maximum intensity projection, (Bottom) Single plane. Scale bar, 20 µm. 3 independent experiments. **m** WmIF image of 6-week iEHM stained for PECAM1 (red), basement membrane marker COL4 (cyan), and myocyte marker f-Actin (green). Scale bar, 1 mm. (Center) Region of interest showing endothelial structures co-localizing with COL4 (Right). Close-up view. Scale bar, 50 µm. 2 independent experiments. **n** WmIF of 6-week iEHM stained for PECAM1 (red) and smooth muscle marker SMA (cyan). (Right) Close-up view of SMA-positive mural cells (cyan) colocalizing with endothelial structures (red). Scale bar, 50 µm. ROI shows SMA-positive mural cells in direct contact with endothelial cells. 2 independent experiments. **o** Violin plot showing contribution of iEHM cell types to nerve growth factor (*NGF*) expression. **p** Example regions analyzed in orientation analysis, stained for axons (NF, green) and endothelial structures (PECAM1, red). Scale bar, 75 µm. **q** Skeletonized binary masks of vasculature and axons from (**p**). The color of the segments illustrates their orientation. Scale bar, 75 µm. **r** Distribution of orientation angles for vasculature (VS) and axons (NF) from **(p**), including Pearson correlation coefficient R. **s** Histogram of correlation coefficient distribution for all analyzed regions (25 images, *n* = 3 iEHM). Median R suggests robust orientation correlation.

To generate the neurocardiac interface, the cardiac module (EHM) was fused with the neural module (SNO). The cardiac module was produced by embedding differentiated CMs of predominantly ventricular fate and human foreskin fibroblasts in a collagen matrix^17^. Since MEA data suggested a stabilized neuronal network function 6 weeks of SNO culture (Fig. 1h, i), a 40-day-old SNO was positioned between the two arms of a 4-week-old EHM, allowing the tissues to fuse (Fig. 2a–c). The resulting innervated EHM (iEHM) displayed spontaneous, synchronous contractions as early as two days after fusion, indicating that the presence of the SNO did not impair EHM function (Supplementary Movie 1).

To define iEHM cell composition, we isolated nuclei (Supplementary Fig. 8) and performed single-nuclei RNA sequencing (snRNAseq) of three iEHM 6 weeks after fusion. Leiden clustering revealed 8 distinct populations (Fig. 2d and Supplementary Fig. 9). iEHM consisted of 39% CMs, 32% fibroblasts, 13% neurons, 7% endothelial cells, 4% pericytes, 3% chondrocytes, 1% progenitors, and 1% monocytes/ macrophages (Fig. 2e). The neuronal population comprised autonomic noradrenergic neurons marked by * ASCL1*, *PHOX2B*, *PRPH*, *HOXD8*, *TH*, and *SLC18A2*; autonomic cholinergic neurons marked by *PHOX2A*, *PRPH*, and *CHAT*; and an intermediate population co-expressing cholinergic and noradrenergic markers (Supplementary Fig. 10a, b). The latter population co-expressing *TH, CHAT, GATA3* and *ISL1* could represent a transitional developmental stage and was observed only in one out of three tissues. Beyond peripheral autonomic neurons, we identified glutamatergic neurons expressing *SLC17A6*, as well as *PRPH*-negative neurons (Supplementary Fig. 10a, b). Normalized HOX gene expression across neuronal clusters showed that *PHOX2A/B* marked the autonomic lineage, whereas *HOXA3* and *HOXB3* defined vagal neural crest identity and *HOXB5* and *HOXD8* indicated a more posterior trunk neural crest identity (Supplementary Fig. 10c). Cell identity was further substantiated by the detection of cell-type characteristic sodium, potassium, and calcium channels (Supplementary Fig. 11). CMs could be subclustered into ventricular-like (56%) and atrial/pacemaker-like clusters (29%) while 15% of the cells presented an indistinguishable identity (Supplementary Fig. 12a, b). The present study did not include additional experiments to distinguish whether the observed cardiomyocyte heterogeneity is line-specific or protocol-dependent, although such mixed atrial-, ventricular-, and pacemaker-like populations are commonly observed following standard Wnt-modulation-based differentiation^32,33^. Expression of *SHOX2*, *FGF12*, *HCN4*, *TBX18*, *MYH11*, and *ZNF385B* within the atrial CMs suggested the presence of pacemaker-like cells. Moreover, one of the two fibroblast populations (*COL1A1*, *COL1A2*, *COL3A1*) in iEHM stemmed from SNO-derived neural crest cells and was characterized by *FGF14*, *PAX3*, *ZIC1*, and *HOXB3* expression (Supplementary Fig. 12c, d).

8 weeks after fusion, immunofluorescence analysis showed an extensive sympathetic axonal network marked by neurofilament (NF) and DBH extending towards cardiac muscle expressing alpha-actinin (ACTN2) or cardiac troponin T (cTNT); higher magnification images showed neurons interlacing CMs (Fig. 2f–h, Supplementary Fig. 13a and Supplementary Movie 2). To visualize axonal synaptic varicosities in close proximity to CMs, we stained for the pre-synaptic marker synapsin (SYN1), while TH served as a marker for noradrenergic neurons. SYN^pos^-TH^pos^ noradrenergic varicosities were found in close proximity to CMs (Fig. 2i and Supplementary Movies 3–7), suggesting potential for neurocardiac communication. Furthermore, neuroligin 1, a postsynaptic marker expressed also by CMs in the human heart^34^ (Supplementary Fig. 13b) localized on CMs close to pre-synaptic varicosities (Fig. 2j) as has been previously shown for other proteins such as β-adrenoreceptors^35^.

### Neuro-vascular network development in iEHM

Since not all SYN^pos^-TH^pos^ noradrenergic axonal boutons were found in close proximity to CMs (Supplementary Movie 8), we reasoned that SN may interact with other cell populations co-developing in iEHM, such as vascular cells. Indeed, immunofluorescence analysis of 6 weeks after fusion iEHM validated the presence of an extensive vascular network consisting of PECAM1-positive endothelial cells, interlaced by neurons (Fig. 2k and Supplementary Movies 9–11). Note that the origin of the vascular network is both from the SNO and the EHM component; the latter deriving from CM-enriched directed differentiations from iPSC^17^. Such populations comprise approximately. 2% CD31-positive endothelial cells, which, if not selected out by, for example, metabolic selection^36^ can form capillary-like structures (Supplementary Fig. 14a) under standard EHM culture conditions^17^. SNO contained sparse endothelial cells, with one out of four tissues developing a vascular network when tissues were maintained in SNO media. Culturing SNOs in iEHM medium, which contains vascular endothelial growth factor, led to extended endothelial network development in all SNOs (Supplementary Fig. 14b), suggesting that SNO and iEHM vascularization is supported by VEGF. In iEHM, we observed both capillary-like structures (diameter 5–10 µm) as well as vessels with diameters between 20–40 µm (Supplementary Fig. 14c, d). Irrespective of their size, vessels were surrounded by a basal lamina layer (COL4^pos^), pericytes (PDGFRb^pos^) and vascular smooth muscle cells (SMA^pos^) (Fig. 2l–n and Supplementary Fig. 14a). Interestingly, similar to the native heart^28^, pericytes were identified as the primary source for nerve growth factor (NGF) in iEHM, a neurotrophin responsible for chemoattracting cardiac SN (Fig. 2o). In line with this data, directionality analysis using deep learning-based segmentation showed a correlation between the orientation of neuronal axons and vessels in iEHM (Fig. 2p–s).

### Autonomic neurons functionally connect with CMs in iEHM

**Fig. 3:**
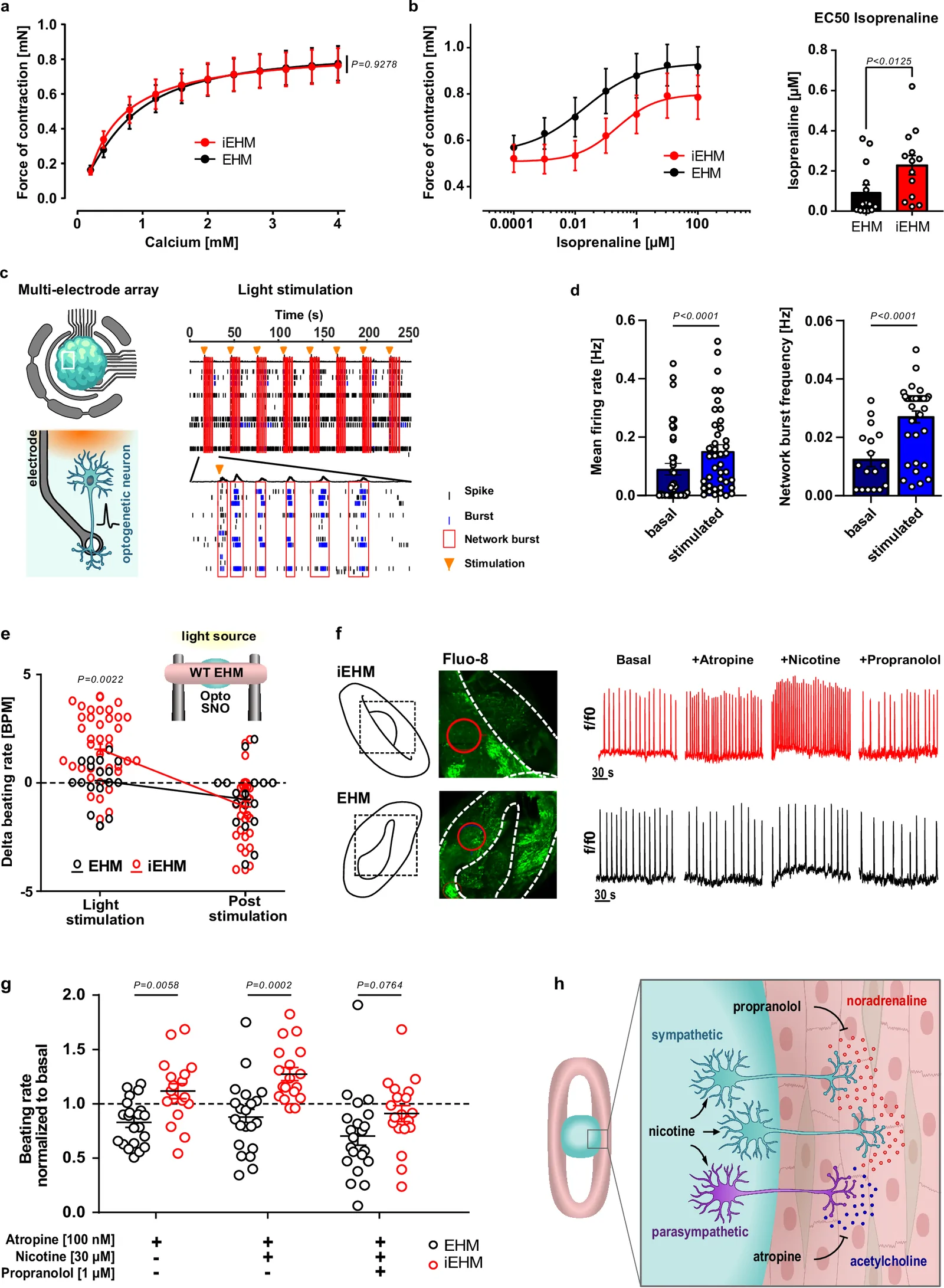
Functional analysis and connectivity between neurons and cardiac cells in iEHM. **a** Force of contraction (FOC) recorded under increasing calcium concentrations and electric pacing at 1.5 Hz; 8 weeks after fusion. Three independent differentiations, 6–8 tissues per differentiation (*n* = 22). **b** (Left) FOC response to escalating concentrations of isoprenaline recorded at EC_50_ of calcium and electrical stimulation at 1.5 Hz, 8 weeks after fusion. (Right) Comparison of the EC_50_ of isoprenaline between EHM (*n* = 13) and iEHM (*n* = 14) from the same experiment. Two independent differentiations, 6–7 tissues per differentiation. **c** (Left) Schematic of optogenetic stimulation of SNO mounted to MEA, (Right) Example raster plot showing light-induced activity of 8 weeks SNO. Light stimulation pulses are indicated with orange triangles. **d** (Left) Endpoint (8 weeks) measurement of basal and light-stimulated mean firing rate of optogenetic SNO. 3 independent differentiations, 12–15 tissues per differentiation (*n* = 41). (Right) Measurement of basal and light-stimulated network burst frequency of optogenetic SNO on the same date. n(stimulated) = 36, n(unstimulated) = 17, 3 independent differentiations. **e** Schematic: Light stimulation of optogenetic SNO (opto SNO) leads to stimulation of the wildtype (WT) EHM in iEHM. Measurement of differences in beating rate upon light stimulation. n(EHM) = 15, n(iEHM) = 39, 3 independent differentiations, age = 5–10 weeks of fusion. **f** (Left) Representative confocal image of live recordings stained with calcium-sensitive dye Fluo-8 (green), including regions of interest (red). (Right) Traces of mean fluorescence intensity of Ca^2+^-indicator dye Fluo-8 of iEHM and EHM upon pharmacological stimulation (more details in Supplementary Fig. 16b). **g** Quantification of EHM and iEHM beating rate during sequential treatment with 100 nM atropine, 30 µM nicotine, and 1 µM propranolol. n(EHM) = 21, n(iEHM) = 19 (8 out of 27 iEHM were not responding, more details in Supplementary Fig. 16a), 4 independent differentiations, age = 8–10 weeks of fusion, Beating rate of the groups is normalized to the individual basal beating rate (indicated by dashed line). Data are displayed as bar graphs with mean ± s.e.m. as well as all individual data points. **h** Schematic of pharmacological stimulation of iEHM: Nicotine stimulates neurotransmitter release from sympathetic and parasympathetic neurons in the iEHM. The effect of acetylcholine is inhibited by atropine administration, and the effect of NA is inhibited by the non-specific β-adrenoreceptor blocker propranolol. 2-way ANOVA with Sidak’s multiple comparisons post hoc test was used to test statistical significance in (**a,** **e**). Two-tailed unpaired Student’s t-test or Mann–Whitney test was used for (**b**, **d-right**, **g**), and two-tailed Wilcoxon test was used for (**d-left**). BPM = beats per minute, EHM = engineered human myocardium, f/f0 = ratio of fluorescence intensity, iEHM = innervated EHM, SNO = sympathetic neural organoid.

Since iEHM contained noradrenergic axonal bouton-like structures proximal to CMs, we reasoned that the SNs have an impact on tissue function and may form functionally relevant neuro-cardiac junctions (NCJ). First, we investigated the contractile performance of iEHM vs EHM, 6 weeks after fusion. Isometric force measurements under electrical stimulation at 1.5 Hz showed no difference in iEHM and EHM contractile performance (Fig. 3a), suggesting little effect of autonomic neurons in tissue contractility. Moreover, when stimulated with the inotropic agent isoprenaline, iEHM presented β-adrenoreceptor desensitization in comparison to EHM with significantly higher EC_50_ values (0.23 ± 0.05  vs 0.10 ± 0.03 µM, respectively) (Fig. 3b). β-adrenoreceptors sensitivity to isoprenaline is inversely correlated to the presence of autonomic neurons^37^ and NA in vivo^38^, indicating that CMs sense NA secreted in the iEHM.

Next, we investigated the effect of autonomic neurons on iEHM chronotropy by optogenetic stimulation. We utilized a neuronal-specific optogenetic iPSC line in which the red-shifted variant of channel rhodopsin f-Chrimson was integrated into the *AAVS1* locus under the human synapsin promoter (pSYN_fChrimson)^39^. Opto-SNOs showed higher firing rate and network burst frequency under light stimulation by week 8 of culture (Fig. 3c, d). iEHM generated by fusing opto-SNOs with wild-type EHMs was compared to standard EHMs, to rule out unspecific activation of CMs by light or heat (Fig. 3e). Light stimulation of optogenetic iEHMs evoked a positive chronotropic response up to two-fold of baseline (Supplementary Movie 12). Beating rate analysis of 42 iEHMs (*N* = 3) showed a mean beating rate increase of 24 ± 4% in iEHMs vs 1 ± 3% in EHMs, providing proof for functional connectivity between the autonomic neurons and pacemaker-like cells (Fig. 3e). Pacemaker-like cells expressing *SHOX2*, *FGF12*, *TBX18*, *MYH11*, *ZNF385B*, and *HCN4*, a marker essential for pacemaker activity in the embryonic heart^40^, were identified by snRNAseq (Supplementary Figs. 12b and 15a) and immunofluorescence (Supplementary Fig. 15b).

Furthermore, to delineate the contribution of the sympathetic and a potential parasympathetic component to iEHM beating response, we utilized classical pharmacological interventions (Fig. 3f–h): (1) to block muscarinic receptors and thus ACh released by parasympathetic neurons (100 nM atropine), (2) to activate postganglionic nicotinic acetylcholine receptors on sympathetic or parasympathetic neurons (30 µM nicotine) and (3) to block postsynaptic β-adrenoreceptors (1 µM propranolol) expressed on pacemaker-like cells. The lack of response to atropine in control EHM tissues showed that in the absence of autonomic neurons, atropine does not directly affect cardiac muscle beating rate. In contrast, iEHM showed a slight positive chronotropic response to atropine, suggesting the blockade of parasympathetic NCJ. Since nicotine would stimulate both sympathetic and parasympathetic neurons, we reasoned that pre-incubation with atropine would block the action of ACh and allow us to evaluate the absolute effect of SN activation only. Indeed, the addition of nicotine in the presence of atropine significantly increased beating rate by 38 ± 13% of baseline (*n* = 19, *N* = 5). In the native tissue, postganglionic SN upon nicotine stimulation would release NA that would modulate chronotropy^41^ by binding to β-adrenoreceptors in pacemaker-like cells. Blockade of β-adrenoreceptors by propranolol abrogated the chronotropic effects of nicotine, providing further evidence for functional NCJ in iEHM. 19 out of 27 iEHM responded to these stimulations, while 8 iEHM showed no response (Supplementary Fig. 16), suggesting that catecholamine release and tissue level diffusion were insufficient to reach spontaneously beating CMs or pacemaker-like cells in iEHM.

To examine cell-state adaptations due to functional connectivity, we performed bulk RNA-seq comparing iEHM with matched controls in which EHM and SNO were cultured independently for the same duration in iEHM medium and pooled at collection to include both neural and cardiac components (Supplementary Fig. 17a). Bulk RNA-seq analysis revealed distinct transcriptional programs in iEHM, including upregulation of pathways related to immune cell regulation, metabolic remodeling, and CM hypertrophy (Supplementary Fig. 17b, c and Supplementary Fig. 18). Whole-mount immunofluorescence showed increased presence of hematopoietic progenitors (CD45^pos^) and immune cells (CD68^pos^) in iEHM compared to EHM (Supplementary Fig. 17d, e). CM size analysis using a validated flow cytometry-based approach^17^ revealed a modest but significant increase in iEHM CM size, accompanied by elevated expression of adrenergic receptors (*ADRB1*, *ADRB2*) and *GJA1* (Supplementary Fig. 17f, g). Immunostaining confirmed enhanced CX43 puncta density in iEHM CMs (Supplementary Fig. 17h, i). Transcriptomic data further indicated a metabolic shift towards fatty acid oxidation, with higher levels of *PDK4, NR4A1–3*, fatty acid transporter *CD36*, and lower levels of glucose transporters *SLC2A1* and *SLC2A4* (Supplementary Fig. 17b, c, j), consistent with noradrenergic signaling–induced metabolic reprogramming^42–45^.

### Autonomic neuron hyperactivity contributes to pro-arrhythmic phenotype in *KCNH2* mutant iEHMs

**Fig. 4:**
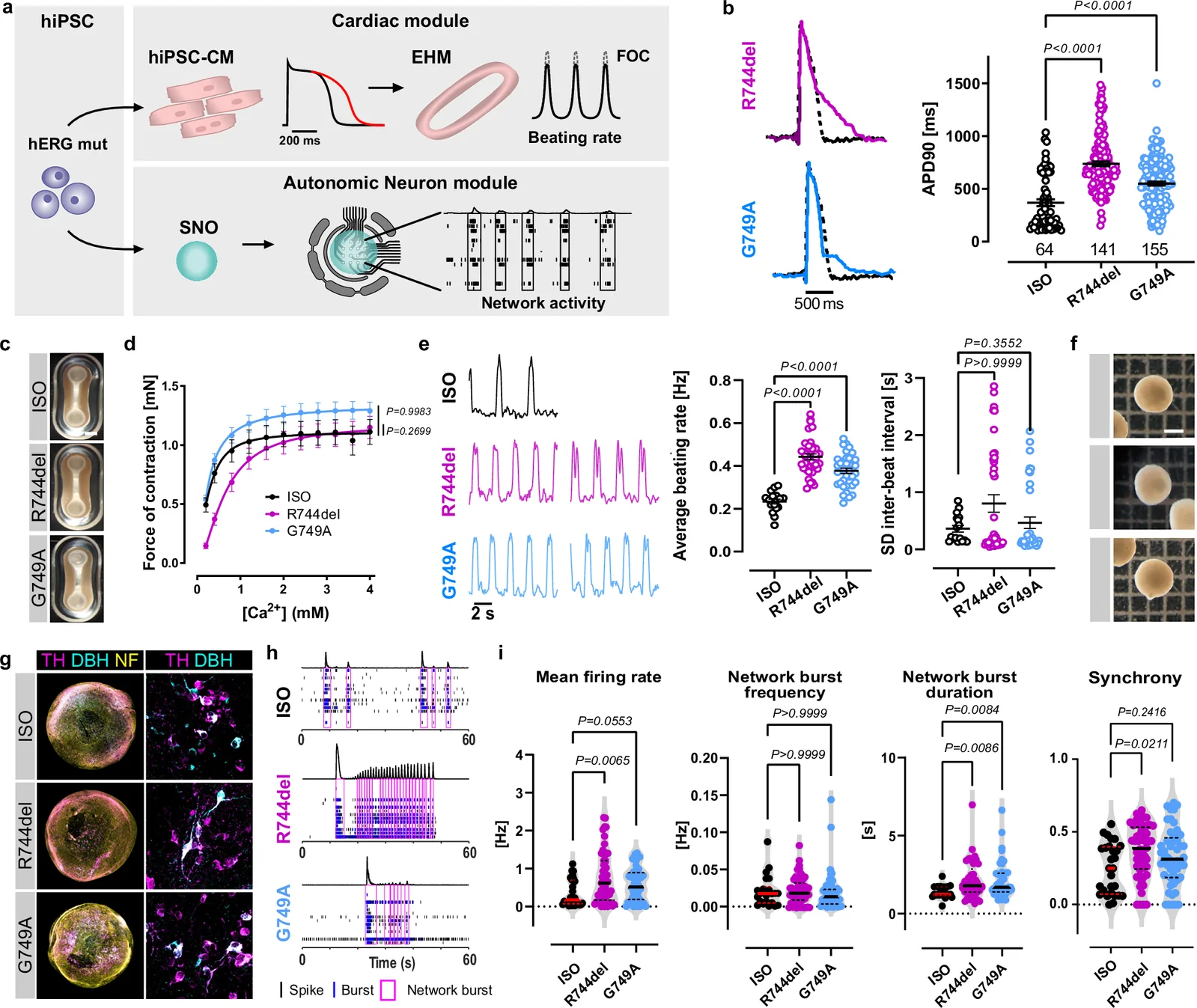
Modeling *KCNH2*-dependent electrical network dysregulation in individual cardiac and neural modules. **a** A schematic depicting the analysis workflow of hERG-mutant hiPSC-derived cardiac and neural iEHM modules. **b** (Left) AP morphology of p.R744del (purple) and p.G749A (blue) compared to isogenic control (ISO, dashed black line). (Right) AP durations of CMs at 90% repolarization (APD90) from hERG-mutant lines and isogenic control. Individual data points represent measurements from single cells of three independent differentiations. **c** Brightfield images at d14 show similar EHM morphology for all lines. Scale bar, 1 mm. **d** FOC measured upon increasing calcium (Ca^2+^) concentrations and 1.5 Hz electrical pacing frequency at 8 weeks of culture. *n*(ISO) = 14, *n*(p.R744del) = 29, *n*(p.G749A) = 31. **e** (Left) Example traces of spontaneous beating of 6-week-old EHM showing both normal and after-depolarizations in p.R744del and p.G749A mutations. (Center) Average beating rate of hERG-mutant EHM and the isogenic control between 4 and 8 weeks of culture. (Right) Analysis of standard deviation (SD) of inter-beat-intervals (R-R intervals) as a measure of beating rate variability of hERG-mutant and isogenic control EHM between 4 and 8 weeks of culture. **f** Brightfield images of d40 SNOs show similar morphology for all lines. Scale bar, 1 mm. **g** WmIF staining of SN marker DBH (cyan), TH (magenta), and NF (yellow) of d43 SNOs. Scale bar, 500 µm. Close-up view of the same organoid details SN co-staining of DBH and TH. Scale bar, 30 µm. **h** Example raster plots showing spontaneous electrical field potentials of SNOs from all hiPSC lines between d80-d90. **i** Mean firing rate, network burst frequency, network burst duration, and synchronicity index of hERG-mutant and isogenic control SNOs averaged between d80-d120 of culture. Individual data points represent averages from individual SNOs from 3 independent differentiations. An ordinary one-way ANOVA or Kruskal-Wallis test with Dunn’s multiple comparisons post hoc test was used in (**b**, **e**, **i**). Two-way ANOVA with Dunnett’s multiple comparison post-hoc test in (**d**).

Although *KCNH2* mutations lead to seizures in the brain and LQT2 syndrome in the heart, it is unclear whether autonomic neuron function is impacted and how the latter contributes to hERG-related arrhythmia. Since *KCNH2* is expressed in both SNs and CMs, we reasoned that iEHM would allow us to define the autonomic neuron contribution to *KCNH2-*related cardiac dysregulation. Thus, we analyzed SNOs, EHMs and iEHMs from two iPSC lines genetically engineered to harbor mutations in amino acids of *KCNH2* essential for its function^46^ (Fig. 4a).

Mutant iPSC lines (2 clones per mutant) were differentiated to SNs and CMs as efficiently as the isogenic control with no significant differences in *KCNH2* transcript / protein levels and distribution of (Supplementary Figs. 19 and 20). *KCNH2* levels increased significantly between d40 and d70 (Supplementary Fig. 20a), consistent with earlier reports^47^. Action potential duration by voltage mapping showed a significant delay in repolarization due to a reduced potassium current of mutant compared to isogenic CMs (Fig. 4b) which in some cases led to after depolarization (AD)-like events (10% p.R744del vs 7% p.G749A vs 4% Isogenic, Supplementary Fig. 21). At the tissue level, EHM mutants showed similar morphology and force of contraction to isogenic EHM (Fig. 4c, d). However, mutant EHM presented significantly higher beating rates, with a subset of R744del tissues presenting AD-like events and higher beat-to-beat variability (Fig. 4e).

Next, SNO generated from the respective lines showed similar morphology and SNs development (Fig. 4f, g). However, electrical network recordings revealed a hyperactive phenotype of mutant SNO, with increased mean firing rates, synchronicity, network burst frequency, and duration (Fig. 4h, i). As observed in CMs, the p.R744del mutation showed a stronger phenotype, compared to p.G749A, represented by a significant change in network parameters. This data shows that, besides the network dysregulation in the brain, peripheral autonomic neurons are also hyperactive due to *KCNH2* mutations.

**Fig. 5:**
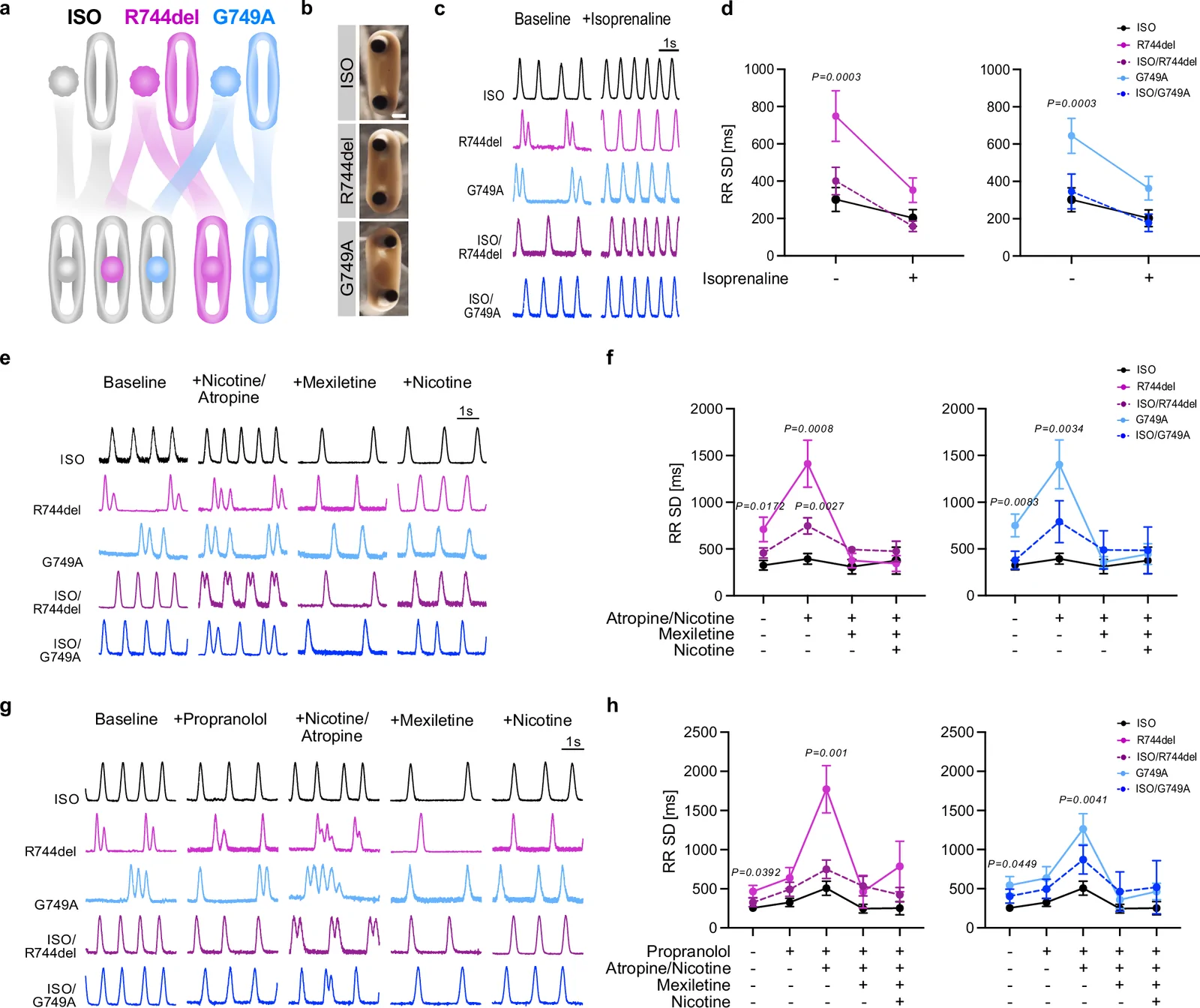
Modeling *KCNH2*-dependent electrical network dysregulation in iEHM. **a** A schematic of the “mix & match” strategy for the fusion of isogenic and mutant iEHM. **b** Brightfield images of iEHM at d39 after fusion. Scale bar, 1 mm. **c** Example traces of (Left) spontaneous beating and (Right) 1 µM isoprenaline of 6-week-old fused iEHM at EC_50_ Ca^2+^. **d** Beat-to-beat variability (RR SD) of (Left) R744del mutant (Right) G749A mutant iEHM (EHM/SNO) compared to isogenic upon isoprenaline treatment in the first 30 s of the treatment. 3 independent differentiations, *n*(ISO) = 24, *n*(p.R744del) =  28, *n*(ISO/p.R744del) = 28, *n*(p.G749A) = 19, *n*(ISO/p.G749A) = 17 tissues. **e** Example traces of (Left) spontaneous beating, (Middle left) stimulated with 30 µM nicotine in the presence of 100 nM atropine, (Middle right) blocked with 50 µM mexiletine and (Right) acutely stimulated with 30 µM nicotine of 6-week-old fused iEHM at EC_50_ Ca^2+^. **f** Beat-to-beat variability (RR SD) of (Left) p.R744del mutant (Right) p.G749A mutant iEHM compared to isogenic upon nicotine treatment in the presence of atropine, followed by mexiletine and nicotine treatments. Atropine and nicotine measurements were measured in the first 30 s of the treatment, and mexiletine was measured in the last 60 s. 3 independent differentiations (Baseline and Atropine/Nicotine treatments: 3 differentiations, Mexiletine and Nicotine treatments: 2 differentiations). *n*(ISO) = 27, *n*(p.R744del) = 31, *n*(ISO/p.R744del) = 28, *n*(p.G749A) = 17, *n*(ISO/p.G749A) = 17.**g** Example traces of (Left) spontaneous beating, (Middle left) blocked with 1 µM propranolol (Middle) stimulated with 30 µM nicotine in presence of 100 nM atropine, (Middle right) blocked with 50 µM mexiletine and (Right) acutely stimulated with 30 µM nicotine of 6-week-old fused iEHM at EC_50_ Ca^2+^. **h** Beat-to-beat variability (RR SD) of (Left) p.R744del mutant (Right) p.G749A mutant iEHM compared to isogenic upon blocking by propranolol, nicotine in the presence of atropine, followed by mexiletine and nicotine treatments. Atropine and nicotine responses in the first 30 s of the treatment, propranolol and mexiletine were measured in the last 60 s. 3 independent differentiations (Baseline, Propranolol and Atropine/Nicotine treatments: 3 differentiations, Mexiletine and Nicotine treatments: 2 differentiations). *n*(ISO) = 26, *n*(p.R744del) = 26, *n*(ISO/p.R744del) = 27, *n*(p.G749A) = 18, *n*(ISO/p.G749A) = 17. The individual data points for each iEHM RR-SD and average beating rate are presented in Supplementary Fig. 22 (**b**–**g**). Two-way ANOVA with Dunnett multiple comparisons post hoc test was used for (**d**, **f**, **h**).

To identify the impact of *KCNH2* mutation in iEHM and the contribution of SNO hyperactivity to this phenotype, iEHMs were generated by a “mix&match” approach (Fig. 5a). iEHMs displayed no difference in tissue morphology (Fig. 5b). To quantify after depolarizations and irregular beating behavior we analyzed beat-to-beat variability as standard deviation of the R-R interval (RR-SD, Supplementary Fig. 22a). We reasoned that afterdepolarizations would lead to increases in RR-SD, whereas regular beating would be presented with low RR-SD.

First, we stimulated iEHM with isoprenaline, typically used to emulate neuronal stimulation of cardiac cultures. Isoprenaline led to an increase in beating rate (Fig. 5c and Supplementary Fig. 22b) but induced no irregular beating, leading to a decrease in RR-SD (Fig. 5d and Supplementary Fig. 22c).

To mimic increased ANS stimulation from the brain during a seizure, we applied nicotine in the presence of atropine to isolate the impact of SN activation in our system. SN stimulation by nicotine increased significantly beat-to-beat variability in iEHM of _mut_EHM – _mut_SNO (for both p.G749A and p.R744del) but had no effect on the respective isogenic control tissues (Fig. 5e, f). Moreover, hybrid tissues (_iso_EHM – _mut_SNO) showed that the pronounced autonomic hyperactivity in p.R744del SNOs led to a lesser but significant increase in the beat-to-beat variability in iEHMs compared to complete mutant tissues, suggesting that neuronal dysregulation, depending on the mutant, can exacerbate the pro-arrhythmic phenotype irrespective of the CM status. Treatment of the same tissues with mexiletine, an antiarrhythmic drug that inhibits both neuronal and CM excitation by blocking sodium channel^48^ was able to rescue the observed phenotype (Fig. 5e, f). Given that nicotine was shown to elicit only a transient beating rate increase in co-cultures of sympathetic ganglia and CMs^49^, we repeated nicotine application following mexiletine exposure. Although nicotine significantly increased beating rate compared to mexiletine alone (Supplementary Fig. 22d), suggesting NA release, no afterdepolarizations were observed (Fig. 5e, f and Supplementary Fig. 22e)^43^.

To assess whether the observed increase in beat-to-beat variability was mediated by noradrenaline release, we repeated the experiments in the presence of the β-adrenergic blocker propranolol. In contrast to the sodium-channel inhibitor mexiletine, propranolol failed to reduce the number of afterdepolarizations induced by nicotine-mediated activation of autonomic neurons (Fig. 5g, h and Supplementary Fig. 22f, g). Significant decrease of beating rate and contractile force in the presence of propranolol, however, indicated successful β-adrenoreceptor blockade (Fig. 5g and Supplementary Fig. 22f, h).

Altogether, this data demonstrates that hERG loss-of-function mutations cause not only cardiomyocyte but also autonomic neuron dysfunction, and both contribute to a proarrhythmic phenotype. Notably, β-adrenoceptor blockade alone was insufficient to rescue this phenotype, whereas treatment with mexiletine, targeting both neuronal and cardiomyocyte excitability, proved to be more effective.

## Discussion

The brain communicates with the heart via the autonomic nervous system. Although increasing evidence suggests that channelopathies or miscommunication between brain and heart underlie a number of diseases^5,6,50^ including SUDEP, our understanding of human neuro-cardiac cell interaction is limited. Here, we established an innervated engineered heart muscle and used this system to study human neuro-cardiac coupling at structural, molecular and functional levels.

To study the functional interaction between autonomic neurons and cardiac cells, monolayer co-culture systems^2,24,51^, as well as microfabricated devices^22,23^ have been employed. As a cell source, isolated primary cells were co-cultured with human iPSC-derived cells, while only recently human-derived co-cultures were developed^22,24–26^. Supplementary Table 1 provides an overview of published models for human SN derivation^2,3,22,24–26,52–56^. In 2D cultures, neurotransmitter measurements frequently report dopamine levels exceeding NA by ~10–100-fold^2,52,54^. Since dopamine generation is rate-limiting for NA synthesis, elevated dopamine may indicate immature noradrenergic specification (low dopamine β-hydroxylase, DBH) or a dopaminergic neuron phenotype. In contrast, SNO exhibits ~10-fold NA than dopamine, consistent with robust noradrenergic identity. While 2D systems capture direct cell–cell interactions with excellent experimental control, 3D organoids and engineered tissues provide complementary strengths, including spatial organization, multicellular self-assembly, tissue dynamics, and maturation features resembling the native tissue^57,58^. Recently, a cardiac gastrula organoid was developed in which peripheral neurons (sensory and motor neurons) co-develop with anterior foregut and cardiac structures^59^. In parallel to iEHM development, two neurocardiac spherical organoid models were reported that provided evidence for functional connectivity between neurons and cardiomyocytes^25,26^. In these models, noradrenaline or acetylcholine levels were not quantified^25^ or were 100-fold lower^26^ than in SNO. Here, we bioengineered iEHM, leveraging a ring-shaped cardiac tissue format that can be mounted on auxotonic stretchers, enhancing cardiomyocyte alignment, training, and maturation^17^. In this format, the iEHM allows beat rate analysis and enables parallel measurement of contractile force ^17,60^. Force of contraction measurements revealed β-adrenoceptor desensitization to isoprenaline—classically associated with chronic noradrenaline exposure^37^—implying that CMs sense NA released from sympathetic terminals in the iEHM. A systematic transcriptomic benchmarking analysis across studies would be an important future direction.

In this study, single-nucleus profiling was not performed on isolated SNOs; instead, we focused on the fused iEHM, as our main objective was to define the cellular composition of the final innervated model used for functional experiments. Single-nuclei sequencing revealed that iEHM consisted of 39% CMs, 32% fibroblasts, 13% neurons, 7% endothelial cells, 4% mural cells, 3% chondrocytes, and 1% immune cells. These levels were partially comparable to adult heart tissue^34^, in which cardiomyocytes ranged from 30.1–49.2%, fibroblasts from 15.5–24.3%, mural cells from 17.1–21.2%, endothelial cells from 7.8–12.2%, immune cells from 5.3–10.4%, and 2% neural cells. Thus, although iEHM does not reproduce adult heart composition in full, it captures key populations within a human multicellular system. iEHM is not intended to fully recapitulate the exact cellular composition of native human myocardium. Rather, it is designed as a tractable human multicellular system to study neuro-cardiac interactions in a controlled setting.

CMs segregated into ventricular-like (56%) and atrial/pacemaker-like (29%) subclusters, while the remaining 15% could not be assigned a clear subtype identity. This distribution is consistent with prior reports showing that standard Wnt-modulation-based differentiation commonly yields mixed atrial-, ventricular-, and pacemaker-like CM populations^32,33^. In future iterations of this model, subtype-directed CM differentiation may represent a valuable strategy to improve CM population specificity.

Single-nuclei sequencing of the neuronal clusters of iEHM showed that around half of the neuronal population displayed autonomic identity, including noradrenergic, cholinergic and mixed cholinergic/noradrenergic populations. Neuronal integration influenced cardiac tissue state. The increased abundance of immune cells in iEHM vs EHM was particularly interesting as it suggests neuro-immune interactions. Cardiomyocytes in iEHM were larger than in EHM, consistent with the trophic effects of noradrenergic signaling^26,61^. This was accompanied by altered expression of *PDK4* and *NR4A3*, consistent with a shift toward fatty-acid oxidation^42–45^, as well as increased β-adrenoceptor abundance and higher levels of structural and gap-junction proteins^59,60^. Together, these findings support neuron-dependent maturation of the cardiac tissue. Similar effects on cardiomyocyte size and gap-junction expression have been recently reported in another neuro-cardiac system^26^.

Resembling the epicardial innervation pattern of the native heart, iEHM innervation density showed an inverse correlation to tissue depth. Interestingly, neuronal axons and presynaptic axonal boutons in iEHM were found in close proximity to capillary-like structures, which is in line with in vivo observations^28^. The vascular network observed in iEHM consisted of capillary-like (diameter 5–10 µm) and venous-like (diameter 20–40 µm) structures. The vascular cells originated from both the cardiac component (EHM), which contained endothelial cells as a byproduct of mesoderm/cardiac differentiation, and the neural component (SNO). The development of endothelial cells in the SNO is unsurprising since cKIT-positive endothelial cells have been shown to emerge during cardiac neural crest development in vivo due to transient BMP4 expression^62^. Similarly, SNOs are submitted to transient BMP4 exposure during the commitment phase. Another interesting point about the co-development of vessels and neurons is that during development, mural cells such as pericytes and smooth muscle cells produce and secrete NGF, which chemoattracts noradrenergic axons and guides sympathetic innervation of the heart^28^. Comparably, in iEHM, pericytes surrounding the dense capillary network were found to be the main source of NGF. Beyond development, NGF is considered one of the key factors orchestrating neuronal homeostasis in the adult heart. After myocardial infarction, NGF dysregulation is hypothesized to significantly contribute to neuronal remodeling, leading to sympathetic overdrive and arrhythmias^63^. This data suggests that iEHM can be employed to study developmental mechanisms, which in some cases are reactivated in the diseased heart.

As SNs innervated the deeper layers of the EHM, TH-positive synaptic varicosities were found in close proximity to CMs or pacemaker-like cells, as previously observed in human postmortem heart samples and co-cultured cells^35^. Since immunofluorescence analysis suggested the presence of NCJs, we assessed functional coupling between SNs and CMs. By 6 weeks post-fusion, iEHM exhibited contractile performance comparable to EHM, indicating that neither the neuronal module nor the culture conditions impaired myocardial function. From 6 weeks after fusion, optogenetic and pharmacological interventions modulated iEHM beating rates, demonstrating evidence for functionally relevant NCJs. The moderate but consistent changes in beating rate may reflect low levels of pacemaker-like cells or immature contracting CM present in the tissue. Responsiveness to atropine indicated cholinergic input, while the absence of *MNX1* in SNOs argued against spinal motor neurons being the source of acetylcholine. Indeed, the cholinergic population likely comprises postganglionic parasympathetic neurons of cardiac neural-crest origin since snRNAseq showed CHAT^pos^ populations also co-expressing *PRPH*, *PHOX2A*, *HOXB3*, *HOXA3* and nicotinic acetylcholine receptor *CHRNA3*, typically expressed in autonomic postganglionic neurons. In contrast, in line with a trunk neural crest commitment^52,64,65^, *HOXD8* and *HOXB5* were expressed in the noradrenergic population co-expressing sympathetic markers as *ASCL1*, *PHOX2B*, *TH*, and *SLC18A2*. Interestingly, we found a population sharing sympathetic and parasympathetic identities similar to previously published single-cell sequencing data in rodents showing cholinergic populations in stellate ganglia co-expressing *TH* and *DBH*^66^. Co-development of other spinal identities during progenitor caudalization may also account for sensory neurons developing in SNO. In either case, acetylcholine acting on pacemaker muscarinic receptors slightly decreased the beating rate. Notably, 8/27 iEHM constructs did not show a response, which likely reflects the low fraction of pacemaker-like cells in EHM (<10%) and/or insufficient axonal proximity required to elicit an HCN4-dependent effect.

Engineered cardiac tissues similar to EHM have been successfully employed to evaluate pro- and anti-arrhythmic compounds^67,68^. Since the iEHM enables direct assessment of the dynamic interplay between neuronal and cardiac modules, we reasoned that the model would allow us to identify the contribution of autonomic neuron dysregulation to a LQT2 arrhythmia phenotype. Our observations provide a mechanistic rationale for the clinical efficacy of cardiac sympathetic denervation in LQT2 patients to prevent arrhythmias and sudden cardiac death (SCD) in patients who do not respond to beta blocker treatment^69,70^. A clinical trial of 670 LQTS patients showed that, in contrast to LQT1 and LQT3, 43% of cardiac events in LQT2 patients are triggered by emotional stress^71^, further implicating sympathetic neuronal activity in arrhythmia susceptibility. However, it remains unclear whether autonomic neurons become hyperactive as a direct consequence of *KCNH2* mutations.

Exploiting the modular nature of the iEHM model, we explored the electrically excitable network dysregulation in the cardiac module (EHM), autonomic neuron module (SNO) and their combination (iEHM). Our cardiac tissues showed a delayed repolarization phenotype without additional functional or morphological deficits, consistent with prior iPSC-CM studies of diverse *KCNH2* variants^72–74^. Importantly, we could show that *KCNH2* mutants lead to hyperexcitability of peripheral autonomic organoids, underlining that mutations in *KCNH2* do not only affect the central nervous system (CNS) but also peripheral neurons, increasing the complexity of the disease along with the treatment regimen.

To delineate the autonomic contribution, we used a “mix & match” strategy combining mutant and isogenic cardiac/neural modules. To emulate central sympathetic drive, tissues were exposed to nicotine under muscarinic blockade (atropine). Double-mutant (_mut_EHM – _mut_SNO) iEHM displayed frequent afterdepolarizations (ADs), a canonical trigger to arrhythmia^75^. Across mutants, iEHM pro-arrhythmic phenotype severity correlated with intrinsic neuronal hyperexcitability. In contrast, mutant tissues challenged with isoprenaline—commonly used to mimic sympathetic input in the absence of neurons in cardiomyocyte monocultures—showed no pro-arrhythmic response. This data demonstrates that global β-adrenoreceptor stimulation may not simulate neuronal hyperactivity and may not be able to capture pathological pro-arrhythmic phenotype related to *KCNH2* mutations. Consistent with a possible non-adrenergic co-transmission, propranolol failed to rescue the pro-arrhythmic phenotype in iEHM, suggesting that afterdepolarizations may not be triggered by NA. One potential alternative trigger is neuropeptide Y, which is co-released with NA from SN and has been linked to arrhythmias^76–80^. Future studies should address whether neuropeptide Y inhibitors or other neuropeptide /neurotransmitter blockers represent a more effective antiarrhythmic treatment in patients carrying *KCNH2* mutations. Importantly, in contrast to propranolol, mexiletine, a sodium channel blocker suppressing excitability in both neurons and cardiomyocytes^48^, reduced afterdepolarizations in mutant iEHM. This observation aligns with clinical trials supporting mexiletine as a more effective therapy for cardiac *KCNH2*-related arrhythmias than β-blockers^81,82^. Notably, fusing p.R744del mutant SNOs to isogenic EHM still elicited afterdepolarizations—again prevented by mexiletine—underscoring a primary neuronal driver in arrhythmias which may be variant dependent^71–75^. Our data support a model in which arrhythmogenesis arises from coupled dysregulation of neuronal and cardiomyocyte systems. Accordingly, therapeutic strategies integrating autonomic modulation with direct targeting of cardiomyocyte electrophysiology may provide incremental benefit in selected arrhythmia subtypes, including *KCNH2*-related arrhythmias, where impaired repolarization reserve may confer heightened susceptibility to autonomically mediated triggers. These findings further suggest that mechanistically informed patient stratification—incorporating autonomic tone and genotype—may enable more precise deployment of combinatorial therapies in inherited and acquired arrhythmia syndromes.

In summary, iEHM is a neuro-cardiac interface 3D model integrating electrically active human autonomic neurons and CMs with supportive fibroblast, vascular, and immune components. Due to its modular design, it allows for combinatorial testing of the contribution of specific tissue types to brain-and-heart pathologies, such as SUDEP. The “mix & match” strategy in iEHM may be particularly useful in identifying cardiac liabilities due to neuronal dysfunction and delineating reciprocal effects of diseased neurons on healthy CMs and vice versa. This study establishes iEHM as a New Approach Methodology (NAM) that provides a human-relevant, physiologically integrated model for mechanistic investigations and pharmacological testing.

## Methods

### Pluripotent stem cell culture

The hiPSC lines (1) RUCDRi002-A, (2) optogenetic RUCDRi002-A-71, (3) UMGi093-A, (4) BIHi001-A-2 (5) MDCi052_A26 (R744del clone 1), (6) MDCi052_A27 (R744del clone 2), (7) MDCi052_A30 (G749A clone 1), and (8) MDCi052_A31 (G749A clone 2) were maintained in StemMACS™ iPS-Brew XF (Brew, Miltenyi, #130-104-368) containing 100 U/mL penicillin and 100 µg/mL streptomycin (Thermo Fisher, #15140-122). hiPSC were detached and singularized using EDTA solution (0.5 M EDTA/PBS) and replated on Matrigel (1:120, BD Bioscience, #354230) at a density of 8000–12,000 cells/cm^2^, passaged twice weekly in the presence of ROCK inhibitor (Y-27632, 5 μM, Stemgent, #04-0012).

Generation of the GMP line LiPSC-GR1.1 (also referred to as TC1133 or RUCDRi002-A; lot number 50-001-21) was supported by the NIH Common Fund Regenerative Medicine Program, and reported in Stem Cell Reports^83^. The NIH Common Fund and the National Center for Advancing Translational Sciences (NCATS) are joint stewards of the LiPSC-GR1.1 resource. Repairon GmbH acquired and imported a vial of the TC1133 master cell bank, from which a Working Cell Bank (WCB) was created. myriamed GmbH acquired a derivative of the WBC from Repairon GmbH and provided a non-GMP derivative thereof to the Institute of Pharmacology and Toxicology at the University Medical Center Göttingen for non-commercial research use. The BIHi001-A-2 iPSC-line was kindly provided by Sebastian Diecke, Max Delbrück Center, Berlin.

### Cardiomyocyte differentiation and purification

At a confluency of 80–90%, cells were submitted to cardiac differentiation as previously reported^17^. D0-d3, cells were supplemented daily with Mesoderm Induction Media (MIM: RPMI 1940 (Thermo Fisher, #61870-044), 2% B27 Supplement without insulin (Thermo Fisher, #A1895601), 200 µM L-ascorbic acid-2-phosphate sesquimagnesium salt hydrate (Sigma, #A8960) and 100 U/mL penicillin, 100 µg/mL streptomycin (Thermo Fisher, #15070063)) containing 9 ng/mL Activin A (R&D systems, #338-AC), 5 ng/mL BMP4 (R&D systems, #314-BP), 1 µM CHIR99021 (Stemgent, #04-0004), and 5 ng/mL bFGF (Miltenyi Biotech, #130-093-841). D3-d13, the cells were cultured in Basal Serum Free Medium (BSFM: RPMI 1940 (Thermo Fisher, #61870-044), 2% B27 Supplement (Thermo Fisher, #17504-044), 200 µM L-ascorbic acid-2-phosphate sesquimagnesium salt hydrate, and 100 U/mL penicillin, 100 µg/mL streptomycin) supplemented with 5 µM IWP4 (Stemgent, #04-0036) and d13-d17, cells were cultured in unsupplemented BSFM, which was changed every other day. For metabolic selection, CMs were cultured in RPMI without glucose and glutamine (Thermo Fisher, #11879020) containing 2.2 mM sodium lactate (Sigma, #71723), 100 µM β-mercaptoethanol (Invitrogen, #31350-010), and 100 U/mL penicillin, 100 µg/mL streptomycin) for 5 days.

### EHM generation

EHM were generated as previously described^17^. CM and human foreskin fibroblasts (HFFs) were combined at an 80:20 ratio and embedded in a pH-neutralized (pH 7.2–7.4) bovine collagen matrix (Collagen Solutions, #CS028; final concentration 1 mg/mL) prepared in RPMI 1640 (Thermo Fisher, #51800-035). A total of 450 µL of the cell–collagen suspension was cast into EHM tissue molds and allowed to polymerize for 1 h at 37 °C. Polymerized constructs were overlaid with basal EHM medium consisting of IMDM (Thermo Fisher, #31980-022), 4% B27 Supplement without Insulin (Thermo Fisher, #A1895601), 1% MEM Non-Essential Amino Acids (Thermo Fisher, #11140-035), 300 µM L-ascorbic acid-2-phosphate sesquimagnesium salt hydrate, 100 U/mL penicillin, 100 µg/mL streptomycin, 100 ng/mL IGF-1 (Peprotech, #AF-100-11), 10 ng/mL FGF-2 (Peprotech, #AF-100-18B), and 5 ng/mL VEGF-165 (Peprotech, #AF-100-20), supplemented with 5 ng/mL TGFβ1 (Peprotech, #AF-100-21C). For the first three days, EHM medium containing TGFβ1 was replenished daily. On day 4, constructs were transferred to flexible holders and subsequently maintained in basal EHM medium without TGFβ1, which was exchanged every other day until fusion with the SNO.

### BENO and SNO generation

BENO were generated as previously described^14^. SNO differentiation was based on the BENO protocol and was optimized by testing concentrations and treatment windows for morphogens BMP4, WNT and retinoic acid. Briefly, hiPSCs were dissociated using EDTA solution and resuspended in iPSC-Brew containing 10 μM ROCK inhibitor (Y-27632, Stemgent, #04-0012) and 20 ng/mL bFGF (Miltenyi Biotech, #130-093-841). A starting cell count of 2.5 × 10^5^ per organoid was used for SNO generation. The cell suspension was added to a pH-neutralized (pH 7.2–7.4) mixture of two-fold concentrated Dulbecco’s Modified Eagle’s Medium (Thermo Fischer, 12100-061) and bovine collagen (Collagen Solutions, #CS028, 50 µg per organoid). A casting volume of 50 µl per well was transferred into low-adhesion U-bottom 96-well plates (Sarstedt, #83.1837.500) and left to polymerize for 30 min at 37 °C. Then, the organoids were covered with 250 µl of iPSC-Brew containing 10 μM ROCK inhibitor and 10 ng/mL bFGF. In the following three days, SNO were supplemented with Basal Medium (Neurobasal A medium (Thermo Fisher, #10888-022), 2% B27 Supplement, 1% N2 -Supplement (Invitrogen, #17502-048), 200 µM L-ascorbic acid-2-phosphate sesquimagnesium salt hydrate, and 100 U/mL penicillin, 100 µg/mL streptomycin) containing 10 µM DAPT (Tocris, #2634), 100 nM LDN193189 (Sigma-Aldrich, #SML0559), 10 µM SB 431542 (Tocris, #1614), and 3 µM CHIR99021. D3, the organoids were transferred into 6-well plates (max. 8 organoids/well) and maintained with 5 mL of the previously described medium per well, which was replenished every other day. From this point until day five, SNO were additionally supplemented with 100 nM retinoic acid (Merck, #R4643) and 10 ng/mL BMP4 (R&D Systems, #314-BP). Then, SNO medium was changed to Basal Medium containing only 100 nM retinoic acid and 10 ng/mL BMP4. From d10-d15, SNO were cultured in Basal Medium containing 5 ng/mL TGFβ1 (Peprotech, #AF-100-21C), 10 ng/mL bFGF (Miltenyi- Biotech, #130-093-841) and 10 ng/mL BMP4. After this phase, the organoids were supplemented with Basal medium containing 5 ng/mL TGFβ1 and 2.5 µM DAPT for neuronal differentiation and maturation. From day 28 forward, SNO were sustained with Basal Medium until further analysis. Between d3 and d30, the tissues compact as a result of traction forces generated by expanding cell populations^84^.

### iEHM generation

In week 4 of maturation, EHM were fused with d40 SNO by placing the SNO in the center of the muscle ring. From this point forward, the iEHM were supplemented with a 1:1 mixture of Basal medium and EHM medium. After 3-5 days, SNO started adhering to the EHM, and after two weeks, the tissues fully fused at their points of contact. The iEHMs were cultured in this medium for 6-8 weeks prior to analysis.

### Immunofluorescent staining of 2D cells

Cells on glass coverslips were fixed by incubating with 4% formaldehyde solution (Histofix, Carlroth) for 15 min at RT. The formaldehyde was removed, and the cells were washed three times with PBS. Prior to staining, the cells were permeabilized using Buffer A (PBS, 3 g/L Triton-X-100, 2 g/L BSA) for 10 min at RT and unspecific binding was blocked using Buffer B (PBS, 1 g/L Triton-X-100, 5 g/L BSA) for one hour at RT. Subsequently, the primary antibodies were applied in Buffer C (PBS, 1 g/L Triton-X-100, 1 g/L BSA) at 4 °C overnight. Next, the cells were washed 3 times with PBS and incubated with the secondary antibodies in Buffer C for one hour at RT. This and all the following steps were performed in the dark. After this, the coverslips were washed three times using PBS, after which nuclear staining was applied (1 μg/ml Hoechst 33342 (Sigma) in PBS) for 10 min at RT. Finally, the cells were washed three times with PBS, followed by one washing step using water. Coverslips were inverted and mounted on glass slides using Mowiol mounting medium (10% Fluoromount Mowiol 4–88 (Carlroth, #0713.1), 25% Glycerol (Merck-Millipore, #56-81-5), 0.1 M Tris (ITW Reagents, #A1086), 0.2% n-propyl gallate (Sigma-Aldrich, #P3130)). An antibody list with respective dilutions is provided in Supplementary Table 2.

### Whole-mount immunofluorescence staining (WmIF)

Tissues were fixed with 4% formaldehyde solution (Histofix, Carl Roth) for 2 h at 4 °C. Subsequently, they were washed twice with PBS and blocked for 30 min at 4 °C with staining buffer (StB; 5% FBS, 1% BSA, 0.5% Triton X-100 in PBS). Tissues were incubated with primary antibodies diluted in StB for 2 days at 4 °C (100 µl/tissue). Upon washing with StB for 6–8 h, tissues were incubated with secondary antibodies and Hoechst 33342 (Sigma) for another 2 days at 4 °C. After StB washings for a total of 6–8 h, BENOs were mounted on glass coverslips with Mowiol mounting medium (10% Fluoromount Mowiol 4-88 (Carlroth, #0713.1), 25% Glycerol (Merck-Millipore, #56-81-5), 0.1 M Tris (ITW Reagents, #A1086), 0.2% n-propyl gallate (Sigma-Aldrich, #P3130)). WmIF was visualized using confocal imaging performed on a Zeiss LSM 710 confocal microscope equipped with 10x and 63x objectives with ZEN 2010 software and further analyzed by ZEN and ImageJ. Nonlinear adjustment by modest gamma stretching in ZEN 2010 was used in images to reduce the background in tissues. Surface and volume rendering of images were used to generate Supplementary Movies 2–7. An antibody list with respective dilutions is provided in Supplementary Table 2.

### Orientation analysis of axons and vessels in iEHM

A convolutional neural network (U-Net^85^) to detect binary masks of vasculature (marked by PECAM1) and axons (labeled by NF) from confocal microscopy images was used. For this analysis, each of the 6–10 images from random regions of three iEHM was analyzed (25 images total). For both vasculature and axons, neural network models were trained with expert-annotated images of a separate data set (using a combined BCE-Dice loss, 50 epochs, learning rate 1e-3). The predicted binary masks of vasculature and axons were skeletonized, and a progressive probabilistic line Hough transform^86^ for spatially resolved orientation analysis was applied. Then, the distribution of orientation angles for both vasculature and axons was analyzed. To quantify their correlation, the Pearson correlation coefficient R based on the histogram counts was calculated. Code is available in the Code availability section.

### Tissue digestion and flow cytometry

For SNO and BENO digestion, d15 organoids were sequentially incubated for one hour with collagenase solution (PBS +Ca/Mg, 0.25% Collagenase Type I (Sigma-Aldrich, #C0130), 20% FBS (Thermo Fisher, #A4766801)) and Accutase dissociation reagent (97% StemPro® Accutase® Cell Dissociation Reagent (Merck-Millipore, #SCR005), 0.025% Trypsin (Thermo Fisher, #15090-046), 20 μg/mL DNaseI (Calbiochem, #260913). The digested cells were fixed in 4% formaldehyde at room temperature (RT) for 15 min and subsequently labeled with anti-PHOX2B primary antibody (Santa Cruz, #sc-376997, 1:250) and Hoechst 33342 in StB for 45 min at 4 °C. To remove the primary antibody, the cells were centrifuged at 3000 x *g*, the supernatant was discarded, and the cells were resuspended and incubated in StB for five minutes at 4 °C. This step was repeated three times. Subsequently, cells were incubated with secondary antibody Alexa Fluor 488 (Invitrogen, #A32723, 1:1000) for 30 min at 4 °C under the exclusion of light. Following another 3 × 5 min washing step, the cells were analyzed using a BD LSR II flow cytometer (BD Biosciences) and FACSDiva (BD Biosciences) as well as FLOWJO software.

### Liquid chromatography-coupled tandem mass spectrometry (LC-MS/MS)

SNO were lysed in 500 µl precipitating agents (80% acetonitrile and 20% water), including noradrenaline-d6, choline-d9 and buformin as internal standards for NA, acetylcholine, and dopamine, respectively. Following 15 min incubation at RT, 400 µL of the liquid phase was evaporated under a nitrogen stream. The residues were reconstituted in 400 µl of 0.1% (v/v) formic acid, and 10 µl were injected into the LC-MS/MS system. NA, dopamine, and acetylcholine (ACh) content of SNO were analyzed by high-performance liquid chromatography (HPLC) − MS/MS using a Shimadzu Nexera HPLC system with a LC-30AD pump, a SIL-30AC autosampler, a CTO-20AC column oven, and a CBM20A controller (Shimadzu, Kyoto, Japan). Separation was achieved on an Imtakt-Intrada Amino Acids Separation Column, 100 × 3.0 mm (ChromTech-, #WAA34) with a C-18 guard pre-column. The HPLC was run at a low speed of 600 µL/min with an oven temperature of 37 °C. The mobile phase consisted of 29.85% 200 mM ammonium formate, 0.15% (v/v) formic acid and 70% (v/v) methanol. The HPLC system was coupled to an API 4000 tandem mass spectrometer (SCIEX, Darmstadt, Germany). Buformin (internal standard), NA-d6 (internal standard), and choline-d9 (internal standard) were quantified using the detection parameters given (Supplementary Table 3). Analysis was performed using the Analyst software (version 1.6.2, SCIEX, Darmstadt, Germany) and standard curves obtained by weighted linear regression. Representative spectra and standard curves are provided in Supplementary Fig. 5.

### Quantitative real-time (qRT) PCR

Total RNA was extracted using the NucleoSpin RNA Mini kit (Macherey Nagel, #740955). 250 ng to 1 µg of RNA were reverse transcribed using M-MLV Reverse transcriptase (Promega, #M1705) and oligo(dT) primer. qRT-PCR was performed using Takyon Blue dTTP Master Mix (Eurogentec, #UF-NSMT-B0701). The primer sequences were designed using NCBI Primer Blast and span an exon-exon junction. The primer list is provided in Supplementary Table 4. Primers were ordered from Microsynth AG (Göttingen, Germany). Analysis was performed by the 7900HT Real Time PCR System (Applied Biosystems) and the Sequence Detection System (SDS) v2.4 software (Applied Biosystems). Transcript expression was normalized to *GAPDH* levels. When different cardiomyocyte-specific transcripts were evaluated, *TNNT2* was used for normalization to exclude variability due to differentiation efficiency between cell lines in Supplementary Fig. 19.

### Calcium imaging

The iEHM were stained with 1 µg/ml Fluo-8-AM (Abcam, #ab142773) in carbogenated artificial cerebrospinal fluid (ACSF) buffer (26 mM NaHCO_3_, 10 mM Glucose, 1 mM MgSO_4_·7H_2_O, 1.25 mM NaH_2_PO_4_, 2.5 mM KCl, 126 mM NaCl, 2 mM CaCl_2_) at pH 7.3 for 15-30 min. During calcium imaging, iEHM were continuously perfused with ACSF at 37 °C. Fluorescence was recorded at 2–5 Hz, and measurements were performed using a Zeiss LSM 780 confocal microscope and ZEN 2010 software. The stimulation of ACh to muscarinic receptors was blocked by the addition of 100 nM atropine (Sigma Aldrich, #A0257). In the presence of atropine, 30 µM nicotine (Simga Aldrich, #N1019) was used to stimulate nicotinic receptors on autonomic neurons. β-adrenoreceptor blockade was achieved by adding 1 µM propranolol (Sigma Aldrich, #P0884). Analysis was performed using MATLAB R2012a (The MathWorks, USA).

### Force of contraction analysis

Isometric force measurements of EHM / iEHM were performed in an organ bath setup as described previously^17^. EHM and iEHM were mounted on two opposing hooks and submerged in normal Tyrode’s solution (120 mM NaCl, 5.36 mM KCl, 0.2 mM CaCl_2_, 10.5 mM MgCl_2_, 22.61 mM NaHCO_3_, 4.2 mM NaH_2_PO_4_, 5.55 mM Glucose, 0.57 mM L-Ascorbic Acid) at 37 °C with a continuous flow of carbogen from beneath. Force changes were measured by a linear isometric force transducer mounted to the upper hook. The basal tension of the tissues was adjusted to 0.1 mN using a micromanipulator. On either side, a field electrode was placed for electrical pacing of the tissues.

### Voltage imaging of 2D CMs

CMs were dissociated using Accutase, replated on Matrigel-coated (1:120) glass cover slips at 1.042 × 10^4^ cells/cm^2^, and cultured for 5–7 days to normalize. The coverslips were washed twice with Tyrode’s solution (120 mM NaCl, 5.36 mM KCl, 2.0 mM CaCl_2_, 10.5 mM MgCl_2_, 22.61 mM NaHCO_3_, 4.2 mM NaH_2_PO_4_, 5.55 mM Glucose, 0.57 mM L-Ascorbic Acid). After that, the cells were stained using the FluoVolt™ Membrane dye and PowerLoad™ concentrate (Invitrogen, F10488) diluted in Tyrode’s solution according to the manufacturer’s instructions at RT for 15 min. After that, the cells were washed twice and then covered with Tyrode’s solution. Imaging was performed using the Zeiss LSM 710 confocal microscope with a 63x oil objective and the ZEN 2010 software at 37 °C under external pacing at 0.5 Hz (18 mA, 5 ms pulse duration). Voltage profiles were analyzed using the line-scan mode (512 pixels, 45 μm, 1057.7 Hz, 20,000 cycles). The traces were extracted using ImageJ and smoothed using the Savitzky-Golay polynomial smoothing algorithm (quartic filter, length: 101). APs were interpreted using LabChart (ADInstruments) and the Peak Analysis Tool.

### Electrical field potential measurements by multielectrode-array (MEA)

SNO were embedded in 3% low melting agarose and sectioned using Leica VT1000S vibratome (Leica) into slices of 400 µm diameter. SNO slices were mounted on Lumos MEA 48-well plates (Axion BioSystems, #M768-tMEA-480PT) coated with 1:30 Matrigel. Gold grids for transmission electron microscopy (Gilder, #G50HEX-G3) were used to stabilize the tissue. The measurements of electrical field potentials were performed by Maestro Pro (Axion BioSystems) at 37 °C and 5% CO_2_. The Lumos system (Axion BioSystems) enabled optogenetic stimulation of the organoids by delivering a pentaphasic light pulse with a central wavelength of 612 nm and an intensity of 1.54 mW/mm^2^ every two minutes for one hour. Stimulation experiments were performed by the addition of increasing concentrations (1 µM-1 mM) of nicotine diluted in pre-warmed Basal medium, while the added volume never exceeded five percent of the well’s media volume. Analysis was performed by the Neural Metrics and Plotting Tool software (Axion BioSystems).

### Single-nuclei RNA sequencing

Nuclei were isolated from snap frozen iEHM by a protocol adapted from Sakib et al^87^. In brief, 500 µL EZ prep nuclei lysis buffer (Sigma, #NUC101) was added to the frozen samples, and dounce homogenized for 40–60 strokes (until no big tissue chunks were seen) using a plastic pestle in a 1.5 mL DNA low-binding tube. Lysates were transferred into a 2 mL DNA low-binding tube and diluted to 2 mL by the addition of lysis buffer. Samples were incubated for 5 min on ice and centrifuged at 500 x *g* for 5 min at 4 °C. Supernatants were discarded, the nuclei pellets were resuspended in 2 mL lysis buffer, and incubated on ice for 5 min. After centrifugation (500 x *g*, 5 min, 4 °C), the pellet was resuspended in Nuclei Suspension buffer (NSB). NSB was prepared by the addition of 0.5% OmniPur BSA (Merck Millipore, #2905), 1:200 RNasin® Ribonuclease Inhibitor (Promega, #N2615), 1x cOmplete™, EDTA-free Protease Inhibitor Cocktail (Roche, #04693132001) in 1x PBS (RNAse free, Invitrogen, #AM9624) and filtered through 0.22 µm Syringe Filter Unit (Millipore, #SLGVR33RB). Upon resuspension, the sample was centrifuged at 500 x *g*, 5 min at 4 °C, and resuspended in 500 µL NSB. The lysate was strained through 40 µM Flowmi® Cell Strainers (Sigma, #BAH136800040) into a FACS tube. To discriminate nuclei from debris, 7-AAD (Invitrogen, #00-6993-50) nuclei staining solution (1:500) was added to the sample (Supplementary Fig. 8), which was sorted by a BD FACSaria III with 85 µM Nozzle, into a 15 mL Falcon tube, coated with and containing 1 mL NSB. Around 200,000 nuclei were sorted per sample. Sorted iEHM nuclei were immediately processed for single nuclei barcoding using 10X Genomics Chromium Single Cell 3’ Reagent Kits (v3 Chemistry). 5000 nuclei were subjected to GEM generation and barcoding using Chromium Controller, according to the manufacturer’s protocol. For cDNA amplification, 14 PCR cycles were used. Indexed cDNA libraries were pooled into a single pool and sequenced 4 times in Illumina NextSeq 550 to achieve >50,000 reads/nuclei. Raw BCL files were demultiplexed, mapped, and count files were generated using cellranger-4.0.0, using hg38 pre-mRNA reference genome.

### Single-nuclei RNA-sequencing data analysis

Cell Ranger (10x Genomics) was used for demultiplexing, alignment and generation of gene counts. The dataset was filtered to remove nuclei where less than 200 genes were detected, as well as genes that were expressed in less than 10 nuclei. Any nuclei expressing more than 1% mitochondrial gene counts were also removed. The data were normalized using the sctransform^88^ package (version 0.3.2) from the Seurat^89^ (version 4.0.2) toolkit in R (version 4.0.1). DoubletFinder^90^ (version 2.0.3) was used to detect and remove any potential doublets. Leiden clustering^91^ was performed using the FindClusters() function in Seurat, and Uniform Manifold Approximation and Projection (UMAP)^92^ was used for visualization of clusters. The top markers for each cluster were detected using the FindAllMarkers() function in Seurat. Cell-type annotation of clusters was done using the expression of selected marker genes as listed in Supplementary Fig. 9. The same analysis pipeline was followed for separate analyses of specific cell clusters after subsetting the original dataset accordingly.

### RNA extraction, library preparation, and bulk RNA-sequencing

RNA from iEHM and EHM/SNO was harvested using the Promega ReliaPrep RNA Tissue Miniprep system and was cleaned with the RNA Clean & Concentrator. 500 ng of total RNA was used for the construction of sequencing libraries. RNA quality was assessed by measuring the RNA Integrity Number (RIN) using the Fragment Analyzer HS Total RNA Kit (DNF-472-FR; Agilent Technologies). Library preparation was carried out using the STAR Hamilton NGS automation platform, applying the Illumina Stranded mRNA Prep Kit (Cat. No. 20040534) and the IDT for Illumina RNA UD Indexes Set A, Ligation with 96 Indexes (Cat. No. 20091646), starting from 200 ng of total RNA. The size range of the final cDNA libraries was determined using the SS NGS Fragment 1–6000 bp Kit on the Fragment Analyzer, yielding an average fragment size of 340 bp. Accurate library quantification was performed with the DeNovix DS-Series System. Libraries were sequenced on the NovaSeq 6000 using an S2 flow cell (100 cycles), producing approximately 20–25 million reads per sample.

### Read alignment and quantification

High-quality reads were aligned to the *Homo sapiens* reference genome (GRCh38.p13, Ensembl) using the STAR aligner^93^. Gene-level quantification was performed with FeatureCounts (Subread v1.6.3). The resulting count matrices were imported into the R/Bioconductor environment for differential expression analysis.

### Normalization and differential expression analysis

Gene-level counts were analyzed using DESeq2 in R (v4.3.2)^94^. Genes corresponding to mitochondrial (MT-) and ribosomal (RPL-, RPS-) transcripts were removed prior to normalization, and genes with fewer than two total counts across all samples were filtered out. Samples were assigned to two experimental groups (SNO + EHM and iEHM), and outliers identified by quality assessment were excluded. Differential expression between groups was modeled with a negative binomial generalized linear model. Shrinkage of log_2_ fold changes was applied using the apeglm algorithm^95^. Genes were classified as significantly differentially expressed if they showed an adjusted *P* value < 0.05 (Benjamini–Hochberg correction) and an absolute log_2_ fold change > 1^90^. For Supplementary Fig. 17, selected expression data were normalized to a cardiomyocyte-enriched reference gene to reflect changes within the cardiomyocyte compartment rather than across the total mixed-tissue sample. In addition to *CASQ2*, corresponding data normalized to a conventional housekeeping gene found to be stably expressed among iPSC-derived cardiomyocytes (*DDB1*)^96^ are also shown.

### Data visualization and functional interpretation

All analyses were performed in R using the following core packages: DESeq2, ggplot2, ggrepel, and ComplexHeatmap. Volcano plots were generated with ggplot2^97^ and ggrepel to visualize log₂ fold changes against –log₁₀ adjusted *P* values. Thresholds were indicated at |log_2_ FC| = 1 and adjusted *P* = 0.05, and the top up- and downregulated genes were annotated. Normalized counts were transformed using the variance-stabilizing transformation (VST) in DESeq2 to obtain homoscedastic expression values suitable for clustering and visualization. To explore gene expression patterns within biologically relevant pathways, selected genes were grouped into four functional categories—metabolic, hypertrophy-associated, immune, and cardiac—based on literature evidence. Heatmaps were generated using ComplexHeatmap^98^ with hierarchical clustering applied to both genes and samples. Annotation bars denoted experimental groups and functional categories, and auxiliary panels represented log_2_ fold change and –log_10_ FDR values.

### Clustering and functional enrichment analyses

Variance-stabilized gene expression values obtained from DESeq2 were subjected to unsupervised clustering to identify coordinated expression modules. The top 500 most variable genes were selected based on row variance, scaled (Z-score), and reduced via principal component analysis (PCA). Genes were then grouped into three expression clusters (*k* = 3) using k-means clustering, defining transcriptional modules with shared variance structure across iEHM and SNO + EHM samples. Each gene cluster was subjected to Gene Ontology (GO) enrichment analysis using the clusterProfiler package^99^ with the *org.Hs.eg.db* annotation database. Both Biological Process (BP) and Cellular Component (CC) ontologies were tested using the Benjamini–Hochberg correction (*P* < 0.05, *q* < 0.2). The most enriched terms per cluster were visualized using bar and dot plots, highlighting distinct functional signatures across expression modules. To further contextualize functional patterns, Reactome pathway enrichment was performed with the Reactome PA package^100^. Pathways with adjusted *P* values < 0.05 were retained, and results were visualized via dot plots and cnet plots to display gene–pathway relationships. For network-level visualization, protein–protein interaction (PPI) networks were reconstructed for each expression cluster using the STRING database (v11.5)^101^. Gene symbols were mapped to STRING identifiers, and interaction networks were exported as high-resolution vector graphics, enabling visualization of key molecular hubs and connectivity patterns within each transcriptional module.

### Statistical analysis

Statistical testing was performed in GraphPad Prism 9. All values are displayed as mean ± s.e.m. Statistical tests were chosen depending on the parametricity of the sample distribution, which was determined by the Shapiro-Wilk and *F*-test. The respective tests as well as sample size (*n*) and number of independent experiments (*N*) are mentioned in the figure legends. Statistically significant differences were assumed with a probability value of *P* ≤ 0.05. Exact *P*-values are given in the figure.

### Reporting summary

Further information on research design is available in the Nature Portfolio Reporting Summary linked to this article.

## Supplementary information

## Source data

## Data Availability

The single-nucleus RNA sequencing data generated in this study have been deposited in the NCBI Gene Expression Omnibus database under accession code GSE313106. The bulk RNA sequencing data generated in this study have been deposited in the EBI biostudies database under accession code E-MTAB-16232. Source data are provided with this paper.

## Code availability

Custom Python scripts for deep learning and image analysis were used. The U-Net implementation is publicly available at https://github.com/danihae/bio-image-unet. The full analysis pipeline, including the iEHM-specific model and processing scripts, can be accessed at https://github.com/danihae/iEHM-analysis. The dataset used in this study is archived at Zenodo (https://zenodo.org/records/21268390) for reproducibility and further analysis.
