# Supplementary material for: Innervated human cardiac muscle model reveals sympathetic drivers of KCNH2-associated arrhythmias
Source: Nat Commun. 2026 Sep 2;17:9404. doi: 10.1038/s41467-026-76956-9 (PMC13538559; doi:10.1038/s41467-026-76956-9)
Supplement: Supplementary file 1 [file 41467_2026_76956_MOESM15_ESM.pdf]

nature portfolio

Corresponding author(s): Maria Patapia Zafeiriou

Last updated by author(s): Jul 23, 2026

Reporting Summary

Nature Portfolio wishes to improve the reproducibility of the work that we publish. This form provides structure for consistency and transparency in reporting. For further information on Nature Portfolio policies, see our Editorial Policies and the Editorial Policy Checklist.

Statistics

For all statistical analyses, confirm that the following items are present in the figure legend, table legend, main text, or Methods section.

n/a	Confirmed	
☐	☒	The exact sample size (n) for each experimental group/condition, given as a discrete number and unit of measurement
☐	☒	A statement on whether measurements were taken from distinct samples or whether the same sample was measured repeatedly
☐	☒	The statistical test(s) used AND whether they are one- or two-sided<br>*Only common tests should be described solely by name; describe more complex techniques in the Methods section.*
☐	☒	A description of all covariates tested
☐	☒	A description of any assumptions or corrections, such as tests of normality and adjustment for multiple comparisons
☐	☒	A full description of the statistical parameters including central tendency (e.g. means) or other basic estimates (e.g. regression coefficient) AND variation (e.g. standard deviation) or associated estimates of uncertainty (e.g. confidence intervals)
☐	☒	For null hypothesis testing, the test statistic (e.g. F, t, r) with confidence intervals, effect sizes, degrees of freedom and P value noted<br>*Give P values as exact values whenever suitable.*
☒	☐	For Bayesian analysis, information on the choice of priors and Markov chain Monte Carlo settings
☒	☐	For hierarchical and complex designs, identification of the appropriate level for tests and full reporting of outcomes
☐	☒	Estimates of effect sizes (e.g. Cohen's d, Pearson's r), indicating how they were calculated

Our web collection on statistics for biologists contains articles on many of the points above.

Software and code

Policy information about availability of computer code

Data collection	Zeiss LSM 710<br>ZEN 2010<br>BD LSR II flow cytometer and FACSDiva<br>API 4000 tandem mass spectrometer<br>7900HT Real Time PCR System<br>Axion BioSystems Maestro Pro<br>Illumina NextSeq 550<br>NovaSeq 6000
Data analysis	FLOWJO software<br>ImageJ<br>Analyst software (v1.6.2)<br>Sequence Detection System software (v2.4)<br>Matlab R2012a<br>Axion BioSystems Neural Metrics and Plotting Tool software<br>GraphPad Prism 9<br>10x Geneomics Cell Ranger<br>Seurat (version 4.0.2) toolkit in R (version 4.0.1)<br>DoubletFinder (version 2.0.3)

STAR aligner
FeatureCounts (Subread v1.6.3)
DESeq2 in R (v4.3.2)

For manuscripts utilizing custom algorithms or software that are central to the research but not yet described in published literature, software must be made available to editors and reviewers. We strongly encourage code deposition in a community repository (e.g. GitHub). See the Nature Portfolio guidelines for submitting code & software for further information.

Data

Policy information about availability of data

All manuscripts must include a data availability statement. This statement should provide the following information, where applicable:

- Accession codes, unique identifiers, or web links for publicly available datasets
- A description of any restrictions on data availability
- For clinical datasets or third party data, please ensure that the statement adheres to our policy

Single nuclei RNA sequencing data in this study have been deposited in Gene Expression Omnibus under accession code GSE313106.
Bulk RNA sequencing data in this study have been deposited in EBI biostudies https://www.ebi.ac.uk/biostudies/arrayexpress/studies/E-MTAB-16232.

Research involving human participants, their data, or biological material

Policy information about studies with human participants or human data. See also policy information about sex, gender (identity/presentation), and sexual orientation and race, ethnicity and racism.

Reporting on sex and gender	No studies involving human research participants
Reporting on race, ethnicity, or other socially relevant groupings	Not applicable
Population characteristics	Not applicable
Recruitment	Not applicable
Ethics oversight	Not applicable

Note that full information on the approval of the study protocol must also be provided in the manuscript.

Field-specific reporting

Please select the one below that is the best fit for your research. If you are not sure, read the appropriate sections before making your selection.

☒ Life sciences ☐ Behavioural & social sciences ☐ Ecological, evolutionary & environmental sciences

For a reference copy of the document with all sections, see nature.com/documents/nr-reporting-summary-flat.pdf

Life sciences study design

All studies must disclose on these points even when the disclosure is negative.

Sample size	No prior sample size calculation was performed. For all experiments containing statistical analysis, a samples size of at least n=3 was chosen.
Data exclusions	Quantification of EHM and iEHM beating rate: Excluded 8 out of 27 iEHM that was not responding, more details in Supplementary Figure 15a.<br>Single nuclei RNA sequencing: Removed nuclei where less than 200 genes were detected & genes that were expressed in less than 10 nuclei. Nuclei expression more than 1% mitochondrial gene were also removed.<br>Bulk RNA sequencing: Mitochondrial and ribosomal transcripts were removed. Genes with fewer than 2 total counts across all samples were filtered out.
Replication	Only iPSCs with pluripotency over 90% for TRA1-60, OCT3/4 and NANOG was used for SNO generation. SNOs were evaluated at day 15 and day 30 by wholemount immunofluorescence to assess for presence of autonomic progenitor marker (PHOX2B) and sympathetic marker (TH and DBH). 3 independent differentiations of SNO were performed and with 3 different cell lines to show reproducibility of the protocol.<br>At least 2 independent differentiation of iEHM were performed. Sample number and independent differentiations are mentioned in the Figure legend.
Randomization	Randomization was not relevant in this study. Two different groups were analyzed (BENO vs SNO, EHM vs iEHM). In the KCNH2 disease modeling, three different groups were analyzed namely R744del and G749A mutation and control. For all groups, all successfully formed tissues were used for further analysis.
Blinding	Blinding was not relevant in this study. Two different groups were analyzed (BENO vs SNO, EHM vs iEHM). In the KCNH2 disease modeling, three different groups were analyzed namely R744del and G749A mutation and control. For all groups, all successfully formed tissues were used for further analysis.

Behavioural & social sciences study design

All studies must disclose on these points even when the disclosure is negative.

Study description	Not applicable
Research sample	Not applicable
Sampling strategy	Not applicable
Data collection	Not applicable
Timing	Not applicable
Data exclusions	Not applicable
Non-participation	Not applicable
Randomization	Not applicable

Ecological, evolutionary & environmental sciences study design

All studies must disclose on these points even when the disclosure is negative.

Study description	Not applicable
Research sample	Not applicable
Sampling strategy	Not applicable
Data collection	Not applicable
Timing and spatial scale	Not applicable
Data exclusions	Not applicable
Reproducibility	Not applicable
Randomization	Not applicable
Blinding	Not applicable

Did the study involve field work? ☐ Yes ☒ No

Reporting for specific materials, systems and methods

We require information from authors about some types of materials, experimental systems and methods used in many studies. Here, indicate whether each material, system or method listed is relevant to your study. If you are not sure if a list item applies to your research, read the appropriate section before selecting a response.

Materials & experimental systems

n/a	Involved in the study	
☐	☒	Antibodies
☐	☒	Eukaryotic cell lines
☒	☐	Palaeontology and archaeology
☒	☐	Animals and other organisms
☒	☐	Clinical data
☒	☐	Dual use research of concern
☒	☐	Plants

Methods

n/a	Involved in the study	
☒	☐	ChIP-seq
☐	☒	Flow cytometry
☒	☐	MRI-based neuroimaging

Antibodies

Antibodies used	anti-ACTN2, Sigma, A7811<br>anti-BRN3A, Merck, MAB1585

	anti-CTNT, Abcam, ab45392<br>anti-CD45, Abcam, Ab8216<br>anti-CD68, Synaptic Systems, HS-460-008<br>anti-DBH, Sigma, HPA070789<br>anti-HCN4, Abcam, ab69054<br>anti-Neurexin 1, Sigma, HPA071400<br>anti-NF (H), Synaptic Systems, 171 106<br>anti-NF (H), Biolegend, 822601<br>anti-PAX6, Biolegend, 901301<br>anti-PDGFRb, Cell Signalling, 28E1<br>anti-PECAM1, DAKO, M0823<br>anti-Peripherin, NOVUS, NBP1-05423<br>anti-Phalloidin, Invitrogen, A12379<br>anti-PHOX2B, Santa Cruz, sc-376997<br>anti-Synapsin 1, Synaptic Systems, 106 011<br>anti-TH, Synaptic Systems, 213 004<br>anti-VAChT, Synaptic Systems, 139 103<br>anti-VGLUT1, NanoTag, N1602-Abb635P<br>anti-chicken Alexa Fluor 488, Invitrogen, A10039<br>anti-rabbit Alexa Fluor 546, Invitrogen, A11035<br>anti-mouse Alexa Fluor 546, Invitrogen, A11074<br>anti-guinea pig Alexa Fluor 633, Invitrogen, A21052
Validation	All antibodies used were validated by the respective manufacturer. Antibody specificity were assessed with a positive and negative control.

Eukaryotic cell lines

Policy information about cell lines and Sex and Gender in Research

Cell line source(s)	Human induced pluripotent stem cell lines:<br>RUCDRi002-A: Male, obtained from Institute of Pharmacology and Toxicology, University Medical Center Goettingen.<br>RUCDRi002-A-71: Male, obtained from Institute of Pharmacology and Toxicology, University Medical Center Goettingen.<br>UMGi093-A: Male, obtained from Lukas Cyganek (Stem Cell Unit, University Medical Center Goettingen).<br>BIHi001-A-2: Male, obtained from Sebastian Diecke (Max Delbrück Center, Berlin).<br>MDCi052_A26: Male, generated by Sebastian Diecke (Max Delbrück Center, Berlin).<br>MDCi052_A27: Male, generated by Sebastian Diecke (Max Delbrück Center, Berlin).<br>MDCi052_A30: Male, generated by Sebastian Diecke (Max Delbrück Center, Berlin).<br>MDCi052_A31: Male, generated by Sebastian Diecke (Max Delbrück Center, Berlin).
Authentication	Cell lines that are well-established:<br>RUCDRi002-A: https://hpscreg.eu/cell-line/RUCDRi002-A<br>RUCDRi002-A-71: https://www.sciencedirect.com/science/article/pii/S1873506124000151?via%3Dihub<br>UMGi093-A: https://sfb1002.med.uni-goettingen.de/production/cellmodel/cell-line/view?tab=internal&line=125<br>BIHi001-A-2: https://www.cellosaurus.org/CVCL_C0NJ<br><br>Cell lines established in the paper:<br>MDCi052_A26: Sub-clone of RUCDRi002-A, mutation was validated by PCR and Sanger Sequencing. Pluripotency analysis and 3 germ layer differentiation were performed.<br>MDCi052_A27: Sub-clone of RUCDRi002-A, mutation was validated by PCR and Sanger Sequencing. Pluripotency analysis and 3 germ layer differentiation were performed.<br>MDCi052_A30: Sub-clone of RUCDRi002-A, mutation was validated by PCR and Sanger Sequencing. Pluripotency analysis and 3 germ layer differentiation were performed.<br>MDCi052_A31: Sub-clone of RUCDRi002-A, mutation was validated by PCR and Sanger Sequencing. Pluripotency analysis and 3 germ layer differentiation were performed.
Mycoplasma contamination	All cell lines were routinely checked for mycoplasma contaminations and were tested negative.
Commonly misidentified lines<br>(See ICLAC register)	No misidentified lines have been used in this study.

Plants

Seed stocks	Not applicable
Novel plant genotypes	Not applicable
Authentication	Not applicable

Flow Cytometry

Plots

Confirm that:

- [x] The axis labels state the marker and fluorochrome used (e.g. CD4-FITC).
- [x] The axis scales are clearly visible. Include numbers along axes only for bottom left plot of group (a 'group' is an analysis of identical markers).
- [x] All plots are contour plots with outliers or pseudocolor plots.
- [x] A numerical value for number of cells or percentage (with statistics) is provided.

Methodology

Sample preparation	SNOs were dissociated into single cells using Collagenase Type I and Accutase dissociation reagent. Digested cells were fixed in 4% formaldehyde at room temperature for 15 min and stained for FACS.<br>iEHMs were lysed with EZ prep nuclei lysis buffer and the pellet was resuspended in Nuclei Suspension buffer. Lysate was strained through 40 uM Flowmi Cell Strainers into FACS tube and stained.<br>Cardiomyocytes were dissociated into single cells using Accutase dissociation reagent. Digested cells were fixed in cold 70% ethanol. Cells were subsequently stained for FACS.
Instrument	Cells were measured with BD LSR II Flow cytometer or BD FACSaria III.
Software	Data were analyzed with FACSDiva and FLOWJO.
Cell population abundance	Abundance of cell population is determined as a % of the parent population gating (gated after FSC/SSC plot and Hoechst-A/SSC-A). Isotype control were used to distinguish between the negative and positive signals.
Gating strategy	Cells were gated by the FSC/SSC plot and further gated by Hoechst-A/SSC-A or 7-AAD/SSC-A signal. Isotype control were used to distinguish between negative and positive signals.

- [x] Tick this box to confirm that a figure exemplifying the gating strategy is provided in the Supplementary Information.
