# Supplementary material for: Innervated human cardiac muscle model reveals sympathetic drivers of KCNH2-associated arrhythmias
Source: Nat Commun. 2026 Sep 2;17:9404. doi: 10.1038/s41467-026-76956-9 (PMC13538559; doi:10.1038/s41467-026-76956-9)
Supplement: Supplementary file 2 [file 41467_2026_76956_MOESM16_ESM.pdf]

nature portfolio

Peer Review File

Innervated human cardiac muscle model reveals sympathetic drivers of KCNH2-associated arrhythmias

Corresponding Author: Dr Maria Patapia Zafeiriou

This manuscript has been previously reviewed at another journal. This document only contains information relating to versions considered at Nature Communications.

This file contains all reviewer reports in order by version, followed by all author rebuttals in order by version.

Version 1:

Reviewer comments:

Reviewer #1

(Remarks to the Author)
I appreciate the authors clarifications to the points raised in my previous assessment.

The newly provided single-cell data indicate that the neuronal compartment of iEHM is heterogeneous, with noradrenergic neurons representing only a minority and a substantial fraction of cholinergic or mixed-identity neurons. This raises an important question about how the reported high noradrenaline and low acetylcholine levels should be interpreted, and whether the disease phenotype in KCNH2 mutant tissues can be attributed specifically to sympathetic neurons.

The pharmacological data are consistent with an autonomic contribution and may reflect partly sympathetic signaling. Atropine removes direct muscarinic parasympathetic effector effects, but it does not exclusively isolate noradrenergic signaling, because nicotine still activates nicotinic receptors in both sympathetic and parasympathetic ganglia. Therefore, these experiments do not conclusively establish that the phenotype is driven by a pure noradrenergic neuronal population, particularly in light of the single-cell data showing mixed neuronal identities. As a result, the mechanistic basis of the pro-arrhythmic phenotype remains less specific than implied.

Reviewer #2

(Remarks to the Author)
I believe the authors have thoroughly responded to all prior comments. The revisions meaningfully address my prior concerns, and I support publication in the journal pending minor clarifications.

The expanded snRNA-seq analysis in Supplementary Figure 10, with HOX gene-based lineage assignment and three biological replicates, now provides sufficient characterization of the neuronal component within the fused iEHM. The dual normalization strategy for bulk RNA-seq and the revised Discussion distinguishing nicotine-induced neuronal activation from direct β-adrenergic stimulation also resolve my previous concerns. The framing of the iEHM as a tractable multicellular model rather than a faithful replica of native myocardium is appropriately calibrated.

Remaining minor points:

The characterization of Cluster 4 as transitional based on TH/CHAT/GATA3/ISL1 co-expression is plausible; the authors should briefly note whether this cluster's relative abundance was consistent across biological replicates, as variability would be relevant for interpreting the functional data.

The cardiomyocyte cluster heterogeneity acknowledged in Supplementary Figure 11B warrants a brief prospective comment in the Discussion on whether extended culture or subtype-directed differentiation could address this in future iterations of the model.

Open Access This Peer Review File is licensed under a Creative Commons Attribution 4.0 International License, which permits use, sharing, adaptation, distribution and reproduction in any medium or format, as long as you give appropriate credit to the original author(s) and the source, provide a link to the Creative Commons license, and indicate if changes were made.
In cases where reviewers are anonymous, credit should be given to 'Anonymous Referee' and the source.
The images or other third party material in this Peer Review File are included in the article's Creative Commons license, unless indicated otherwise in a credit line to the material. If material is not included in the article's Creative Commons license and your intended use is not permitted by statutory regulation or exceeds the permitted use, you will need to obtain permission directly from the copyright holder.
To view a copy of this license, visit https://creativecommons.org/licenses/by/4.0/

Point by Point response to the reviewers

We thank the reviewers for their time, insightful suggestions, and constructive criticism, which we believe have substantially strengthened this work.

Reviewer #1 (Remarks to the Author):

Schneider and colleagues present a 3D human innervated engineered heart muscle (iEHM) by fusing a sympathetic neuronal organoid (SNO) with engineered heart muscle (EHM) and apply this system to LQT2/KCNH2 disease modeling. The work is technically sound, and the data volume is substantial. The authors have made a serious effort to address the previous review, but the incremental novelty over existing approaches remains limited. Specifically,

1. As stated in my previous review, a number of groups have previously reported the generation of sympathetic neurons and their characteristics including most recently PMID 41386226. But what remains unclear in all these studies is a robust demonstration of physiological relevance including developmental origin. While the authors state that they made trunk neural crest-derived sympathetic neurons, they do not provide any proof. Moreover, given the heterogeneity in SNOs, which contain sensory, glutamatergic, and cholinergic neurons besides sympathetic neurons, it is important to demonstrate cell type composition of the progenitors as well as the end population. This has impact for disease modeling as the authors are evaluating a mixed population which may impact outcomes.

We thank the reviewer for this important comment. We agree that the developmental origin and cellular heterogeneity of the neuronal component are highly relevant for interpreting the physiological relevance of the model and its use in disease modeling.

Our SNO differentiation protocol was adapted from the previously established 2D protocol by Oh et al. (2016), which reported robust HOX gene expression consistent with trunk neural crest identity. In the original submission, we relied on this developmental framework and did not present this rationale with sufficient clarity. We have now clarified this point in the revised manuscript (Pg12, L29-37).

In addition, to address the reviewer's concern regarding the neuronal populations present in the disease model actually analyzed in this study, we have added new single-nucleus RNA-seq data from the iEHM in Supplementary Figure 10. These data provide a more detailed characterization of the neuronal populations present after fusion, including autonomic, cholinergic, noradrenergic, and sensory-like components (Pg6, L13-21, Pg12, L29-37).

We agree that direct profiling of the SNO progenitor population would provide complementary information. Because the present study is centered on the fused iEHM model and its functional consequences, we prioritized characterization of the final integrated system. We now explicitly acknowledge this in the revised Discussion (Pg11, L20-22).

To better position our work relative to recently published systems (PMID 41386226), we have also clarified the aspects in which our model extends beyond prior reports, including the vascularized and innervated 3D architecture, the ability to assess contractile performance in addition to beating rate, the application to KCNH2-associated arrhythmia modeling, and the observation that sympathetic innervation cannot be fully mimicked by acute β-adrenergic stimulation alone (Pg11, L10-16).

2. There is no data presented on the single nucleus sequencing of neurons except a UMAP in Figure 1D and some violin plots of ion channel genes in Supplementary Figure 10, which represents all neurons and not just sympathetic neurons. For transparency, and to address the issues mentioned above, the authors should provide single cell/nucleus gene expression profiles of SNOs and give an account of the cell composition.

We thank the reviewer for this helpful suggestion. We agree that a more transparent and unbiased account of neuronal composition is important.

To address this point, we have expanded the snRNA-seq analysis and now include data from three biological replicates, which allowed more robust resolution of neuronal subclusters in the iEHM. These new data are presented in Supplementary Figure 10 and identify multiple neuronal populations, including noradrenergic, cholinergic, sensory-like (glutamatergic), and additional autonomic neural crest-related clusters. We also include marker-based interpretation of these clusters together with HOX gene expression patterns to better define their likely identities.

We fully acknowledge that this analysis was performed on the fused iEHM rather than on isolated SNOs. We did not perform single-cell or single-nucleus profiling on SNOs alone in the present study, and we now state this explicitly in the revised manuscript (Pg11, L20-22). Because our main objective was to define the cellular composition of the final innervated cardiac model used for functional experiments, we focused our additional analysis on the iEHM.

Regarding the reviewer's question about the proportion of neurons that can be classified as sympathetic, our revised text now distinguishes more clearly between autonomic neuronal populations and the subset with noradrenergic features, while also acknowledging that some clusters remain incompletely resolved (Pg6, L13-21, Pg12, L29-37).

For example, based on Figure 1, there are an estimated 50 – 60% PHOX2B positive cells. This includes sympathetic neurons as well as any potential parasympathetic or cholinergic neurons. So, what is the % of neurons that can be classified as truly sympathetic? The authors should provide differential gene expression analysis of all clusters obtained from SNOs. They show selected marker expression by qPCR in supplemental figure 4A. Why can't this be done in a more unbiased way using their single cell data?

Specifically, the new data in Supplementary Figure 10 show that eight weeks after fusion, the iEHM contains peripheral autonomic identities in clusters 1, 4, 6, and 7, marked by *PRPH*, *CHRN3*, P*HOX2A/B*, and *ASCL1*, as well as non-autonomic identities in clusters 2, 3, and 5. Cluster 6 expresses *PHOX2B*, *TH*, and *SLC18A2* and is therefore most

consistent with a noradrenergic population, whereas cluster 1 expresses *CHAT* and represents a cholinergic component. Cluster 4 expresses both *TH* and *CHAT* together with *GATA3* and *ISL1*, suggesting a more immature population of mixed or transitional identity.

Moreover, HOX gene expression showed that *PHOX2A/B* marked the autonomic lineage (Clusters 1, 4, 6, 7), *HOXA3*, *HOXB3* defined vagal neural crest identity (Clusters 1, 4, 7) and *HOXB5*, *HOXD8* indicated a more posterior trunk neural crest identity (Cluster 6).

Such an analysis would also resolve whether cholinergic neurons are pre-ganglionic sympathetic or rather parasympathetic arising from cardiac neural crest cells.

The co-expression of *PRPH*, *CHRNA3*, *PHOX2A*, *KCNQ5*, and *CHAT* is most consistent with peripheral cholinergic autonomic neurons and therefore suggests a postganglionic identity. This data is now introduced in Supplementary figure 10.

3. For comparing SNOs generated in this study, the authors differentiated neurons as reported in Kirino et al. I do appreciate this but as the authors acknowledge themselves, this was done ad hoc through collaboration with an external party, which is not a neuronal differentiation group. The authors should consider comparing the RNA sequencing profiles of sympathetic neurons generated in different studies in order to better position their approach and also provide a transparent account of how the different approaches weigh against each other. I acknowledge that not all studies have performed this analysis but there are a few studies mentioned in Supplementary table 1 have done this.

We thank the reviewer for this thoughtful suggestion. We agree that cross-study comparison of transcriptomic profiles from stem cell-derived sympathetic neuron protocols would be valuable for positioning the present approach within the field.

We have not performed a systematic comparative reanalysis of published RNA-seq datasets across studies in the current revision. Our aim in including the 2D comparison was to provide additional context requested during review rather than to establish a comprehensive benchmark across all available differentiation strategies.

To address the spirit of the reviewer's comment, we have strengthened the discussion of how our model differs from previously published systems, including its integrated vascularized and innervated 3D architecture, its ability to assess contractile performance and arrhythmogenesis, and its application to KCNH2-associated disease modeling. We have also added text acknowledging that a systematic transcriptomic benchmarking analysis across studies would be an important future direction (Pg11, L18-19).

4. The cardiomyocyte identities in EHM remain confusing. There appears to be similar expression of ventricular markers IRX4, MYL2, and MYH7 in atrial/pacemaker-like and the two ventricular clusters (Supplementary Figure 11B). It does appear that the atrial/pacemaker-like cluster expresses more HCN4, SHOX2 but taken with the point above, it is still very mixed and indicates a suboptimal differentiation approach. The authors should check whether this a line-specific issue or a protocol-dependent issue.

We thank the reviewer for raising this important point. We agree that the mixed identity of iPSC-derived cardiomyocyte populations requires careful interpretation. In our dataset,

the atrial/pacemaker-like cluster shows increased expression of nodal and atrial-associated regulators, including *HCN4* and *SHOX2*, together with relatively lower *MYL2* and *IRX4* expression compared with the ventricular clusters, supporting its classification despite residual overlap. At the same time, we agree that the overall population remains heterogeneous. We have not performed additional experiments in this revision to formally distinguish whether this heterogeneity is line-specific or primarily protocol-dependent. We now state this explicitly and discuss it as a limitation (Pg6, L25-28). We also note in the revised manuscript that mixed atrial-, ventricular-, and pacemaker-like populations are commonly observed with standard Wnt-modulation-based differentiation protocols, suggesting that the phenomenon is likely at least in part protocol-related (Galdos et al. 2023 eLife, Nakanishi-Koakutsu et al. 2024 Comm Biol).

The heterogeneous expression patterns detected among scRNA-seq cardiomyocyte clusters may, at least in part, arise from asynchronous maturation, in line with the EHM spontaneous beating observed at earlier culture stages that progressively declines and eventually stops. This represents a particularly interesting point that should be addressed systematically in future studies.

5. The title "Bioengineering of a human innervated cardiac muscle model" implies a novel engineering approach to create innervated cardiac tissue. Since the focus of the study is not on engineering itself but rather on functional integration and disease modeling, the title could be modified to reflect that.

We have now revised the title of our manuscript to: Innervated human cardiac muscle model reveals sympathetic drivers of *KCNH2*-associated arrhythmias.

Reviewer #1 (Remarks on code availability): I have not reviewed the code.

Reviewer #2 (Remarks to the Author):

The authors have comprehensively addressed most of my concerns with thoughtful revisions and additional analyses. I remain excited about the potential impact of their findings on cardiomyocyte maturation within the co-cultured 3D system. This aspect holds significant implications for advancing our knowledge in cardiac research. However, I would like to highlight a couple of points that still require clarification.

Firstly, I appreciate the authors' efforts to address my concerns, particularly their analysis of cardiomyocyte maturation and the detailed description of the EHM formation protocol. These enhancements significantly contribute to understanding their study and the complexities of the neuro-cardiac interface.

We thank the reviewer for these encouraging comments and for recognizing the additional analyses and clarifications included in the revision.

The authors' approach to bulk RNA-seq analysis and the normalization of certain gene expressions, such as for CasQ2, appears somewhat unconventional. Further explanation of their rationale behind this normalization method would be beneficial for understanding the implications of their findings.

We thank the reviewer for requesting clarification of this point. In the original analysis, we normalized selected expression data to a cardiomyocyte-enriched reference gene because our intention was to assess changes relative to the cardiomyocyte compartment rather than to total mixed-tissue input. We chose this reference based on our prior work, in which it showed stable expression in cardiac tissue.

We agree, however, that this normalization strategy may not have been sufficiently explained and could appear unconventional without additional context. To improve transparency, we now provide the corresponding data normalized to a conventional housekeeping gene as well, and we clarify the rationale for both normalization approaches in the revised Methods and Results (Pg33, L12-16).

Additionally, I noted that the authors have not provided a thorough explanation for the observed reduction in heart rate (HR) variability following nicotine addition. Given that isoproterenol is expected to stimulate heart rate increases and reduce variability, clarity on the mechanisms in their model that led to these observations is crucial for a comprehensive interpretation of their results.

We thank the reviewer for highlighting this point. We realize that our original presentation may not have made this aspect sufficiently clear.

In our dataset (Figure 5), nicotine increased heart rate variability rather than reducing it. We have now revised the text to state this more clearly and to better distinguish the effects of nicotine-induced neuronal activation from those of direct β-adrenergic stimulation with isoproterenol.

In the revised Discussion, we further clarify our interpretation that these differing responses may reflect the release of additional neuron-derived factors beyond noradrenaline, including candidates such as NPY, which may modulate the

arrhythmogenic phenotype in ways not reproduced by isoproterenol alone (Pg13, L20-33). If the reviewer was referring to another specific panel or analysis, we hope that the revised presentation resolves the ambiguity.

Moreover, a comment regarding the composition of their iEHM system is warranted. While the inclusion of neural and cardiac components is intriguing, the relevance and consistency of this composition with in vivo heart conditions remain uncertain. A discussion around the implications of these cell types and their interactions within the context of cardiac physiology should be included to provide readers with a clearer understanding of the potential limitations of the model.

We thank the reviewer for this important comment. We agree that the cellular composition of the iEHM and its relationship to the in vivo heart should be discussed more explicitly, particularly with respect to both the strengths and the limitations of the model.

We have therefore added text to the revised Discussion clarifying that the model is not intended to fully recapitulate the exact cellular composition of native human myocardium. Rather, it is designed as a tractable human multicellular system to study neuro-cardiac interactions in a controlled setting. We also now discuss more explicitly how the presence of neural, vascular, mural, and cardiac populations may influence interpretation of the observed phenotypes (Pg11, L22-30).

Finally, although the authors introduced additional marker analyses for neuronal subtypes, the completeness of their profiling and characterization of cholinergic neuron populations seems insufficient. While they acknowledge the limitations in distinguishing among sympathetic and parasympathetic neurons, a more detailed exploration of these neuronal subtypes would strengthen the foundation of their findings.

We thank the reviewer for this constructive suggestion. To strengthen the characterization of neuronal subtypes, we now include expanded snRNA-seq analysis of the neuronal component in the iEHM in Supplementary Figure 10.

These data identify multiple neuronal subclusters, including populations with cholinergic, noradrenergic, sensory-like, glutamatergic, and autonomic neural crest-related signatures. We have revised the manuscript to describe these clusters more clearly and to explain how marker combinations and HOX gene expression informed our interpretation (Pg6, L13-21, Pg12, L29-37).

Specifically, the new data in Supplementary Figure 10 show that eight weeks after fusion, the iEHM contains peripheral autonomic identities in clusters 1, 4, 6, and 7, marked by *PRPH*, *CHRN3*, *PHOX2A/B*, and *ASCL1*, as well as non-autonomic identities in clusters 2, 3, and 5. Cluster 6 expresses *PHOX2B*, *TH*, and *SLC18A2* and is therefore most consistent with a noradrenergic population, whereas cluster 1 expresses *CHAT* and represents a cholinergic component. Cluster 4 expresses both *TH* and *CHAT* together

with *GATA3* and *ISL1*, suggesting a more immature population of mixed or transitional identity.

Moreover, HOX gene analysis showed that *PHOX2A/B* expression marked the autonomic lineage (Clusters 1, 4, 6, 7), *HOXA3, HOXB3* defined vagal neural crest identity (Clusters 1, 4, 7) and *HOXB5, HOXD8* indicated a more posterior trunk neural crest identity (Clusters 4, 6).

Point by Point rebuttal letter

We thank the reviewers for their time, insightful suggestions, and constructive criticism, which we believe have substantially strengthened this work.

Reviewer #1 (Remarks to the Author):

I appreciate the authors clarifications to the points raised in my previous assessment.

The newly provided single-cell data indicate that the neuronal compartment of iEHM is heterogeneous, with noradrenergic neurons representing only a minority and a substantial fraction of cholinergic or mixed-identity neurons. This raises an important question about how the reported high noradrenaline and low acetylcholine levels should be interpreted, and whether the disease phenotype in KCNH2 mutant tissues can be attributed specifically to sympathetic neurons.

The pharmacological data are consistent with an autonomic contribution and may reflect partly sympathetic signaling. Atropine removes direct muscarinic parasympathetic effector effects, but it does not exclusively isolate noradrenergic signaling, because nicotine still activates nicotinic receptors in both sympathetic and parasympathetic ganglia. Therefore, these experiments do not conclusively establish that the phenotype is driven by a pure noradrenergic neuronal population, particularly in light of the single-cell data showing mixed neuronal identities. As a result, the mechanistic basis of the pro-arrhythmic phenotype remains less specific than implied.

We appreciate the reviewer's concern. Although the noradrenaline measurements were performed in SNO at day 40, not in iEHM, where the neurons are at approximately day 90 we would like to underline that innervation cannot be assessed solely by cell number, since a single sympathetic neuron may innervate multiple cardiomyocytes, as also indicated by the immunofluorescence shown in Figure 2. In line with this point, the functional data obtained in the presence of atropine showed only a minor contribution of endogenous acetylcholine to iEHM beating rate (Figure 3g). Moreover, because the effect on beating rate was abolished by beta blockers, it is unlikely that other neurotransmitters played a substantial role in beating rate rather than noradrenaline. Last but not least, optogenetic stimulation of all neurons was performed in the absence of atropine (Figure 3e). In these experiments, the beating rate increased again in most tissues, consistent with a stronger influence of sympathetic neurons rather than parasympathetic.

For KCNH2 modeling our data support that noradrenaline is not the main contributor for the recorded pro-arrhythmic phenotype. Although beta adrenoreceptor blockade reduced iEHM beating rate also in the mutant tissues, it clearly did not alleviate the beating rate variability. We added in the discussion that other neuropeptides as for example NPY may be responsible for this phenotype. Defining the driver of arrhythmia in KCNH2 is a very interesting question which we plan to address in a future study employing this model. We have now changed the wording in the discussion that

addresses that the neurotransmitter responsible is not known and it should be investigated highlighted in green (Page 13).

Reviewer #2 (Remarks to the Author):

I believe the authors have thoroughly responded to all prior comments. The revisions meaningfully address my prior concerns, and I support publication in the journal pending minor clarifications.

The expanded snRNA-seq analysis in Supplementary Figure 10, with HOX gene-based lineage assignment and three biological replicates, now provides sufficient characterization of the neuronal component within the fused iEHM. The dual normalization strategy for bulk RNA-seq and the revised Discussion distinguishing nicotine-induced neuronal activation from direct β-adrenergic stimulation also resolve my previous concerns. The framing of the iEHM as a tractable multicellular model rather than a faithful replica of native myocardium is appropriately calibrated.

Remaining minor points:

The characterization of Cluster 4 as transitional based on TH/CHAT/GATA3/ISL1 co-expression is plausible; the authors should briefly note whether this cluster's relative abundance was consistent across biological replicates, as variability would be relevant for interpreting the functional data.

We thank the reviewer for raising this point. We now added a brief note about the relative abundance of this population across the samples on pg 6 (Highlighted in green)

The cardiomyocyte cluster heterogeneity acknowledged in Supplementary Figure 11B warrants a brief prospective comment in the Discussion on whether extended culture or subtype-directed differentiation could address this in future iterations of the model.

We thank the reviewer for this comment. We now added a small paragraph on the discussion that addresses the CM heterogeneity in the discussion on pg 11 (Highlighted in green).
