# Supplementary material for: Innervated human cardiac muscle model reveals sympathetic drivers of KCNH2-associated arrhythmias
Source: Nat Commun. 2026 Sep 2;17:9404. doi: 10.1038/s41467-026-76956-9 (PMC13538559; doi:10.1038/s41467-026-76956-9)
Supplement: Supplementary file 3 [file 41467_2026_76956_MOESM1_ESM.pdf]

Supplementary Information

Innervated human cardiac muscle model reveals sympathetic drivers of *KCNH2*-associated arrhythmias

#Lennart Valentin Schneider[1,2,3], #Michael Gani Setya[1,2,3], Guobin Bao[1], Daniel Härtter[1,2], Aditi Methi[4], Dennis Krüger[4], Marie Kristin Schreiber[1,3], Ole Jensen[5], Elisavet Kanari[1], Kea Aline Schmoll[1,3], Narasimha Swamy Telugu[6], Sadman Sakib[4], Eun Seo Kim[1], Aminath Luveysa Fahud[1], Maham Novin[1,2], Aylin Seedorf[1,2,3,7], Sebastian Diecke[6], André Fischer[3,4], Norman Y. Liaw[1,2], Wolfram-Hubertus *Zimmermann[1,2,3,4,8,9,10], *Maria-Patapia Zafeiriou[1,2,3,9,,11,12,13]

#Equal contribution, *Corresponding authors

[1] Institute of Pharmacology and Toxicology, University Medical Center Göttingen, Germany

[2] DZHK (German Center for Cardiovascular Research), partner site Lower Saxony, Germany

[3] MBExC (Multi-Scale Bioimaging Excellence Cluster), Göttingen, Germany

[4] German Center for Neurodegenerative Diseases, Göttingen, Germany

[5] Department of Clinical Pharmacology, University Medical Center Göttingen, Germany

[6] Max Delbrück Center for Molecular Medicine in the Helmholtz Association (MDC), Technology Platform Pluripotent Stem Cells, 13125, Berlin, Germany

[7] Department of Cardiology and Pneumology, University Medical Center, Göttingen, Germany

[8] Fraunhofer Institute for Translational Medicine and Pharmacology (ITMP), Göttingen, Germany

[9] German Center for Child and Adolescent Health (DZKJ), partner site Lower Saxony, Germany

[10] Campus-Institute Data Science (CIDAS), Göttingen, Germany

[11] Else Kröner Fresenius Center for Optogenetic Therapies (EKFZ L2T), Göttingen, Germany

[12] Clinic for pediatric and adolescent medicine, University Medical Center Göttingen, Germany

[13] Collaborative Research Center 1690, University of Göttingen, Göttingen, Germany

Supplementary Figure 1

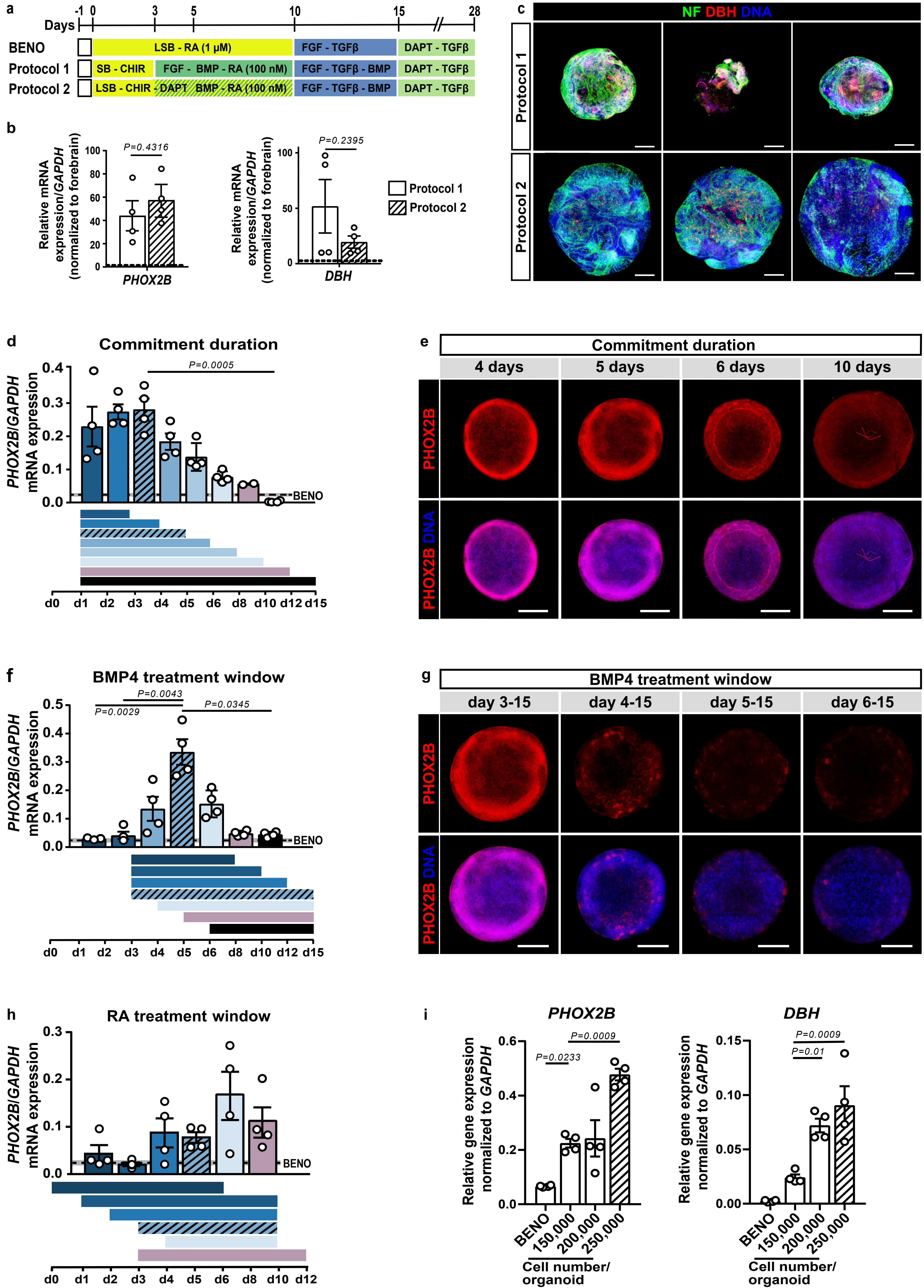

Supplementary Figure 1| SNO protocol optimization. **a** Schematic of two SN-differentiation protocols adapted from 2D-protocols (Kirino et al., 2018, Oh et al., 2016) to the BENO system. **b** *PHOX2B* (d15) and *DBH* (d30) expression in SNOs from both protocols, normalized to *GAPDH* and a forebrain organoid control (dashed line).1 differentiation, n=4. **c** WmIF of d30 SNO stained for sympathetic marker DBH (red), neural marker NF (green) and DNA (blue). Scale bar, 500 µm. **d** *PHOX2B* expression in d15 SNO with varying commitment durations based on protocol 2 (d1-10, see a), normalized to *GAPDH*. Treatment time windows are represented below with matching bar colors. 1 differentiation, n=4 (Except d1-d12 n=2). **e** WmIF of d15 SNOs stained for PHOX2B (red) and DNA comparing different commitment durations (10 days, see a). Scale bar, 500 µm. **f** *PHOX2B* expression with varying BMP4 treatment windows, normalized to *GAPDH* (d3-10, see a). 1 differentiation, n=4 (Except d3-d8 n=3). **g** WmIF of d15 SNO stained for PHOX2B (red) and DNA comparing different BMP4 timing (starting d3, see a). Scale bar, 500 µm. **h** *PHOX2B* expression SNO (d15) using varying RA treatment windows, normalized to *GAPDH* (d1-10, see a). 1 differentiation, n=4. **i** *PHOX2B* (d15) and *DBH* (d30) expression in SNO using different casting cell number, normalized to *GAPDH* (standard condition was 150,000 cells). 1 differentiation, n=4. Hashed bars indicate the new optimal condition. Data shown as mean ± s.e.m. with individual data points. One-way ANOVA or Kruskal-Wallis test with Dunn's multiple comparisons post hoc test was used for (**d**, **f**, **h**, **i**). Two-tailed unpaired Student's t-test or Mann-Whitney test was used for (**b**).

Supplementary Figure 2

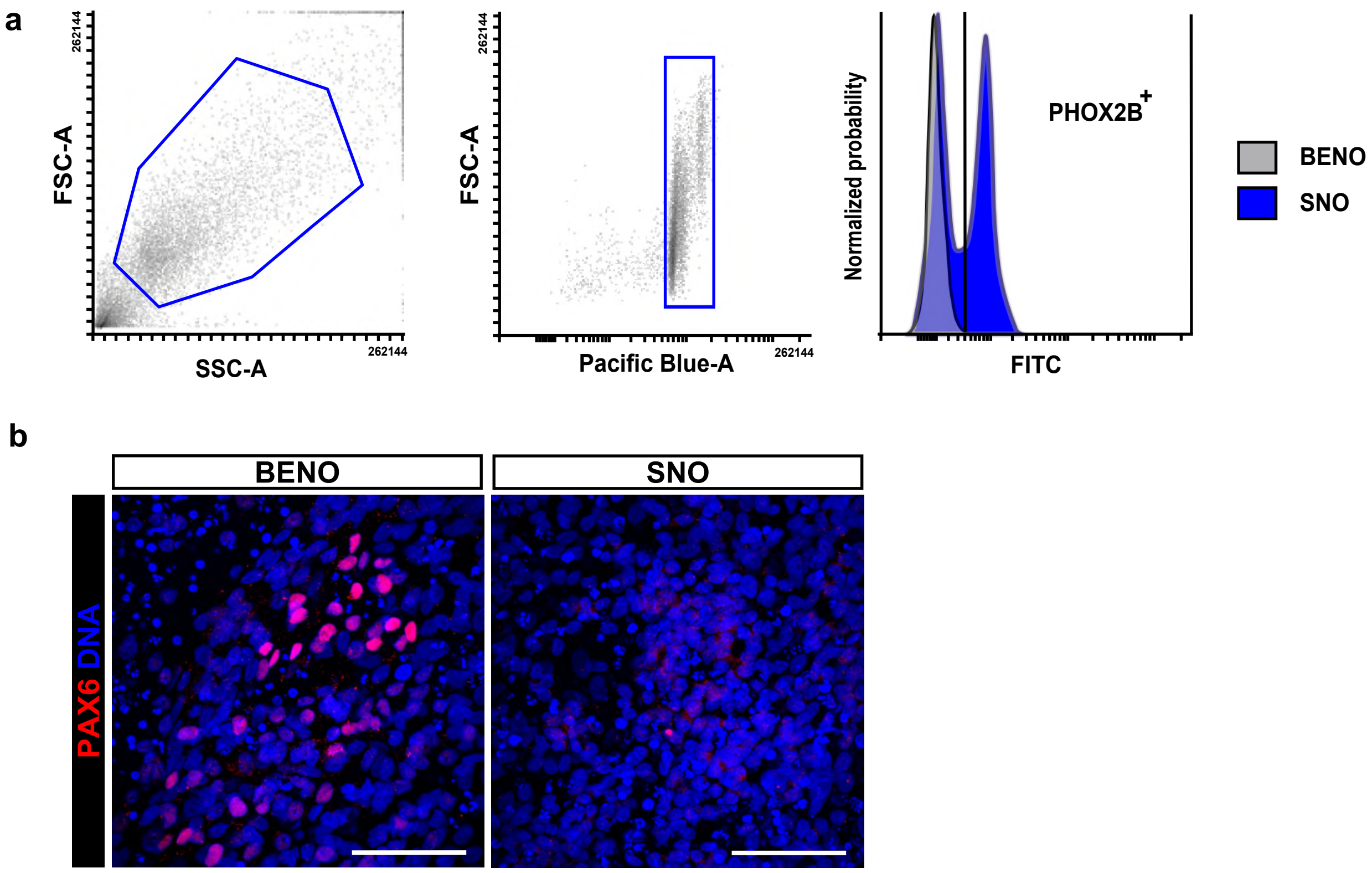

Supplementary Figure 2| Sympathetic neural progenitor analysis in d15 SNO. a Gating of flow cytometry analysis of d15 neural progenitor cells isolated from SNO (blue) and BENO (grey) stained for DNA and PHOX2B. SSC-A=side scatter-area, FSC-A=forward scatter-area. The right panel is the same as Figure 1b. **b** Immunofluorescence of d15 SNO and BENO stained for cortical marker PAX6 (red) and DNA (blue). Scale bar, 50 µm.

Supplementary Figure 3

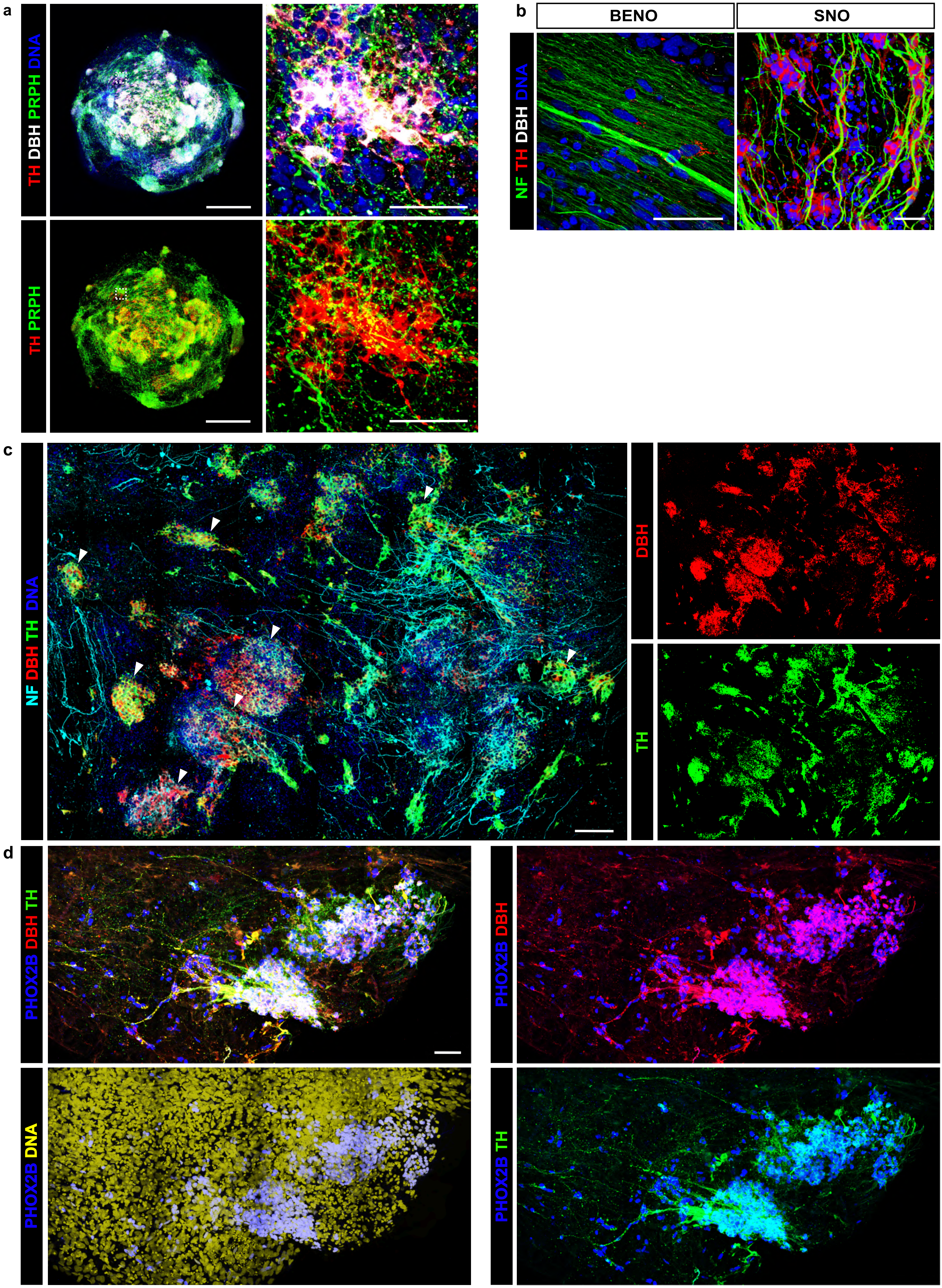

Supplementary Figure 3| Sympathetic neuron immunofluorescence analysis in SNO. **a** Representative image of d41 SNO stained for sympathetic marker TH (red), DBH (grey), peripheral neuron marker PRPH (green), and DNA (blue). Scale bar, 500 μm. (Right) Close-up view. Scale bar, 50 μm. **b** Close-up view shows SNs co-stained by TH (red), DBH (grey), NF (green) and DNA (blue) in SNO showing ganglia-like structures (Right). BENO did not contain SNs (Left). Scale bars, 50 μm. **c** High resolution imaging of SNO stained for sympathetic marker TH (green) and DBH (red) as well as NF (cyan) and DNA (blue) showing widespread sympathetic ganglia-like structures (marked by white arrowheads). Scale bar, 100 μm. **d** Close-up view of d93 SNO stained for sympathetic marker PHOX2B (blue), TH (green), DBH (red), and DNA (yellow). Scale bar, 50 μm.

Supplementary Figure 4

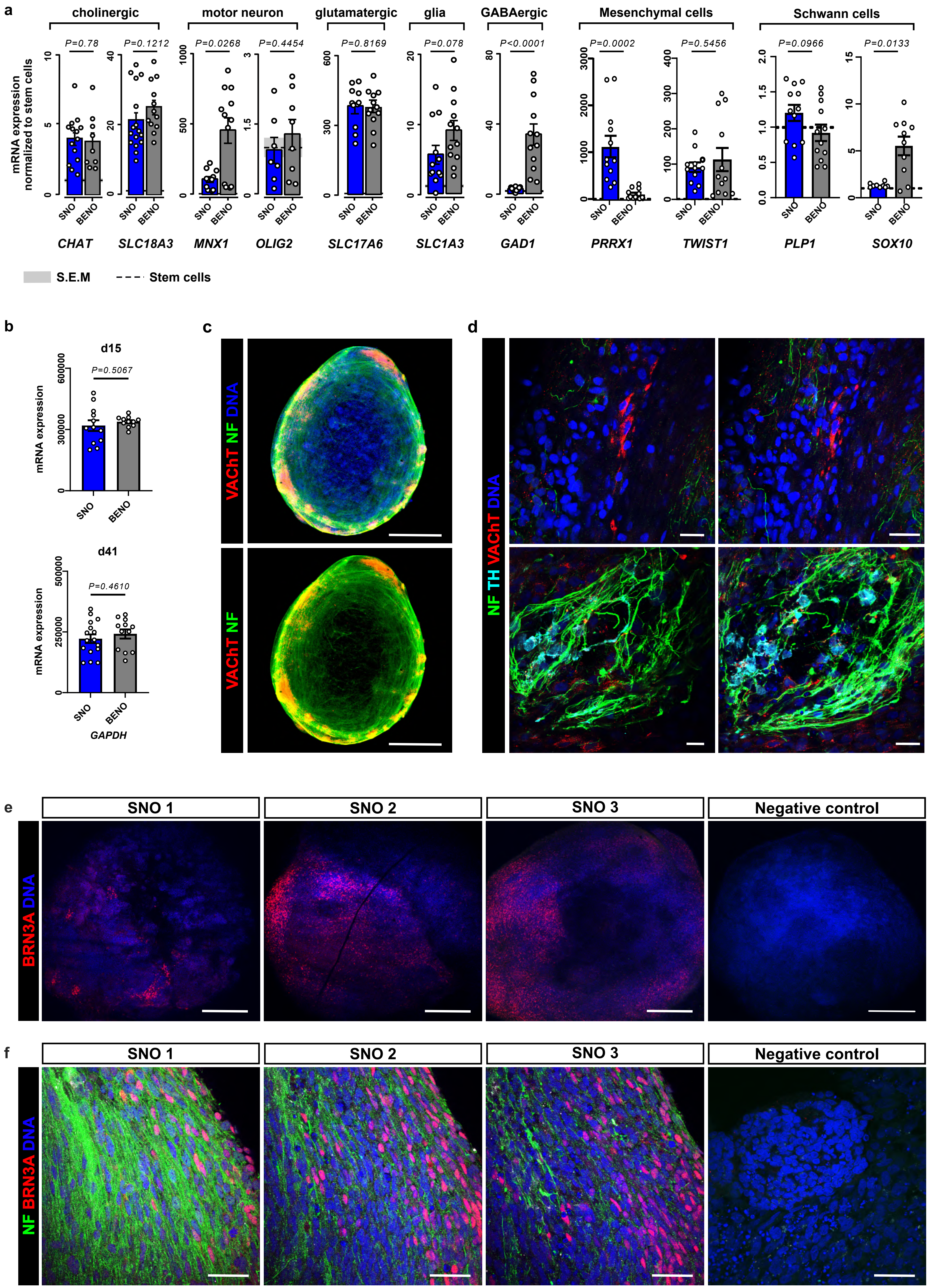

Supplementary Figure 4| Identification of non-SN cells co-developing in SNO. a Expression analysis of cholinergic marker (*CHAT, SLC18A3* [aka *VAChT*]), motor (*MNX1*, *OLIG2*), glutamatergic (*SLC17A6* [aka *VGLUT2*]), GABAergic (*GAD1*) neuron marker, astrocyte (*SLC1A3* [aka *GLAST*]) marker, mesenchymal (*PRRX1*, *TWIST1*) markers, and Schwann cells (*PLP1*, *SOX10*) markers for SNO and BENO at d41 normalized to *GAPDH* expression. SNO and BENO were normalized to expression of undifferentiated stem cells. 3-4 independent differentiations, 3-4 tissues per differentiation (n=11-16). *CHAT*: n(SNO)=14 n(BENO)=9. *SLC18A3*: n(SNO)=16 n(BENO)=12. *MNX1*, *SLC17A6*: n(SNO)=11 n(BENO)=12. *OLIG2*: n(SNO)=7 n(BENO)=8. *SLC1A3*, *GAD1*, *TWIST1*, *PLP1*: n(SNO/BENO)=12. *PRRX1*: n(SNO/BENO)=10. *SOX10*: n(SNO)=9 n(BENO)=10. **b** *GAPDH* expression level of d15 (Top) and d41 (Bottom) SNO and BENO. 3 independent differentiations, 3-4 tissues per differentiation (n=11-16). d15: n(SNO)=12 n(BENO)=11, d41: n(SNO)=16 n(BENO)=12. **c** WmIF of d30 SNO stained for cholinergic marker VAChT (red), NF (green) and DNA (blue). Scale bar, 500 μm. **d** Close-up view of cholinergic neurons VAChT (red) and SNs marked by TH (cyan) from two different SNO regions. Scale bar, 20 μm. **e** IF-staining of three individual SNO at d41 for sensory neuron marker BRN3A (red) and nuclear counterstaining (blue). (Far right) d41 SNO as no primary antibody control. Scale bar, 200 μm. **f** IF of three planes from a representative region of d41 SNO stained for sensory neurons (BRN3A (red) and NF (green)). No primary antibody control can be seen on the right. Scale bar, 20 μm. Data shown as mean ± s.e.m. with individual data points. Two-tailed unpaired Student's t-test or Mann-Whitney test was used for (**a**, **b**).

Supplementary Figure 5

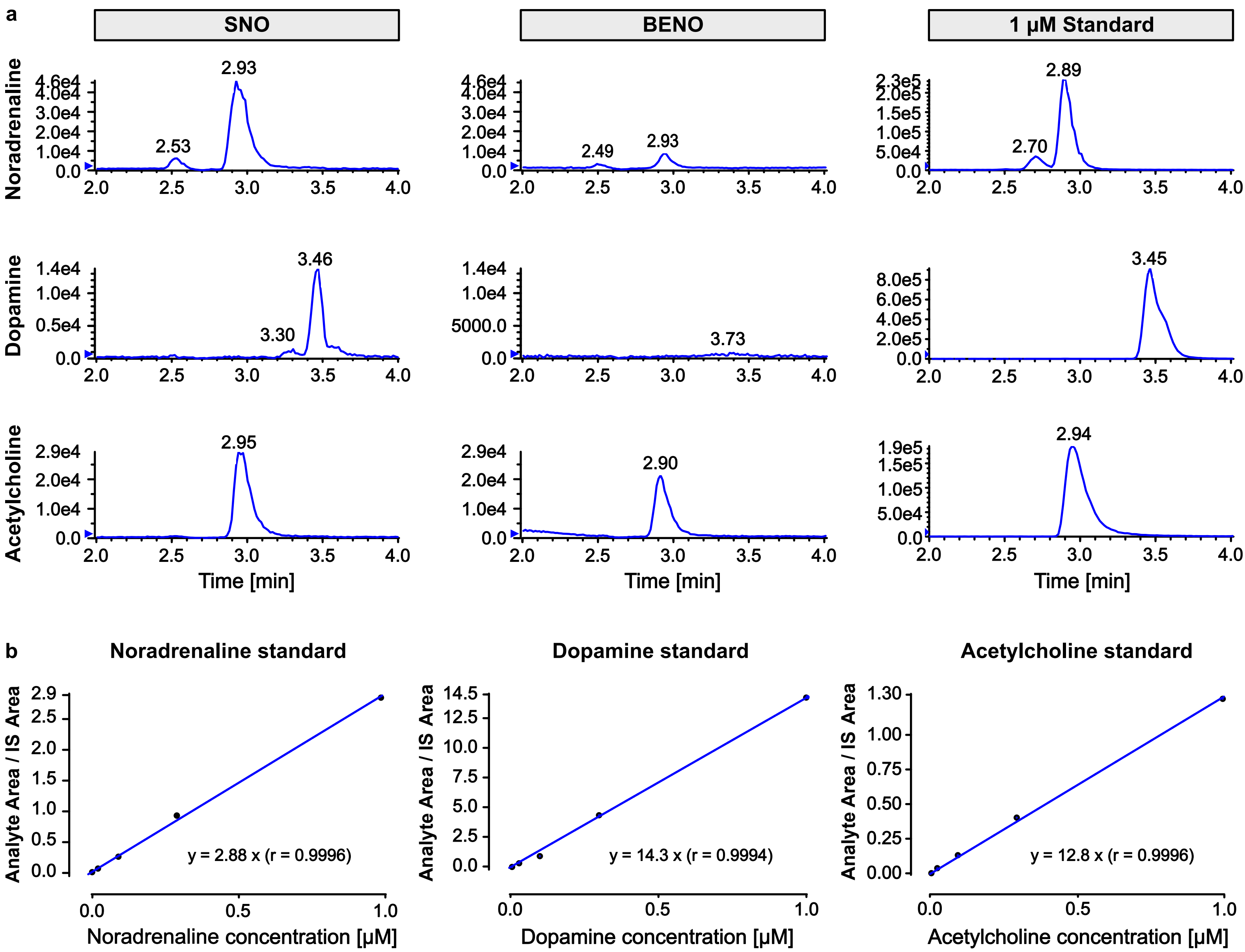

Supplementary Figure 5| Neurotransmitter quantification by LC-MS/MS analysis. **a** Representative LC-MS/MS spectra from lysed SNO and BENO for noradrenaline, dopamine, and acetylcholine compared to the respective standard at 1 µM concentration. **b** Standard curves of all three neurotransmitters (concentrations from 0.001 µM to 1 µM). Linear regression ("through zero") was calculated for the integrated peaks and used for quantification of the sample concentrations. Y-intercept and correlation coefficient r are indicated in the graphs.

Supplementary Figure 6

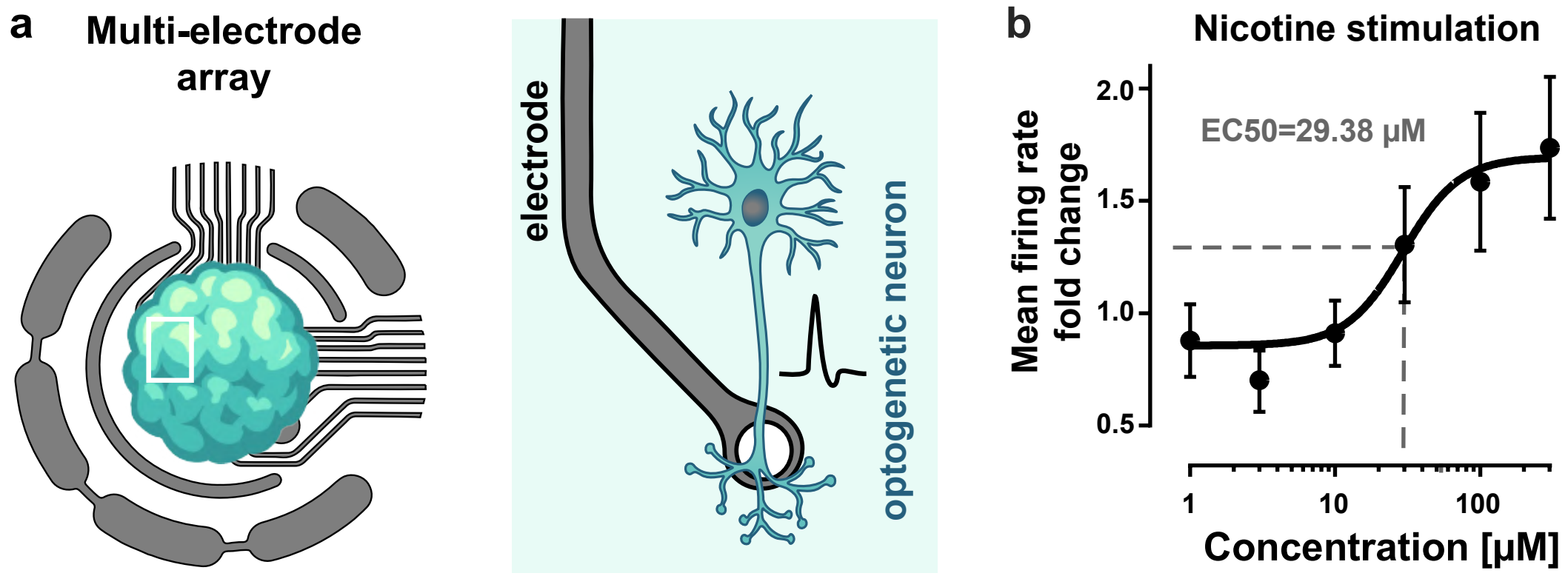

Supplementary Figure 6| Effect of nicotine on electrical activity of SNO d73-87. a Schematic of the placement of SNO on the multi-electrode array. **b** Dose response curve of mean firing rate of SNO upon stimulation with escalating concentrations of nicotine normalized to basal firing rate and untreated control. EC_{50} Nicotine=29.38 µM, Nonlinear fit using variable slope (four parameters, ordinary fit). 3 independent differentiations, 6-7 tissues per differentiation (n=19), mean ± s.e.m.

Supplementary Figure 7

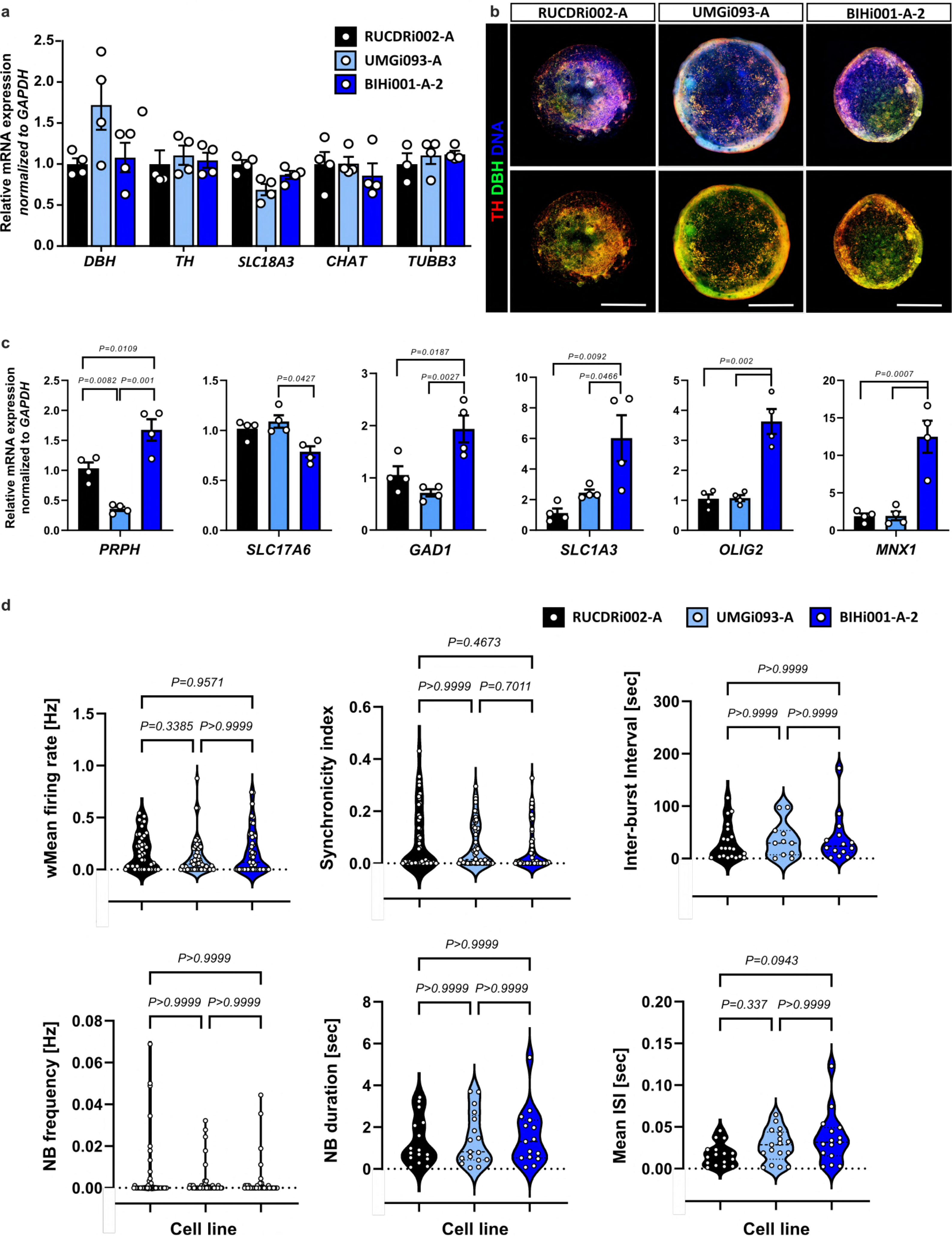

Supplementary Figure 7| Reproducibility of SNO differentiation protocol in three iPSC-lines. **a** qPCR analysis of SN-marker *DBH* and *TH*, cholinergic marker *SLC18A3* and *CHAT* as well as general neuron marker *TUBB3* between SNO from three different cell lines (RUCDRi002-A, UMGi093-A, BIHi001-A-2). Values were normalized to *GAPDH* and standard cell line RUCDRi002-A. 1 differentiation, n=4. **b** WmIF of SNO from three different iPSC lines stained for DNA (blue) and SN-marker DBH (green) and TH (red). Scale bar, 1 mm. **c** qPCR analysis of peripheral (*PRPH*), glutamatergic (*SLC17A6* [aka *VGLUT2*]), GABAergic (*GAD1*), motor (*MNX1*, *OLIG2*) neuron markers and astrocyte (*SLC1A3* [aka *GLAST*]) between SNO from three different cell lines (RUCDRi002-A, UMGi093-A, BIHi001-A-2). Values were normalized to *GAPDH* and standard cell line RUCDRi002-A. 1 differentiation, n=4. **d** Weighted (w) mean firing rate, synchronicity index, inter-burst interval, network burst (NB) frequency, NB duration and mean inter-spike-interval (ISI) of SNO from three different cell lines (RUCDRi002-A, UMGi093-A, BIHi001-A-2) weeks 4-8. 1 differentiation, n=16 / group. All data is presented as mean ± s.e.m. One-way ANOVA or Kruskal-Wallis test with Dunn's post hoc test was performed for (**a**, **c**, **d**).

Supplementary Figure 8

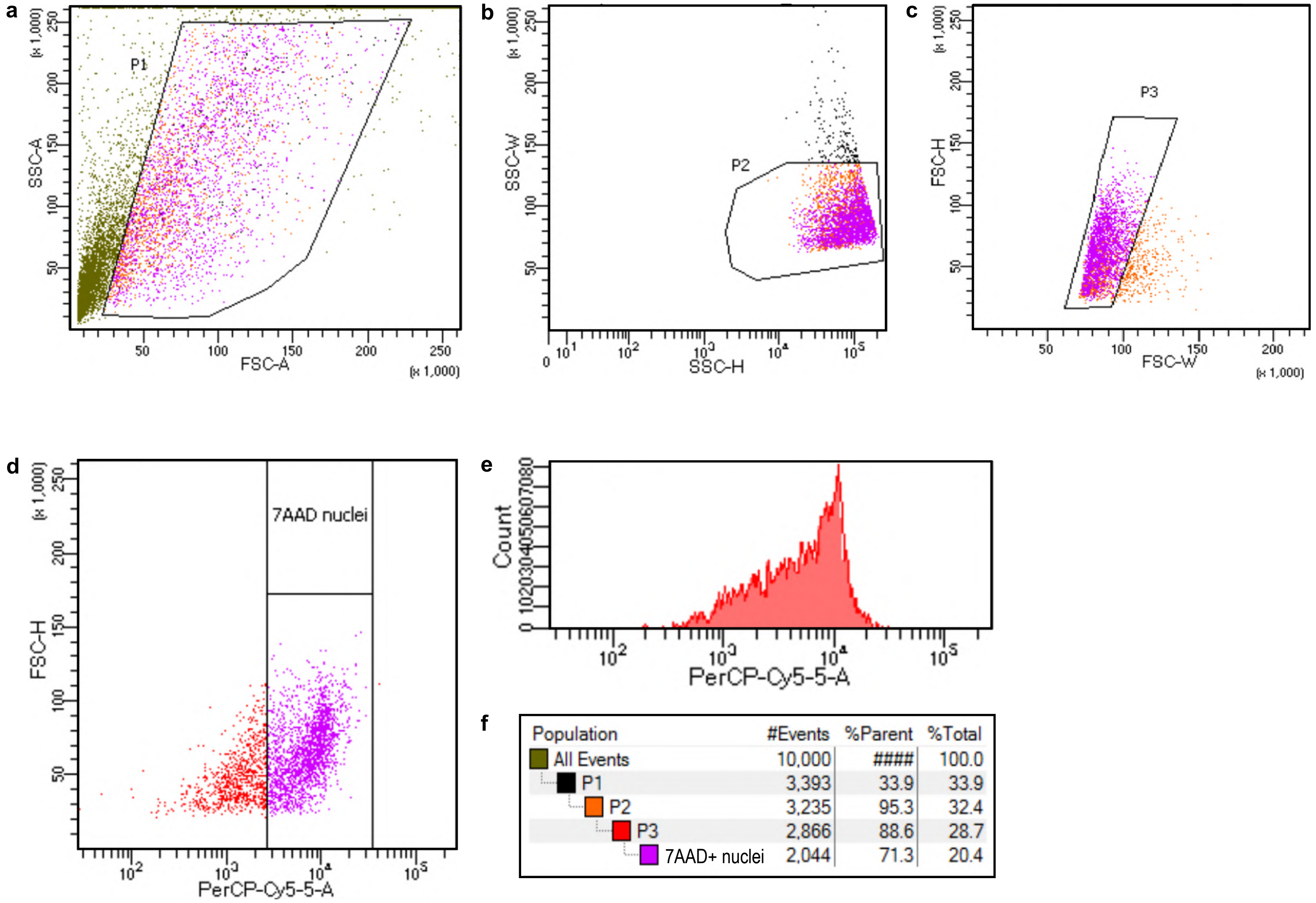

Population	#Events	%Parent	%Total
All Events	10,000	####	100.0
P1	3,393	33.9	33.9
P2	3,235	95.3	32.4
P3	2,866	88.6	28.7
7AAD+ nuclei	2,044	71.3	20.4

Supplementary Figure 8| Gating strategy for sorting nuclei from frozen iEHM lysates using 7AAD staining. a-c Gating strategy for exclusion of debris (P1, P2, P3) and for **d** 7AAD-stained nuclei from iEHM lysates. **e** Histogram of raw nuclei count (PerCP-Cy5.5 signal, 7AAD dye). **f** Hierarchy of the gated populations including proportions.

Supplementary Figure 9

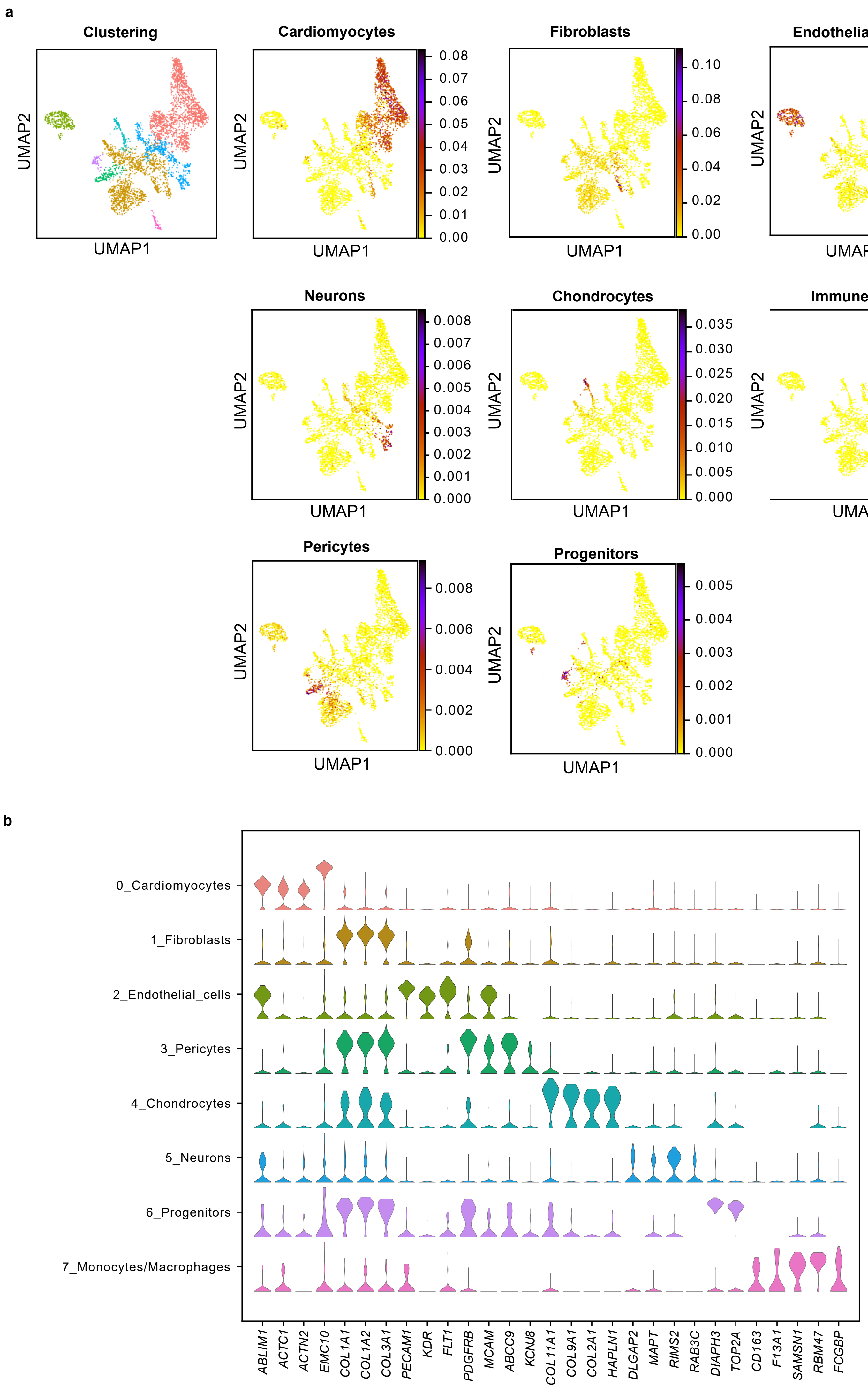

Supplementary Figure 9| Cell cluster annotation of snRNA-sequencing. a UMAP plots depicting the consensus expression (average module score) of cell-type specific marker genes used to annotate the clusters in the dataset. Gene names are highlighted in blue under the plot title of the corresponding cell-type. **b** Violin plots showing the normalized expression of individual marker genes from **a** across the annotated cell-types.

Supplementary Figure 10

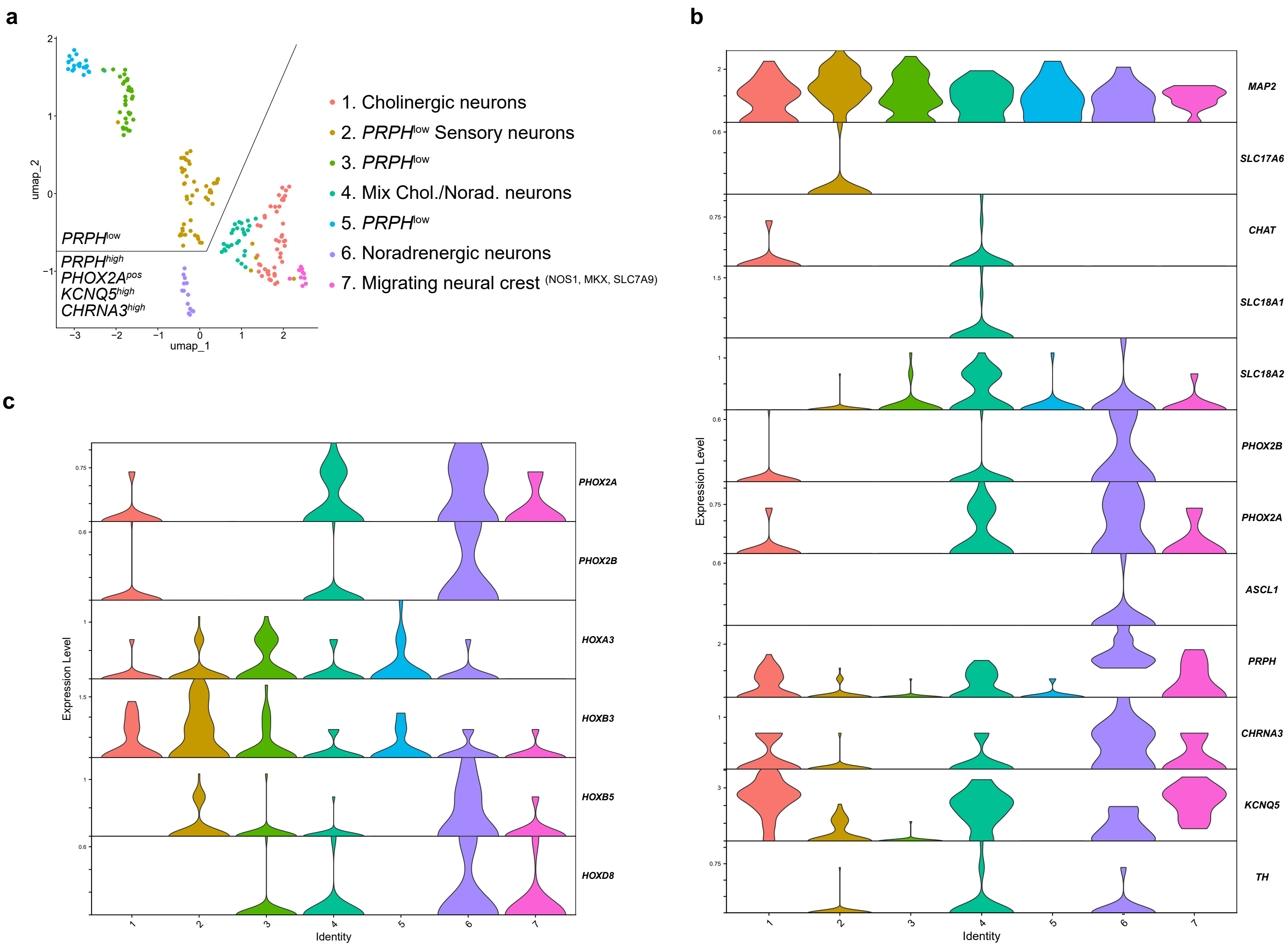

Supplementary Figure 10| Expression analysis of neuronal subclusters in iEHM by snRNA-sequencing. a UMAP visualization of neuronal subclusters identified in the dataset. Seven clusters were detected. Clusters 1, 4, 6, and 7 show high expression of peripheral autonomic markers *PRPH, KCNQ5, PHOX2A, CHRNA3,* whereas clusters 2, 3 and 5 display lower expression of these peripheral markers. **b** Violin plots showing the normalized expression of pan-neural marker MAP2; autonomic lineage *PRPH, ASCL1, PHOX2A/B, CHRNA3;* cholinergic marker *CHAT;* noradrenergic markers *TH* and *SLC18A1/2;* and glutamatergic marker *SLC17A6*. **c** Violin plots showing the normalized expression of HOX genes across neuronal clusters. *PHOX2A/B* mark the autonomic lineage, while *HOXA3, HOXB3* define vagal neural crest identity and *HOXB5, HOXD8* indicate a more posterior trunk neural crest identity.

Supplementary Figure 11

a

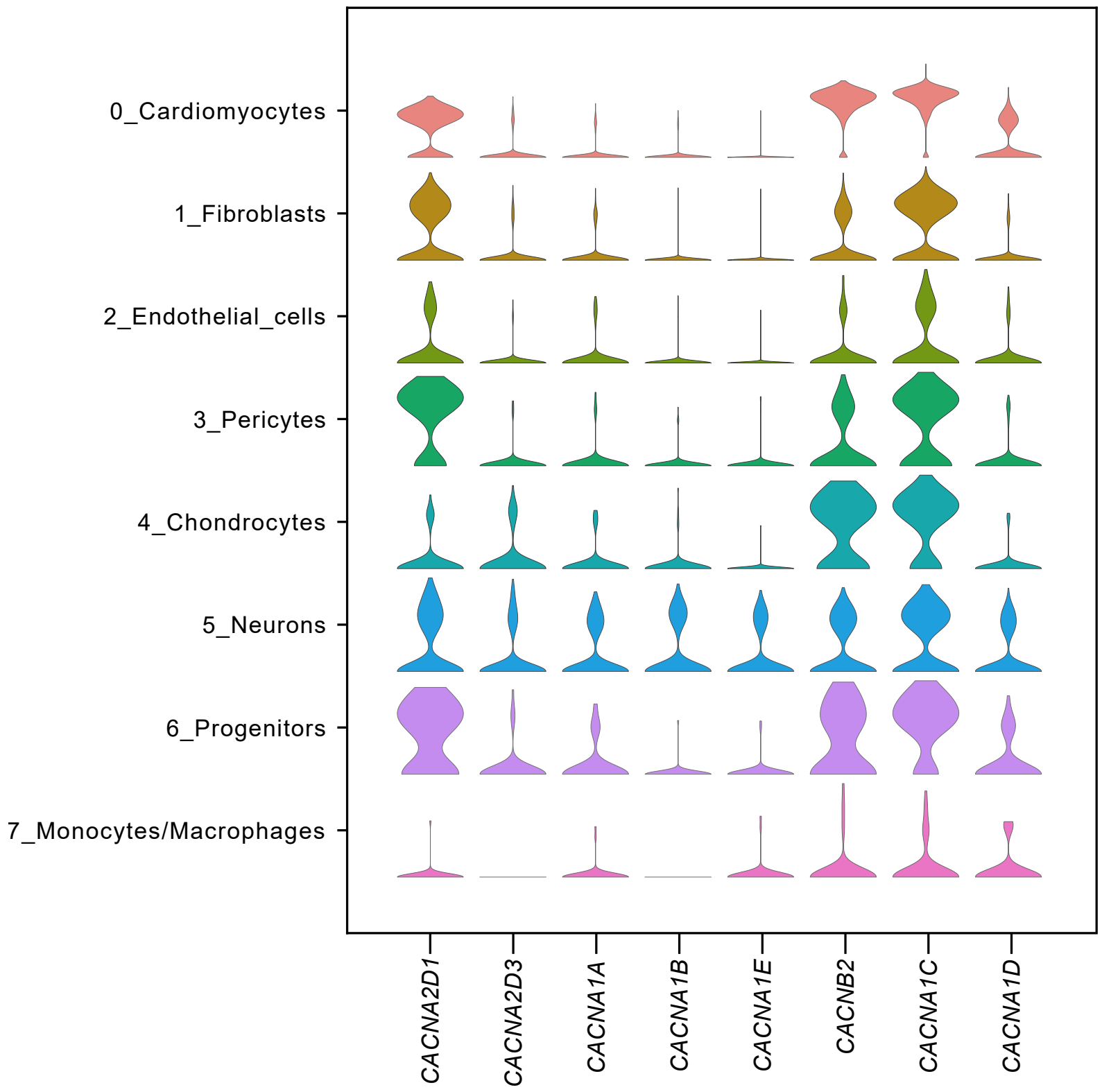

b

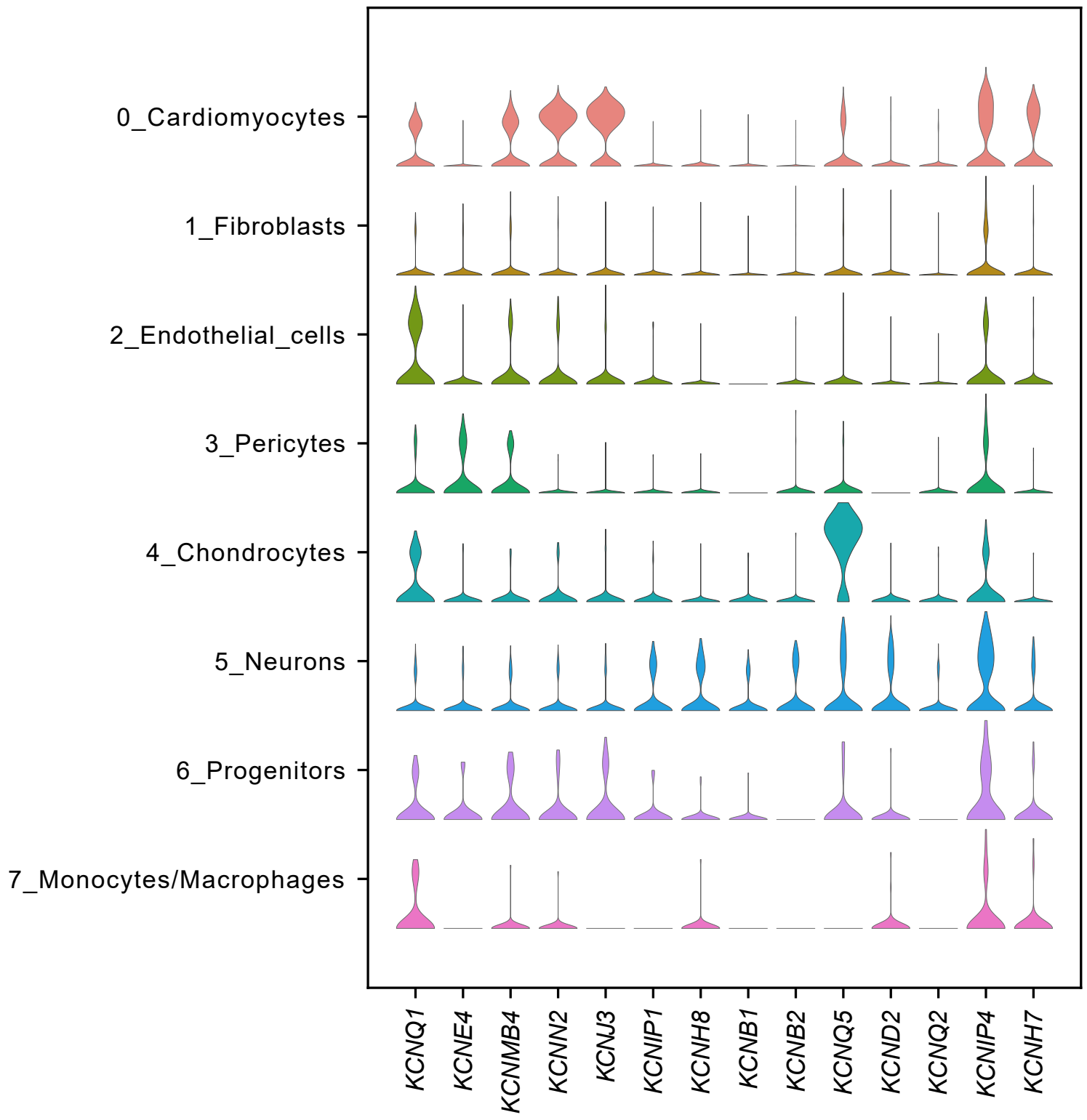

c

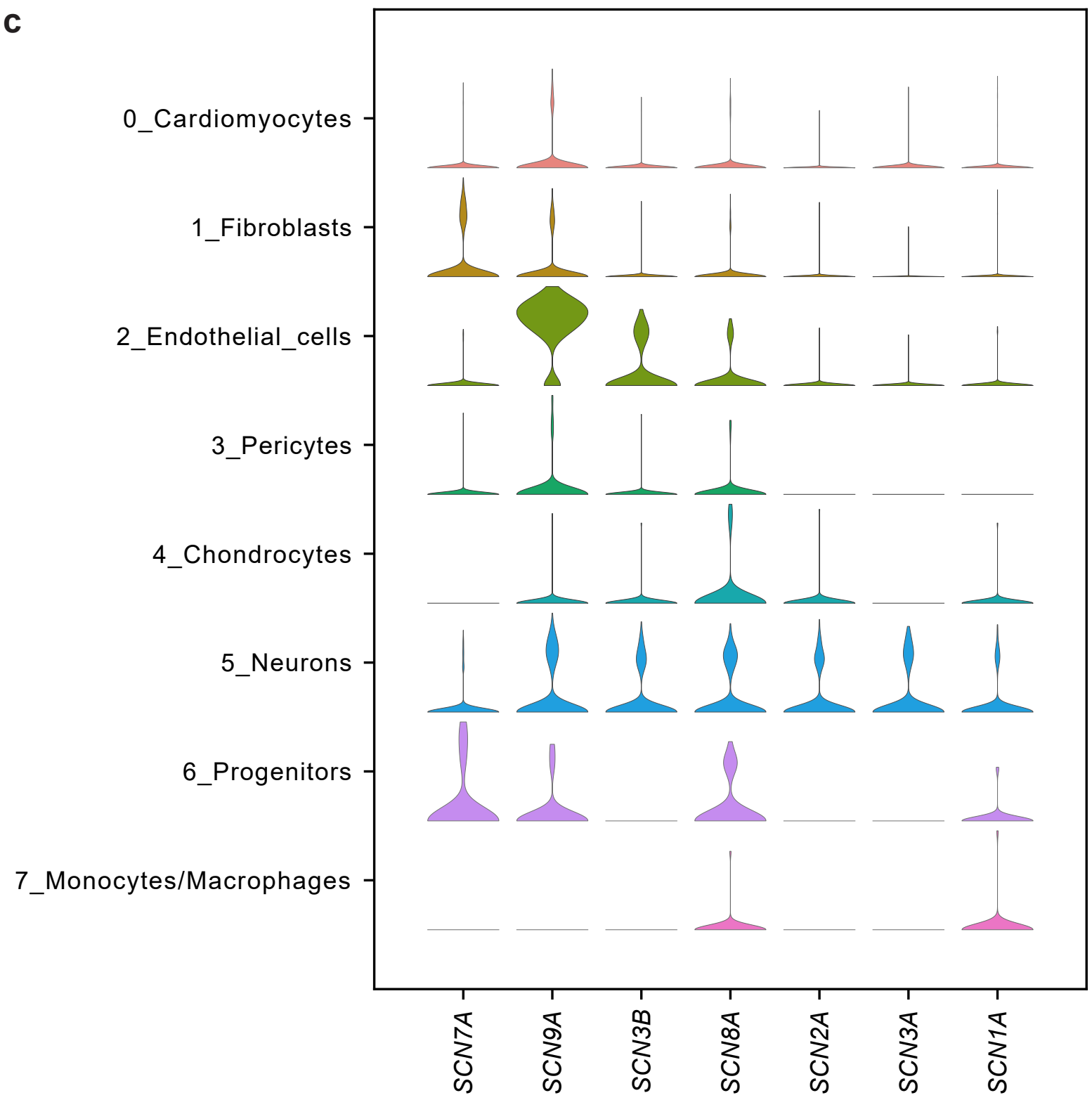

d

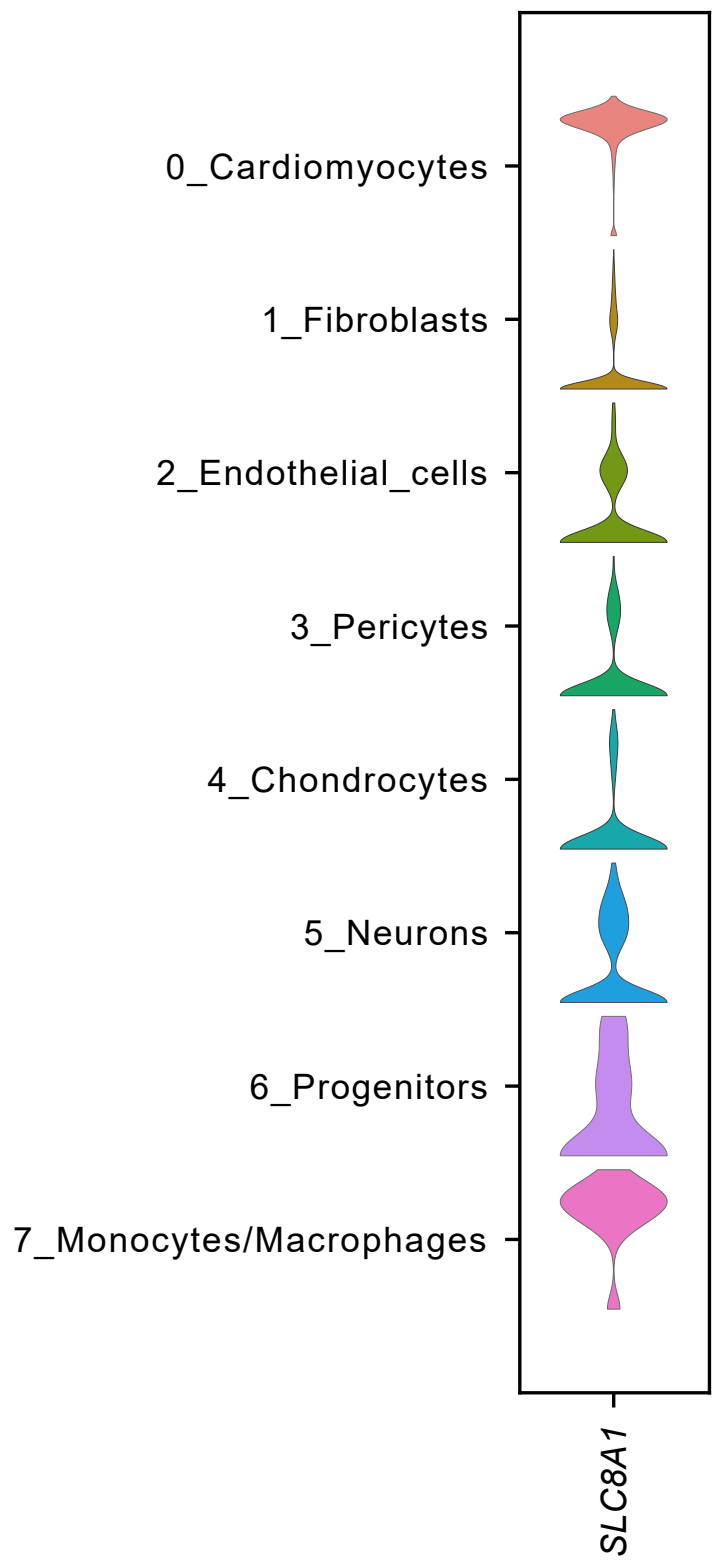

Supplementary Figure 11| Expression analysis of ion channels across cell types from snRNA-sequencing. Violin plots showing the normalized expression of **a** voltage-gated calcium (Ca^{2+}) channel transcripts, **b** voltage-gated potassium (K^{+}) channel transcripts, **c** voltage-gated sodium (Na^{+}) channel transcripts and **d** the Na+/Ca^{2+} exchanger *SLC8A1* transcript, across the annotated cell-types.

Supplementary Figure 12

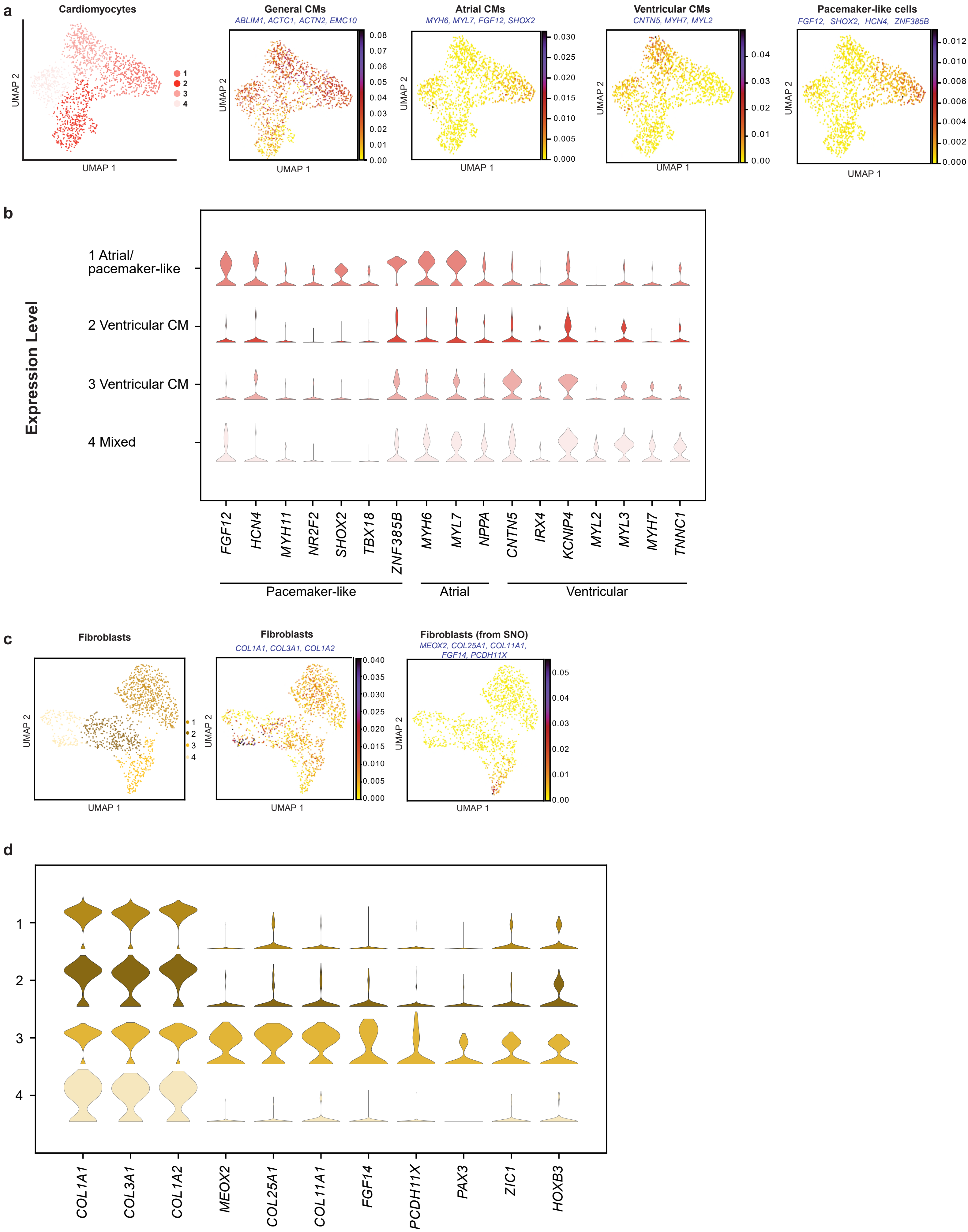

Supplementary Figure 12| Analysis of CM and fibroblast subcluster in the iEHM. a UMAP plots depicting the consensus expression (average module score) of atrial (*FGF12, MYH6, MYL7*), ventricular (*CNTN5, MYH7, MYL2*), pacemaker-like (*SHOX2*, *FGF12, HCN4, MYH11, ZNF385B*) and general CM marker genes across the CM sub-clusters (UMAP plot in the top-left panel). Gene names are highlighted in blue under the plot title of the corresponding sub-type. **b** Violin plots showing the normalized expression of individual atrial and ventricular CM marker genes from a across the three sub-clusters. **c** UMAP plots depicting the consensus expression (average module score) of fibroblast marker genes across the fibroblast sub-clusters (UMAP plot in the top-left panel). **d** Violin plots showing the normalized expression of individual fibroblast marker genes from c across the two sub-clusters.

Supplementary Figure 13

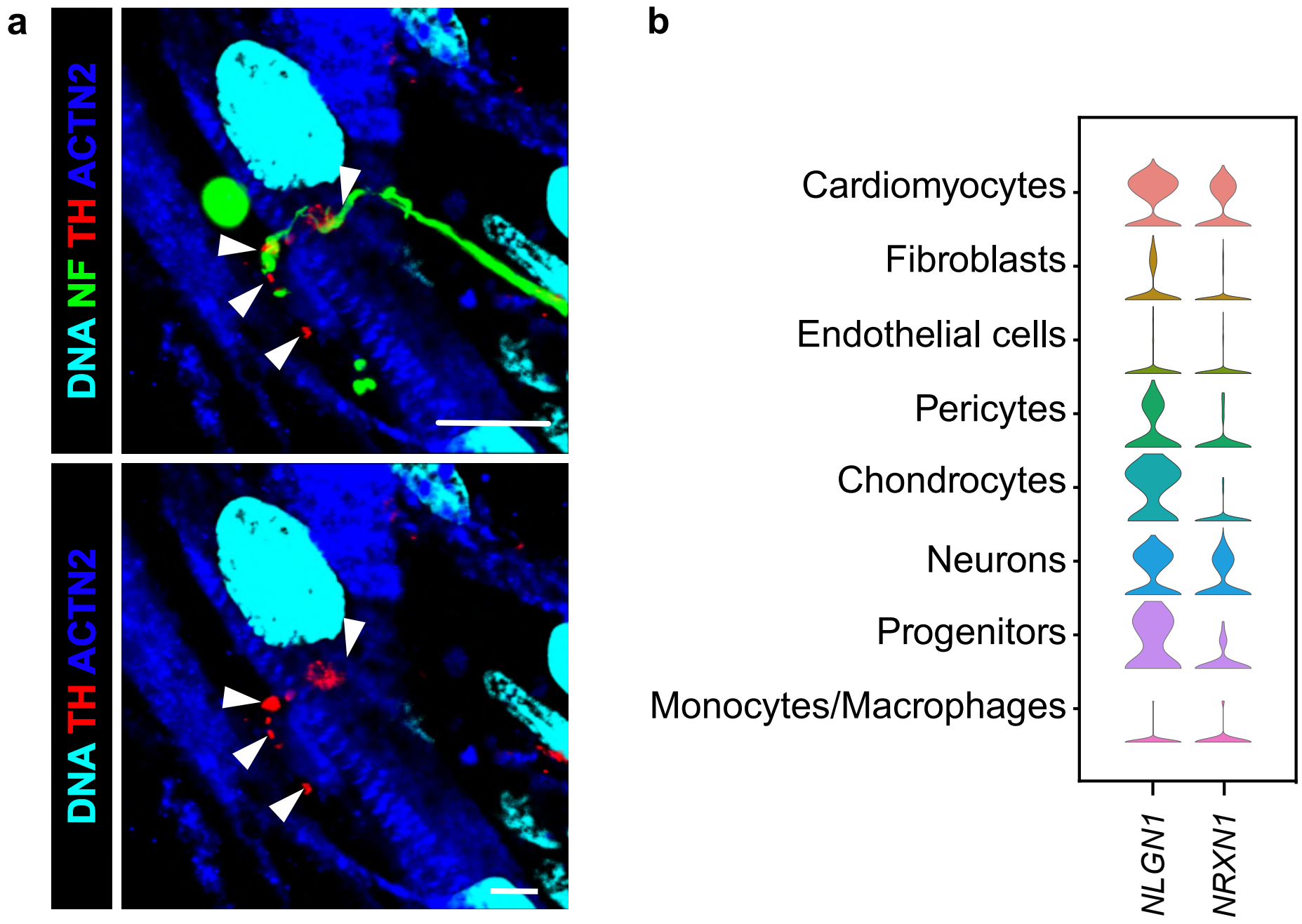

Supplementary Figure 13| Investigation of neuro-cardiac junctions in iEHM. a WmIF showing co-staining of synaptic bouton-like varicosity (NF, green) co-localizing with autonomic neuron marker TH (red) on the surface of CMs (ACTN2, blue) and DNA (cyan). Co-localization is indicated by white arrowheads. Scale bar, 10 µm. **b** snRNA sequencing expression analysis of presynaptic marker neurexin 1 (*NRXN1*) and postsynaptic adhesion molecule neuroligin 1 (*NLGN1*) in the annotated iEHM cell clusters (see Supplementary Figure 9).

Supplementary Figure 14

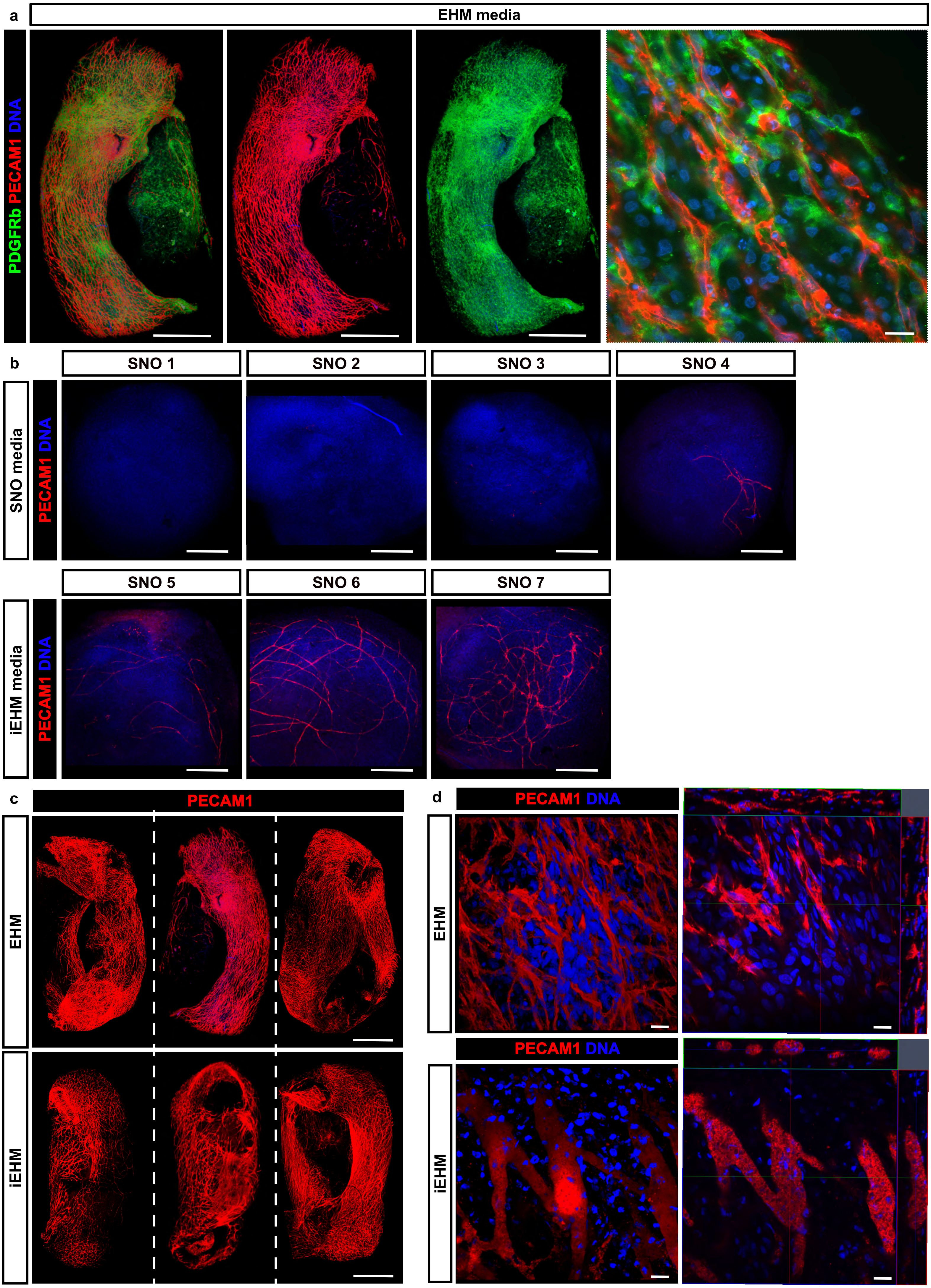

Supplementary Figure 14| Vascular network development in EHM, SNO and iEHM. a WmIF of 6 weeks EHM cultured in EHM medium reveals a dense endothelial network (PECAM1, red), presence of PDGFRb (green)-positive pericytes and DNA (blue). Scale bar, 1 mm. (Right) Close-up view showing close localization of pericytes and endothelial cells. Scale bar, 20 μm. **b** (Top) WmIF of d93 SNO cultured exclusively in SNO media shows presence of endothelial cells (PECAM1, red) in only one out of four tissues. (Bottom) After eight weeks of culture in iEHM medium, d103 SNO show the presence of endothelial cells (PECAM1, red) in all of the screened tissues. Scale bar, 200 μm. **c** PECAM1 (red) immunofluorescence analysis of three representative 6-week-old EHM and iEHM. Scale bar, 1 mm. **d** Close-up view of PECAM1 staining in EHM and iEHM (Left) and the 3D-reconstruction (Right) show a larger vessel diameter in some areas of the iEHM when compared to EHM. Scale bar, 20 μm.

Supplementary Figure 15

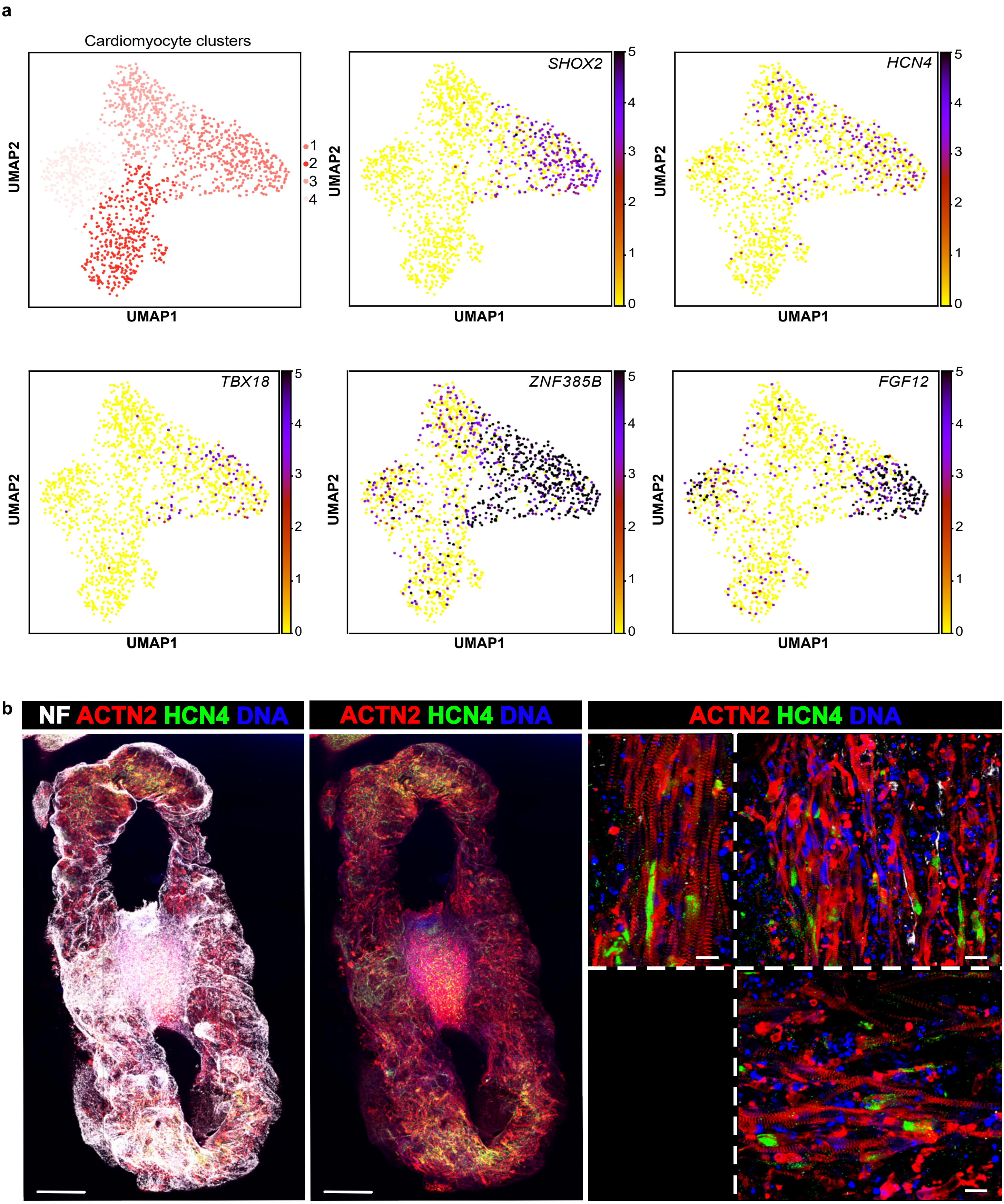

Supplementary Figure 15| Identification of pacemaker-like cells in iEHM. a UMAP plots showing the distribution of cells expressing the pacemaker-like-cell markers *HCN4, SHOX2, FGF12, TBX18,* and *ZNF385B* in the clusters in the dataset including cluster annotations (compare in the four CM cluster (Supplementary Figure 11). **b** WmIF of iEHM stained for NF (grey), CM marker ACTN2 (red), pacemaker-like-cell marker HCN4 (green) and DNA (blue). (Left) Overview images show pacemaker-like-cells dispersed throughout the innervated areas of the EHM. Scale bar, 500 µm. (Right) Close-up view show HCN4 positive cells in between ACTN2-positive CM co-localizing with NF-positive neurites (top left panel). Scale bar, 20 µm.

Supplementary Figure 16

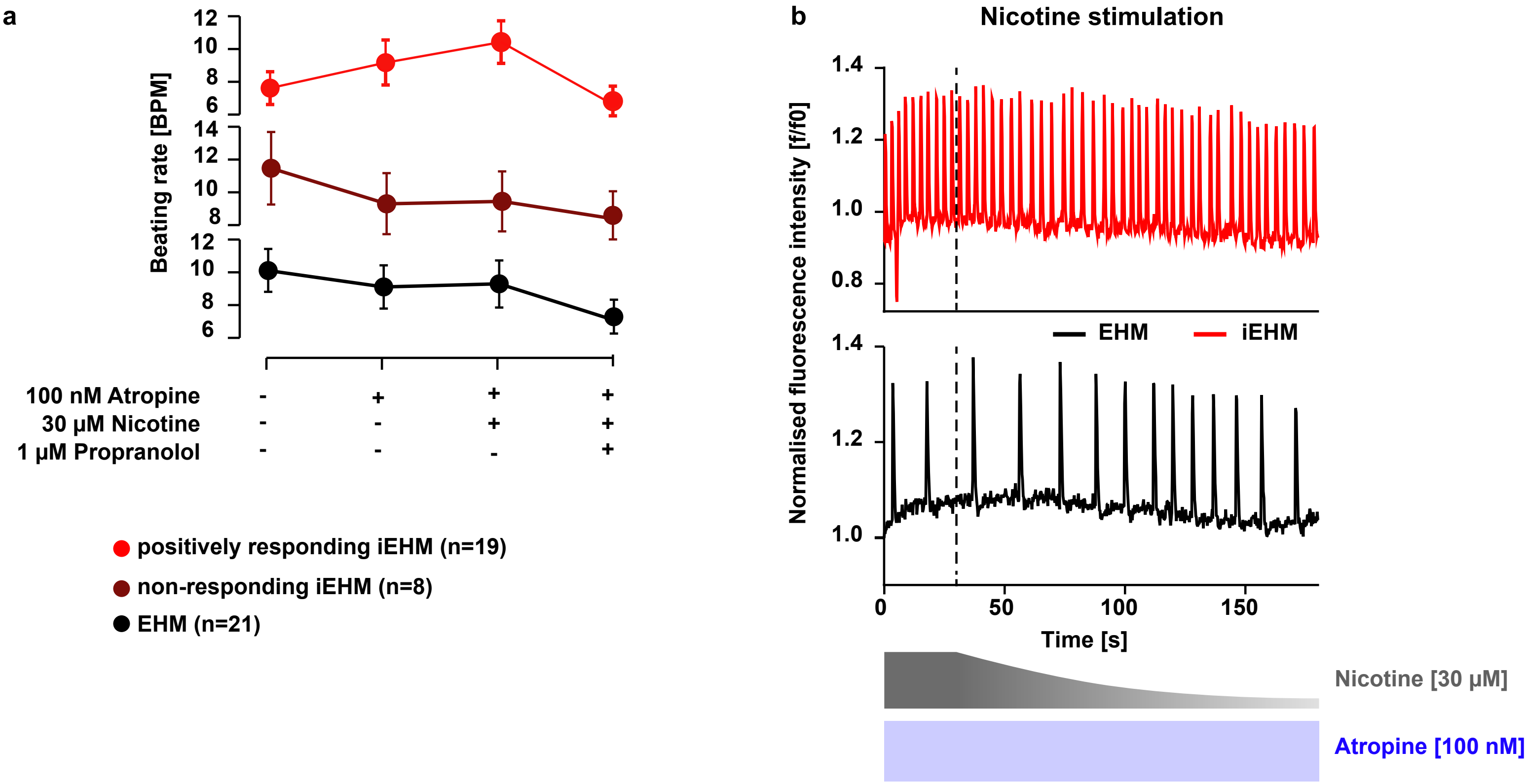

Supplementary Figure 16| Details of pharmacological stimulation of iEHM. a Chronotropic response to stimulation of EHM and iEHM with 100 nM atropine, 30 µM nicotine and 1 µM propranolol. iEHM could be separated into two distinct groups of responding (n=19) and non-responding tissues (n=8) that behaved similar to EHM controls (n=21). **b** Example traces showing the acute effect of nicotine stimulation on EHM and iEHM mean fluorescence intensity (f/f0) of the ROI (see in Figure 3f) in the presence of 100 nM atropine.

Supplementary Figure 17

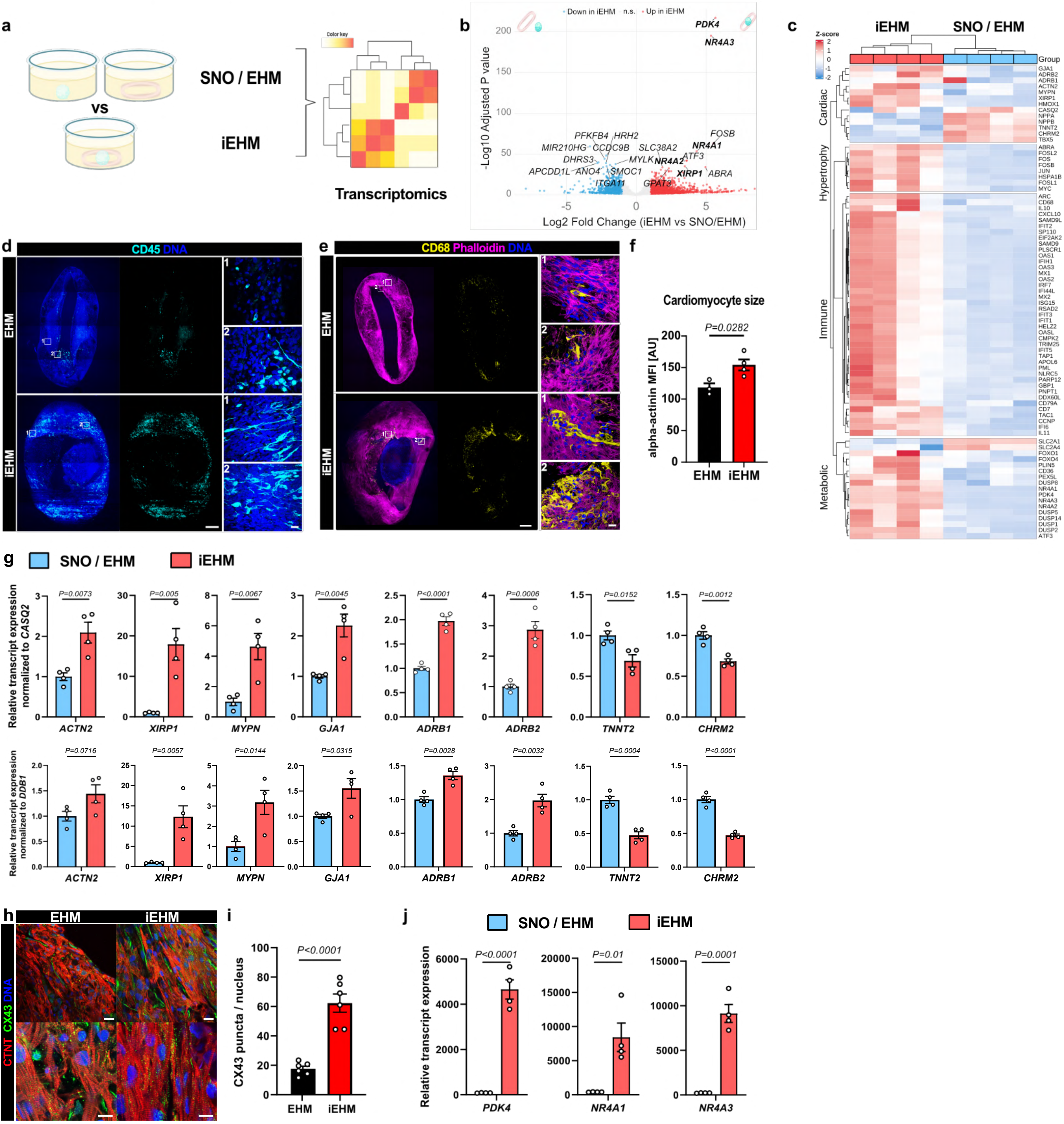

Supplementary Figure 17| Bulk RNA-sequencing of EHM+SNO vs iEHM. a Schematic diagram of bulk RNA-sequencing samples of pooled individual cultured neural (SNO) and cardiac (EHM) modules vs iEHM. **b** Volcano plot showing differentially expressed genes between iEHM and SNO+EHM organoids (adjusted P<0.05, $|\log_2 FC| > 1$). **c** Heatmap of representative differentially regulated genes linked to cardiac function, metabolism, immune signaling, and hypertrophy. **d** WmIF of d71 EHM and iEHM stained for hematopoietic progenitors (CD45, cyan) and DNA (blue). Scale bar, 500 µm. (Right) Close-up views of CD45 progenitor cells in EHM and iEHM. Scale bar, 20 µm. **e** WmIF of d71 EHM and iEHM stained for immune cell marker CD68 (yellow), CM marker Phalloidin (magenta) and DNA (blue). Scale bar, 500 µm. (Right) Close-up views of CD68 immune cells in EHM and iEHM. Scale bar, 20 µm. **f** Cardiomyocyte (α-actinin) size of digested EHM and iEHM. Size is assessed by median fluorescence intensity of α-actinin signal. n(EHM)=3 n(iEHM)=4. **g** Cardiac specific transcript expression levels normalized to (Top) *CASQ2* and (Bottom) *DDB1,* n=4 tissues / condition. **h** WmIF of d71 EHM and iEHM stained for gap junction marker CX43 (green), cardiac marker CTNT (red) and DNA (blue). Scale bar, 20 µm. **i** Quantification of CX43 puncta from 6 representative close-up images of EHM and iEHM normalized to the number of nuclei. **j** Relative transcript expression of *PDK4*, *NR4A1*, and *NR4A3* in EHM/SNO vs iEHM. n=4 tissues / condition. All data is presented as mean ± s.e.m. Unpaired two-tailed Student's t-test was used for (**f**, **g**, **i**, **j**). Supplementary Figure 17a created in *BioRender*. Zafeiriou, M. P. (2026) https://BioRender.com/w8pgnup.

Supplementary Figure 18

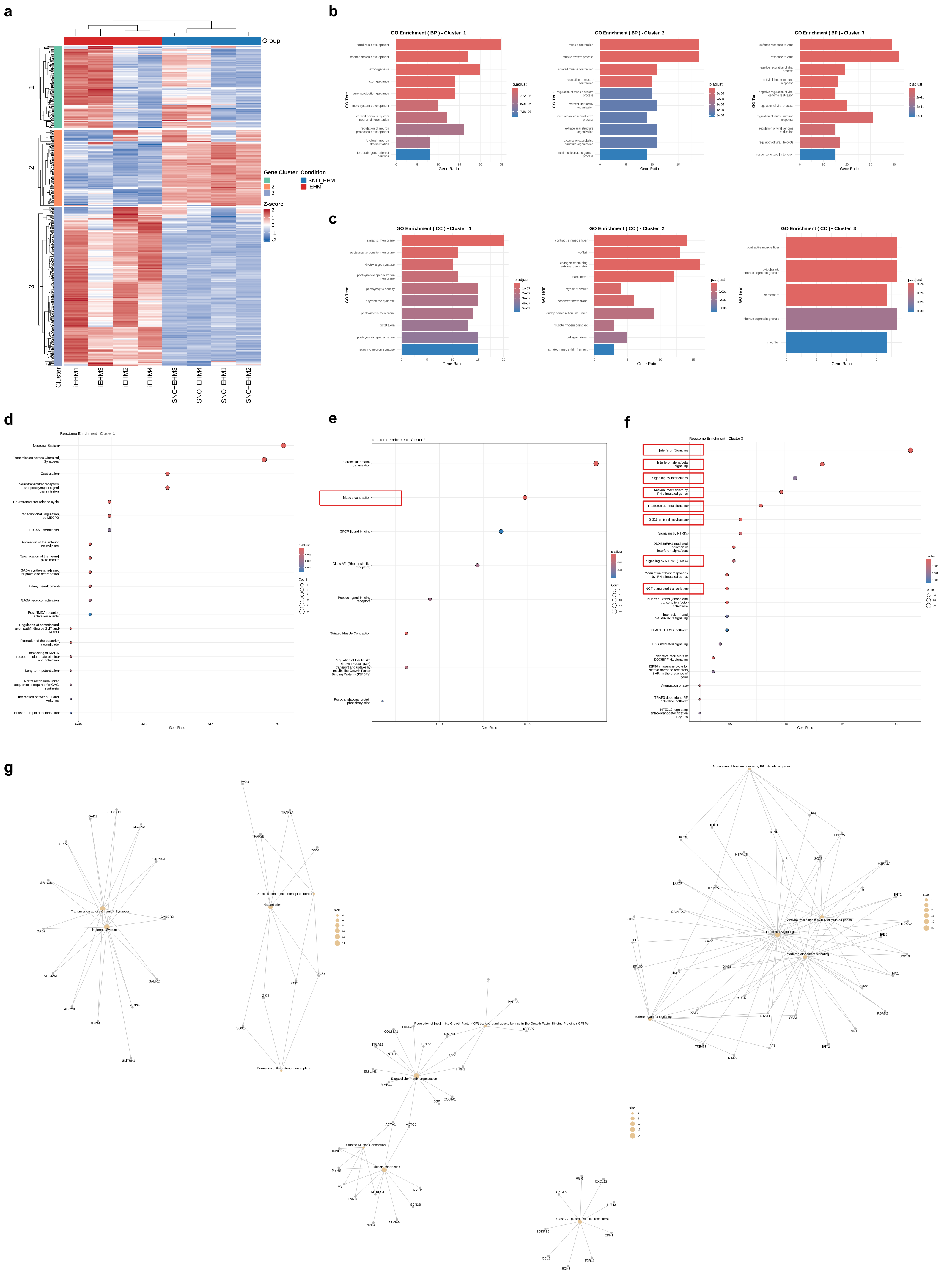

Supplementary Figure 18| Gene ontology from bulk RNA-sequencing of EHM+SNO vs iEHM. **a** Heatmap of variance-stabilized expression values for the top 500 variable genes across iEHM and SNO+EHM samples. Values are Z-score normalized per gene, and hierarchical k-means clustering (k = 3) is applied to visualize global expression differences between conditions. **b–c** Gene Ontology enrichment analysis for the transcriptional clusters. The most significantly enriched Biological Process (BP) terms (**b**) and Cellular Component (CC) terms (**c**) are shown, ranked by gene ratio. Bar length indicates the proportion of genes contributing to each term, and bar color reflects adjusted *P* values. **d–f**, Reactome pathway enrichment for each k-means gene cluster, highlighting distinct pathway signatures associated with the corresponding transcriptional module. **g**, STRING protein–protein interaction (PPI) networks generated from the genes in each expression cluster. Nodes represent proteins and edges denote predicted or experimentally supported functional associations. Network organization reflects cluster membership and node color indicates Reactome pathway enrichment significance.

Supplementary Figure 19

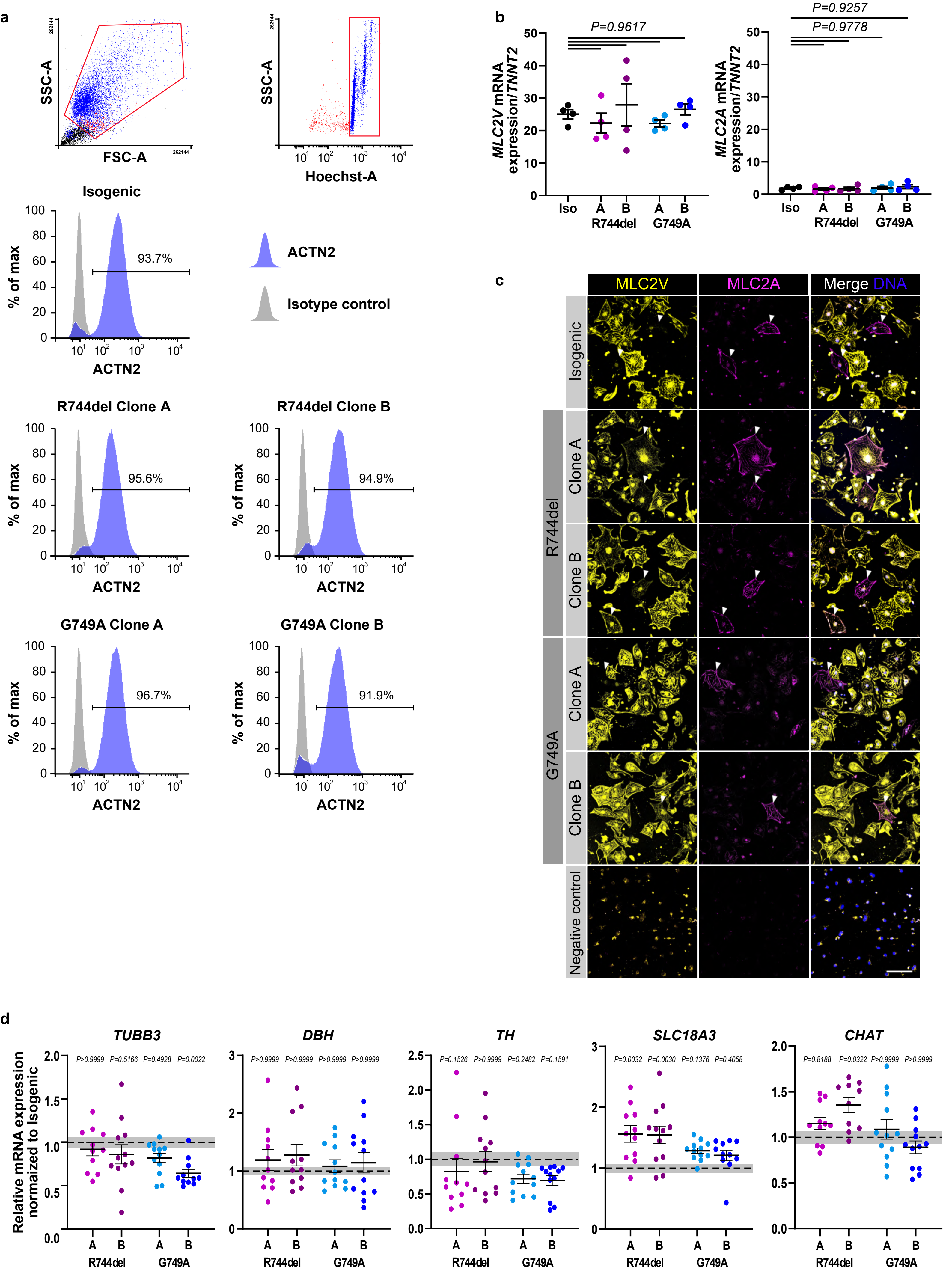

Supplementary Figure 19| Analysis of *KCNH2*-mutant differentiation efficiency to autonomic neurons and CMs. a Flow cytometry analysis of d30 CM stained for CM-marker ACTN2. (Uppermost panels). The gating strategy shows the exclusion of small debris based on size (forward scatter = FSC-A), granularity (side scatter = SSC-A), and DNA content (Hoechst-A). (Below) Histograms of ACTN2-staining fluorescence intensity distribution: ACTN2-stained CM are represented by blue curves and IgG1 isotype-stained negative controls are shown in grey. **b** qPCR expression analysis of myosin light chain's ventricular and atrial isoforms (*MLC2V* and *MLC2A*, respectively) in CM differentiation (d40-d60) from the *KCNH2* mutant lines and isogenic (Iso) control. 1 differentiation, n=4. **c** Immunofluorescent staining of MLC2A (magenta), MLC2V (yellow) and DNA (blue) in d60 CM shows predominant expression of the ventricular isoform. Atrial cells are marked by white arrowheads. Scale bar, 100 µm. **d** mRNA expression of pan-neural marker *TUBB3*, SN markers *DBH* and *TH*, and cholinergic neuron marker *SLC18A3* and *CHAT* of d43 SNO from *KCNH2*-variant lines compared to isogenic control. All values are normalized to isogenic control. Isogenic control expression levels are represented by dashed lines, and grey shading indicates the s.e.m. 3 differentiations, n=3-4 tissues. *TUBB3*: n(Iso, R744del-A, G749A-B)=11, n(R744del-B, G749A-A)=11. *DBH*: n(Iso, G749A-A, G749A-B)=12, n(R744del-A, R744del-B)=11. *TH*: n(Iso, R744del-B, G749A-A, G749A-B)=12, n(R744del-A)=11. *SLC18A3*: n(Iso, R744del-B, G749A-A)=12, n(R744del-A, G749A-B)=11. *CHAT*: n(R744del-B)=10, n(Iso, R744del-A)=11, n(G749A-A, G749A-B)=12. All data is presented as mean ± s.e.m. One-way ANOVA with Holm-Šídák's multiple comparisons test was used for (**b**). One-way ANOVA or Kruskal-Wallis test with Dunn's multiple comparisons post hoc test was used for (**d**).

Supplementary Figure 20

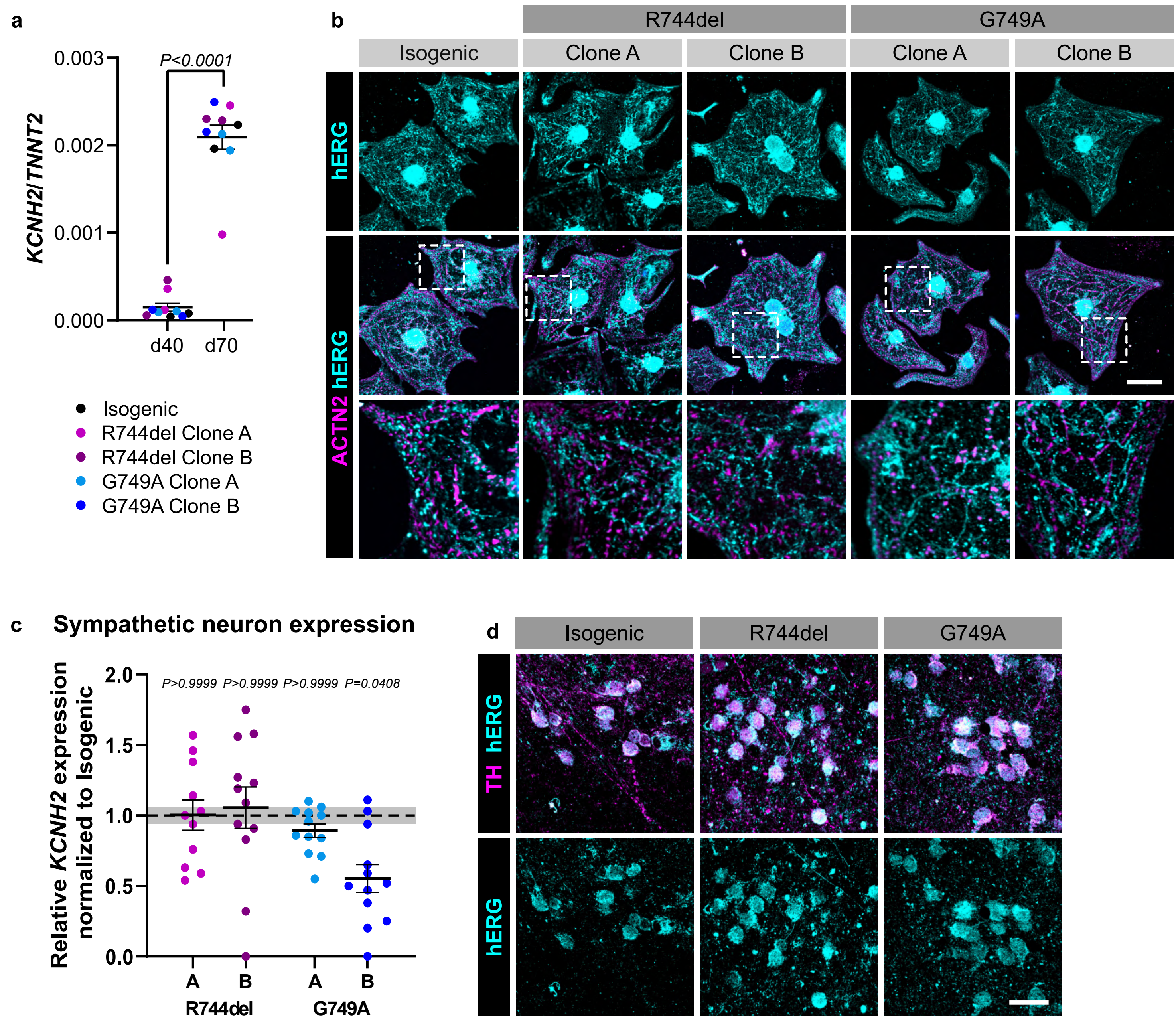

Supplementary Figure 20| *KCNH2* expression levels in CMs and sympathetic neurons. a qPCR analysis of *KCNH2* expression in *KCNH2*-mutant and isogenic control CM compared between d40 and d70 normalized to CM-marker gene *TNNT2*. n=10 / timepoint. **b** Immunofluorescence staining of hERG (cyan) and CM-marker ACTN2 (magenta) in hiPSC-CM at d70. (Lower panels) Detailed view illustrates endoplasmic reticulum and membrane localization of hERG channels. Detailed regions are indicated by white dashed rectangles. Scale bar, 30 µm. **c** *KCNH2* transcript analysis in d43 SNO. Values were normalized to *KCNH2* expression in isogenic SNO. The dashed line and grey shaded area indicate isogenic mean expression and s.e.m., respectively. 3 differentiations, n=3-4. n(Iso, R744del-A)=11, n(R744del-B, G749A-A, G749A-B)=12. **d** hERG immunofluorescent staining (cyan) of SN marked by TH (magenta). Scale bar, 30 µm. All data is presented as mean ± s.e.m. Unpaired two-tailed Mann-Whitney test was used for (**a**) and Kruskal-Wallis test with Dunn's multiple comparisons post hoc test was used for (**c**).

Supplementary Figure 21

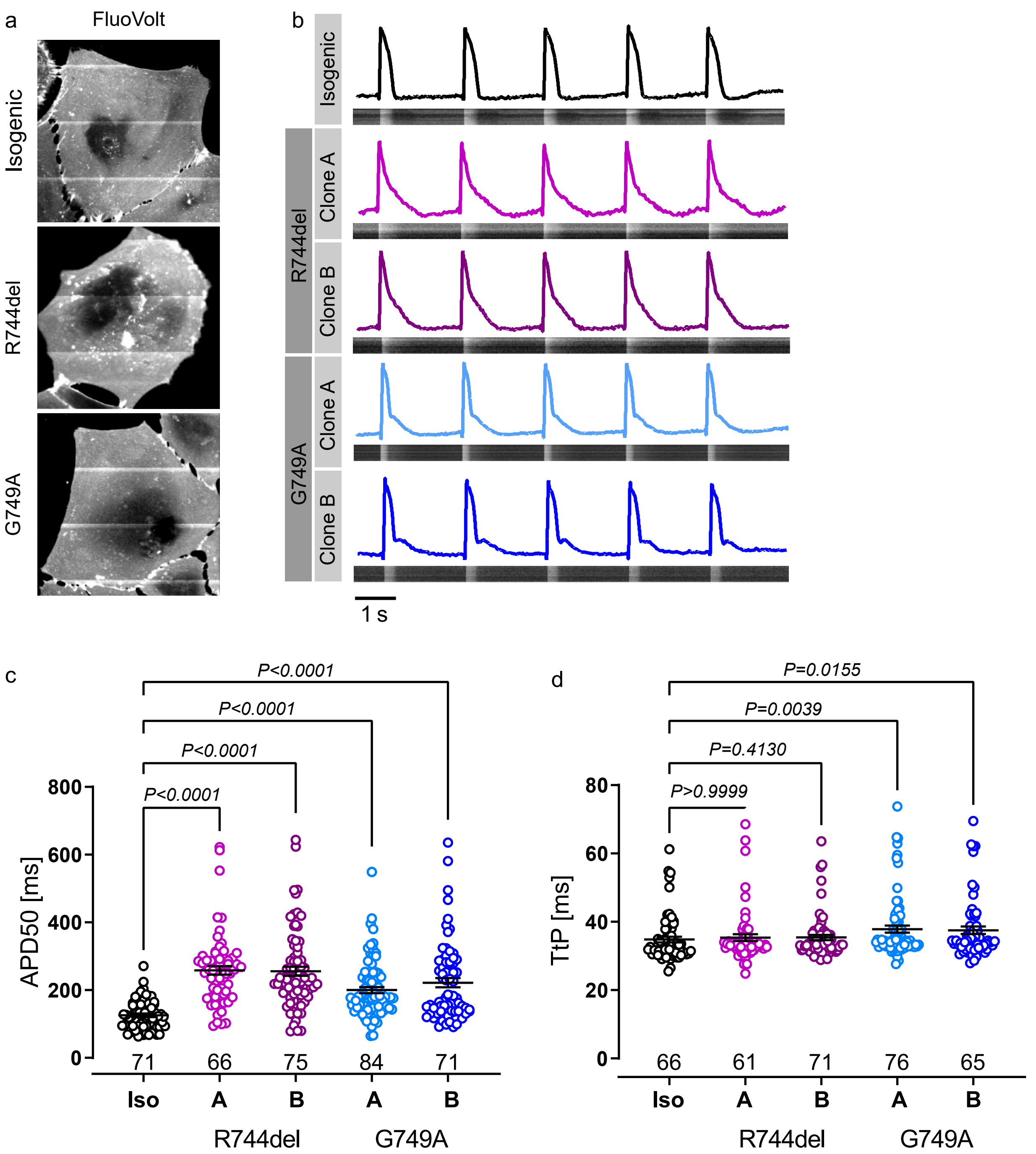

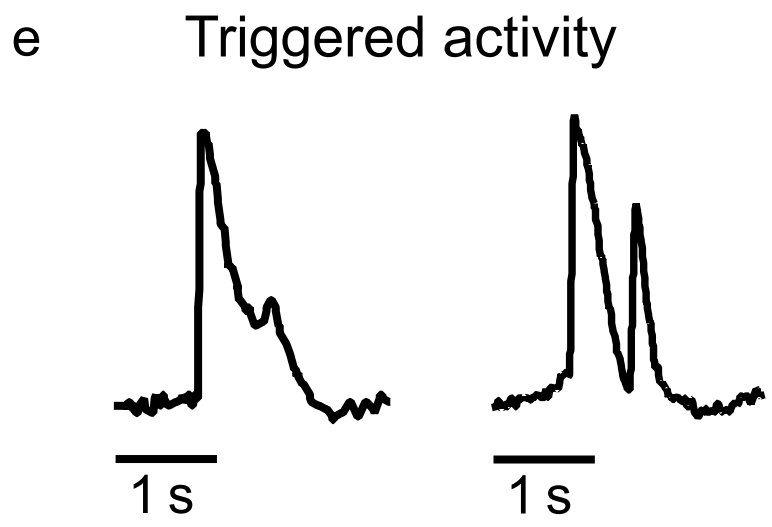

f

	Iso	R744del		G749A	
		Clone A	Clone B	Clone A	Clone B
EAD-like	3	2	10	8	2
Total cells	70	70	45	73	62

Supplementary Figure 21| Optical voltage imaging of *KCNH2* mutant vs isogenic CMs. a Example overview images of CM stained with voltage-sensitive FluoVolt™ dye. Scale bar, 50 µm. **b** Representative traces of optical voltage imaging of ventricular CM from *KCNH2*-mutant lines and isogenic control at d60; 0.5 Hz electrical pacing rate. **c** Action potential durations at 50% repolarization (APD50) from voltage imaging analysis. 3 independent differentiations, n numbers are given in the graph. **d** Rise time (Time to peak = TtP) of *KCNH2*-mutant and isogenic CM calculated from FluoVolt™ voltage traces. 3 independent differentiations, n numbers are given in the graph. **e** Example traces from voltage imaging showing EAD-like events in p.R744del-mutant CM. **f** Quantification of EAD-like events for all lines. All data is presented as mean ± s.e.m. Kruskal-Wallis test with Dunn's multiple comparisons post hoc test was performed for (**c**, **d**).

Supplementary Figure 22

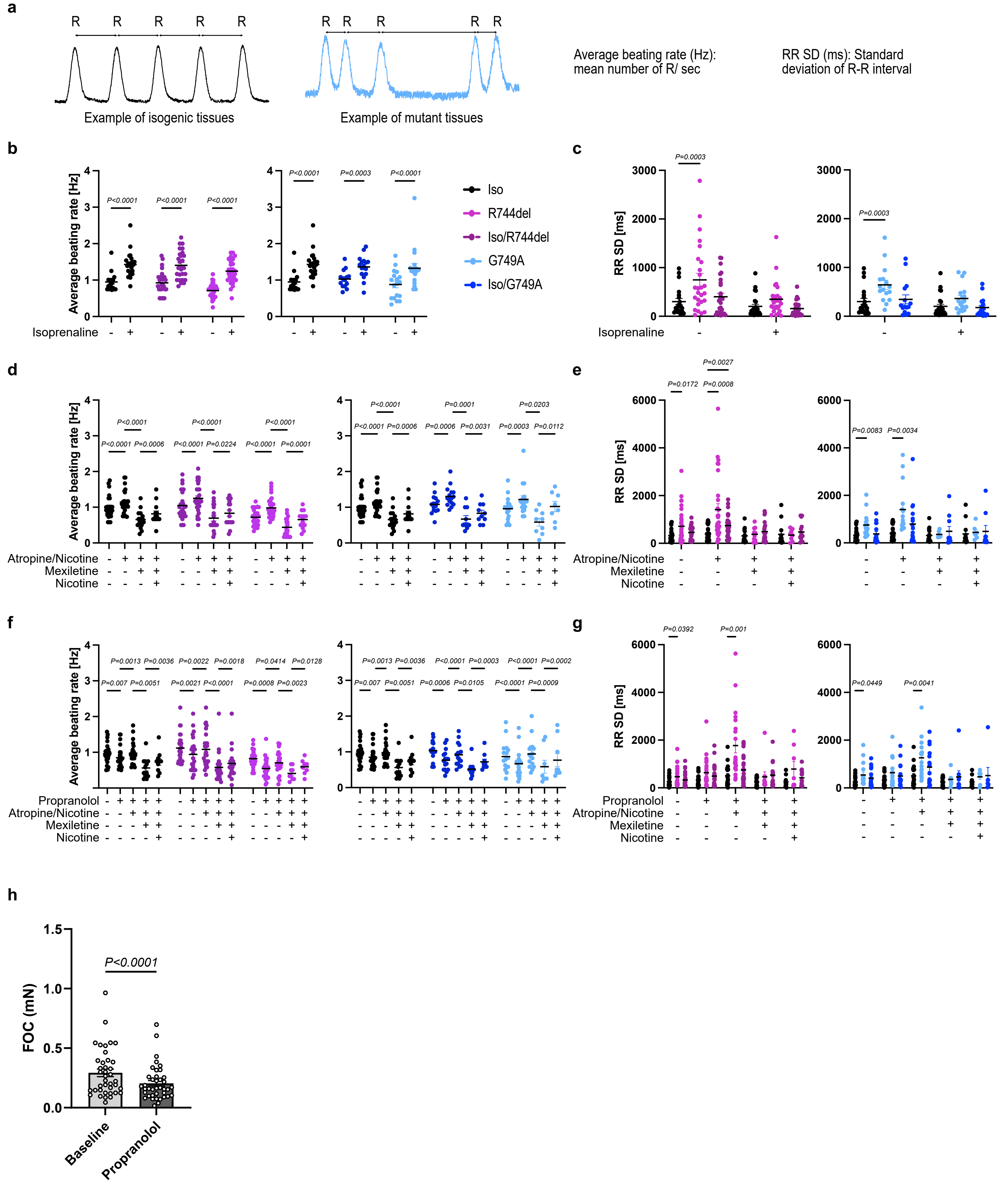

Supplementary Figure 22| Pharmacological modulation of rhythm variability and beating rate in iEHM carrying SUDEP-associated *KCNH2* mutations. a Representative contractility recordings from isogenic and hERG mutant (R744del, G749A) iEHM showing rhythmic force oscillations. Each peak corresponds to one contraction (R wave equivalent). The interval between consecutive contractions was used to calculate the R–R interval and its standard deviation (RR SD, in ms) as a measure of rhythm variability. The frequency of contractions was used to determine the average beating rate (Hz). **b, c** Quantifications of pharmacological responses showing the effects of β-adrenergic stimulation (isoprenaline) on beating rate and RR SD across isogenic and mutant iEHM. 3 independent differentiations, n(ISO)= 24, n(p.R744del)= 28, n(ISO/R744del)= 28, n(p.G749A)= 19, n(ISO/G749A)= 17. **d, e** Quantifications of pharmacological responses showing the effects of SN stimulation (atropine/nicotine), in the presence and absence of sodium channel inhibitor (mexiletine) on RR SD and beating rate across isogenic and mutant iEHM. 3 independent differentiations (Baseline and atropine/nicotine treatments: 3 differentiations, mexiletine and nicotine treatments: 2 differentiations). n(ISO)= 27, n(p.R744del)= 31, n(ISO/R744del)= 28, n(p.G749A)= 17, n(ISO/G749A)= 17. **f, g** Quantifications of pharmacological responses showing the effects of SN stimulation (atropine/nicotine), in the presence and absence of sodium channel inhibitor (mexiletine) and β-adrenoreceptor blocker (propranolol) on beating rate and RR SD across isogenic and mutant iEHM. 3 independent differentiations (baseline, propranolol and atropine/nicotine treatments: 3 differentiations, mexiletine and nicotine treatments: 2 differentiations). n(ISO)=26, n(p.R744del)= 26, n(ISO/R744del)= 27, n(p.G749A)= 18, n(ISO/G749A)= 17. **h** Force of contraction data showing decrease of contractile performance of iEHM treated with propranolol. 3 independent differentiations, n= 39. The data presented in c, e, g are single data points of each tissue corresponding to the graphs in Figure 5 d, f, h. Data is presented as mean ± s.e.m. Two-way ANOVA with Tukey's multiple comparisons post hoc test was performed for (**b**-**g**). Paired two-tailed Wilcoxon test was performed for (**h**).

Supplementary Table 1| Summarizing table comparing protocols and functional characterization of 2D and 3D SN culture systems.

Study	Starting cell population	Dimension	Differentiation factors	Cell contents	Functional analysis	Catecholamine content	Co-culture with hIPSC Cardiomyocytes	Functional connectivity
Oh et al 2016 [2]	hESC (2 lines)<br>hiPSC (1 line)	2D	AA<br>BDNF<br>BMP4<br>CHIR99021<br>DAPT<br>db-cAMP<br>FGF2<br>GDNF<br>LDN193189<br>NGF<br>PD173074<br>PMP<br>SB431542<br>Shh C25II	SN*<br>Enteric neurons<br><br>NO Sensory neurons<br>NO Motor neurons	Patch clamp (d18-d29)<br>• Spontaneous firing<br>• Response to Nicotine stimulation<br>• Response to Optogenetic stimulation	ELISA: Secretion (d18-d29)<br>• DA: 3.27 pmol/ml<br>• NA: 0.27 pmol/ml	-	-
Frith et al 2018 [50]	hESC (3 lines)<br>hiPSC (3 lines)	2D	AA<br>BDNF<br>BMP4<br>CHIR99021<br>DMH1<br>FGF2<br>GDNF<br>NGF<br>PMP<br>SB431542<br>Shh C25II	SN**	Patch clamp (d19-d22)<br>• Spontaneous firing	ELISA: Secretion (d19-d22)<br>• DA: 2.94 pmol/ml<br>• NA: 0.18 pmol/ml	-	-
Kirino et al 2018 [3]	hESC (2 lines)<br>hiPSC (2 lines)	2D	BDNF<br>BMP4<br>CHIR99021<br>EGF<br>FGF2<br>GDNF<br>Heparin<br>NGF<br>RA<br>SB431542	SN**	-	ELISA: Secretion (d40)<br>• NA: 0.24-0.44 pmol/ml	-	-
Takayama et al 2020 [24]	hESC (1 line)<br>hiPSC (2 lines)	2D	AA<br>BDNF<br>BMP4<br>CHIR99021<br>DM<br>FGF2<br>Forskolin<br>GDNF<br>IWR1<br>NGF<br>NT3<br>SANT1<br>SB431542	SN<br>PNS<br>Enteric neurons<br>Non-neuronal (Stromal cells, others)<br>GABAergic neurons	MEA<br>• Response to Optogenetic stimulation<br><br>Calcium imaging (d45-d49)<br>• Rare spontaneous firing<br>• Response to Electrical stimulation<br>• Response to Nicotine stimulation	-	2D	+
Winbo et al 2020 [53]	hiPSC (1 line)	2D	AA<br>BDNF<br>BMP4<br>CHIR99021<br>DAPT<br>db-cAMP<br>GDNF<br>LDN193189<br>NGF<br>PD173074<br>PMP<br>SB431542<br>Shh C25II	SN**	Patch clamp (d28-d76)<br>• Spontaneous firing<br>• Response to Tetrodotoxin	ELISA: Secretion (d75)<br>• NA: 0-0.37 pmol/ml	2D	+
Wu & Zeltner 2020 [51]	hiPSC (2 lines)	2D	AA<br>BDNF<br>BMP4<br>CHIR99021<br>db-cAMP<br>FGF2<br>GDNF<br>NGF<br>RA<br>SB431542	SN<br>PNS<br>Sensory neurons<br>Enteric neurons<br>Motor neurons<br>Myofibroblasts	MEA (d20-d30)<br>• Spontaneous firing<br>• Response to Nicotine stimulation<br>• Response to Propranolol	-	-	-
Bernardin et al 2022 [52]	Rat PC12<br>hiPSC (1 line)	2D	Rat PC12:<br>NGF<br><br>hIPSC:<br>Winbo et al 2020	SN**<br>PNS	-	LCMS: Secretion (d14)<br>• L-DOPA: 0.004 pmol/ml<br>• DA: 0.215 pmol/ml NA: 0.004 pmol/ml<br><br>Fluorometric kit: Secretion (d14)<br>• ACh: 24.39 pmol/ml	2D	-
Haekli et al 2022 [22]	hiPSC (1 line)	2D	AA<br>BDNF<br>db-cAMP<br>FGF2<br>GDNF<br>LDN193189<br>RA<br>SB431542	SN**<br>PNS	-	-	2D	+
Olmsted & Paluh 2022 [57]	hIPSC (1 line)	3D	AA<br>CHIR99021<br>FGF2<br>HGF<br>IGF1<br>VEGF	Peripheral progenitors<br>Anterior foregut<br>Motor neurons<br>Sensory neurons<br>Cardiac cells	-	-	3D	-
Wu et al 2024 [54]	hiPSC (1 line)	2D	AA<br>BDNF<br>BMP4<br>CHIR99021<br>db-cAMP<br>FGF2<br>GDNF<br>NGF<br>SB431542<br>Amphi	SN**	MEA (d20/d30+)<br>• Spontaneous firing<br><br>Calcium imaging (d30+)	-	-	-
Liu, Zhu, Lu et al 2026 [26]	hiPSC (1 line)<br>hESC (2 lines)	3D	AA<br>BMP4<br>CHIR99021<br>DAPT<br>FGF2<br>GDNF<br>NGF<br>RA<br>SAG<br>SB431542	SN	MEA (d70-d80, d130-d150)<br>• Spontaneous firing<br>• Response to Acetylcholine & Nicotine	-	3D	+
Branco et al 2026 [25]	hiPSC (1 line)	3D	AA<br>BDNF<br>CHIR99021<br>DAPT<br>GDNF<br>LDN193189<br>NGF<br>SAG<br>SB431542	SN**	MEA (d50-d62)<br>• Spontaneous firing<br>• Response to Nicotine	ELISA: Secretion (3 days culture)<br>• DA: 1.76 pmol/ml<br>• NA: 0.33 pmol/ml	3D	+
Schneider & Setya et al	hiPSC (3 lines)	3D	BMP4<br>CHIR99021<br>DAPT<br>FGF2<br>LDN193189<br>RA<br>SB431542<br>TGFβ1	SN<br>PSN<br>Sensory neurons<br>Glutamatergic neurons<br>NO Motor neurons	MEA (d28-d56)<br>• Spontaneous firing<br>• Response to Nicotine stimulation<br>• Response to Optogenetic stimulation	LCMS: Cell lysate (d41)<br>• DA: 6.18 pmol/mg<br>• NA: 60.4 pmol/mg<br>• ACh: 19 pmol/mg	3D	+

AA: Ascorbic acid, ACh: Acetylcholine, Amphi: Amphidicolin, BDNF: Brain-derived neurotrophic factor, BMP4: Bone morphogenetic protein 4, CHIR99021: GSK3 inhibitor/Wnt activator, DA: Dopamine, DAPT: γ-secretase inhibitor, db-cAMP: Dibutyryl-cAMP, DM: Dorsomorphin, DMH1: Dorsomorphin homolog 1, EGF: Epidermal growth factor, FGF2: Fibroblast growth factor 2, GDNF:

Glial cell-derived neurotrophic factor, hESC: Human embryonic stem cell, hiPSC: Human induced pluripotent stem cell, HGF: Hepatocyte growth factor, IGF1: Insulin growth factor 1, IWR1: Wnt pathway inhibitor, LDN193189: BMP pathway inhibitor, NA: Noradrenaline, NGF: Nerve growth factor, NT3: Neurotrophin-3, PD173074: FGFR1 inhibitor, PMP: Purmorphamine, RA: Retinoic acid, SAG: Smoothened agonist, SANT1: Sonic hedgehog pathway inhibitor, SB431542: TGFβ pathway inhibitor, Shh C25II: Sonic hedgehog pathway agonist, TGFβ1: Transforming growth factor beta-1, VEGF: Vascular endothelial growth factor

*No PNS marker tested / **No other neuronal marker tested

Supplementary Table 2| List of primary and secondary antibodies

Antigen	Modification	Brand	Cat. No.	Host	Dilution	Type
ACTN2	None	Sigma	A7811	Mouse	1:1000	Primary
BRN3A	None	Merck Millipore	MAB1585	Mouse	1:500	Primary
CTNT	None	Abcam	ab45932	Rabbit	1:250	Primary
CD45	None	Abcam	Ab8216	Mouse	1:100	Primary
CD68	None	Synaptic Systems	HS-460 008	Rabbit	1:100	Primary
DBH	None	Sigma	HPA070789	Rabbit	1:100	Primary
HCN4	None	Abcam	ab69054	Rabbit	1:200	Primary
Neurexin 1	None	Sigma	HPA071400	Rabbit	1:200	Primary
NF (H)	None	Synaptic Systems	171 106	Chicken	1:100	Primary
NF (H)	None	Biolegend	822601	Chicken	1:1000	Primary
PAX6	None	Biolegend	901301	Mouse	1:500	Primary
PDGFRb	None	Cell Signalling	28E1	Rabbit	1:100	Primary
PECAM1	None	DAKO	M0823	Mouse	1:100	Primary
Peripherin	None	NOVUS	NBP1-05423	Chicken	1:1000	Primary
Phalloidin	Alexa Fluor 488	Invitrogen	A12379	-	1:200	Coupled
PHOX2B	None	Santa Cruz	sc-376997	Mouse	1:250	Primary
Synapsin 1	None	Synaptic systems	106 011	Mouse	1:200	Primary
TH	None	Synaptic Systems	213 004	Guinea pig	1:500	Primary
VAChT	None	Synaptic Systems	139 103	Rabbit	1:500	Primary
VGLUT1	Abberior STAR 635P	NanoTag	N1602-Abb635P	Rabbit	1:250	Coupled
Mouse	Alexa Fluor 633	Invitrogen	A21052	Goat	1:400	Secondary
Chicken	Alexa Fluor 488	Invitrogen	A11039	Goat	1:400	Secondary
Rabbit	Alexa Fluor 546	Invitrogen	A11035	Goat	1:400	Secondary
Guinea Pig	Alexa Fluor 546	Invitrogen	A11074	Goat	1:400	Secondary

Supplementary Table 3| Detection parameters for a second compound-specific mass transition used as qualifier. Q1=first quadrupole, Q3=third quadrupole, DP=declustering potential, CE=collision energy, CXP=collision cell exit potential.

**Compound**	**Mass Q1 [Da]**	**Mass Q3 [Da]**	**DP [V]**	**CE [V]**	**CXP [V]**
NA	170.2	152.0<br>(107.0)	36	10<br>-28	10<br>-20
Dopamine	154.1	137.2<br>(91.0)	36	15<br>-33	10<br>-8
Acetylcholine	147.0	87.0<br>(88.0)	36	19<br>-20	16<br>-16
NA-d6	176.1	158.0<br>(111.0)	31	11<br>-29	10<br>-6
Buformin	157.91	60.9<br>(47.0)	36	35<br>-66	10<br>-8
Choline-d9	113.108	69.1<br>(66.1)	66	27<br>-44	12<br>-12

Supplementary Table 4| List of primer for quantitative real-time PCR

Gene	Forward primer	Reverse primer	Amplicon length (bp)
ASCL1	CGGTCTCATCCTACTCGTCG	GTTGTGCGATCACCCTGCTT	150
CHAT	GGATCGCTGGACATGATTG	CCACACCGCCAGGTCGC	103
DBH	TGGGTGCCAAGGCATTTTAC	ATGCCTGAGGAGTCGTTTCG	136
GAD1	AGGCAATCCTCCAAGAACC	TGAAAGTCCAGCACCTTGG	218
GAPDH	CCTCAAGATCATCAGCAATGCC	ATGTTCTGGAGAGCCCCGC	189
GATA2	GCAGAACCGACCACTCATCA	AGTGGCCTGTTAACATTGTGC	177
GBX2	GTTCCCGCCGTCGCTGATGAT	GCCGGTGTAGACGAAATGGCCG	118
GLAST	CCCCTTACAAAATCAGAAAAGTTGT	CCCATCTTGGGCTCTTCTCC	151
MNX1	TGCCTAAGATGCCGACTTC	AATCTTCACCTGGGTCTCGG	194
OLIG2	GCTGCGTCTCAAGATC	AGTCGCTTCATCTCCT	192
PAX6	CCCCACATATGCAGACACAC	TCACTTCCGGGAACTTGAAC	112
PHOX2B	CGCCGCAGTTCCTTACAAAC	TGTCGGGGTAGTGAGTCTCC	140
PLP1	GGTTTCCCTGCTCACCTTCA	ATCAGAACTTGGTGCCTCGG	83
PRPH	TAAATATAAAGACGACTGTGCCTGA	GCAGAAGACTTGTCCAGCTCA	151
PRRX1	CGCAGGAATGAGAGAGCCAT	GAGGGACGAGGATCTGTACG	165
SLC17A6	GTAGACTGGCAACCACCTCC	CCATTCCAAAGCTTCCGTAGAC	129
SLC18A2	TTCCGACTGTCCCAGTGAAG	TGCTGGAGAAGGCAAACATAAT	200
SLC6A2	CAGGTTCAGCAACGACATCCAG	GTCGTAGGTGAGTGGCTTGAAG	142
SOX10	TCTGGAGGCTGCTGAACGAA	GTAGTGGGCCTGGATGGC	200
TH	CGGGGCTTCTCGGACCAGGTGTA	CTCCTCGGCGGTGTACTCCACA	111
TUBB3	CAGCGTCTACTACAACGAGG	AGGCCTGAAGAGATGTCCAA	121
TWIST1	GCCGGAGACCTAGATGTCATT	TTTTAAAAGTGCGCCCCACG	165
SLC18A3	CTGCTAGTGAACCCCTTGAGC	CAGGACTGTAGAGGCGAACAT	99
