# Supplementary material for: Innervated human cardiac muscle model reveals sympathetic drivers of KCNH2-associated arrhythmias
Source: Nat Commun. 2026 Sep 2;17:9404. doi: 10.1038/s41467-026-76956-9 (PMC13538559; doi:10.1038/s41467-026-76956-9)
Supplement: Supplementary file 4 [file 41467_2026_76956_MOESM2_ESM.pdf]

Title: Supplementary Movie 1.

Description: Spontaneous beating of iEHM 1 week after fusion

Title: Supplementary Movie 2.

Description: Representative immunofluorescence picture depicting neurofilament positive axons (green) interlacing alpha-actinin positive CMs (red) in iEHM

Title: Supplementary Movie 3.

Description: Representative immunofluorescence picture 1 depicting synapsin positive pre-synaptic terminals (red) of noradrenergic neurons (TH, blue) in between CMs (cTNT, green) in 8 week-old iEHM.

Title: Supplementary Movie4.

Description: Representative immunofluorescence picture 2 depicting synapsin positive pre-synaptic terminals (red) of noradrenergic neurons (TH, blue) in between CMs (cTNT, green)) in 8 week-old iEHM.

Title: Supplementary Movie5.

Description: Representative immunofluorescence picture 3 depicting synapsin positive pre-synaptic terminals (red) of noradrenergic neurons (TH, blue) in between CMs (cTNT, green)) in 8 week-old iEHM.

Title: Supplementary Movie6.

Description: Representative immunofluorescence picture 4 depicting synapsin positive pre-synaptic terminals (red) of noradrenergic neurons (TH, blue) in between CMs (cTNT, green)) in 8 week-old iEHM.

Title: Supplementary Movie7.

Description: Representative immunofluorescence picture 5 depicting synapsin positive pre-synaptic terminals (red) of noradrenergic neurons (TH, blue) in between CMs (cTNT, green) in 8 week-old iEHM.

Title: Supplementary Movie8.

Description: Representative immunofluorescence picture 6 depicting synapsin positive pre-synaptic terminals (red) of noradrenergic neurons (TH, blue) in between CMs (cTNT, green) in 8 week-old iEHM.

Title: Supplementary Movie9.

Description: Representative immunofluorescence picture 1 depicting PECAM1 positive vascular network (red) surrounded by PDGFRb positive pericytes (cyan) in close proximity to neurons (NF, green) in 8 week-old iEHM.

Title: Supplementary Movie10.

Description: Representative immunofluorescence picture 2 depicting PECAM1 positive vascular network (red) surrounded by PDGFRb positive pericytes (cyan) in close proximity to neurons (NF, green) in 8 week-old iEHM.

Title: Supplementary Movie11.

Description: Representative immunofluorescence picture 3 depicting PECAM1 positive vascular network (red) surrounded by PDGFRb positive pericytes (cyan) in close proximity to neurons (NF, green) in 8 week-old iEHM.

Title: Supplementary Movie12.

Description: Chronotropic response of EHM and optogenetic iEHM evoked by light stimulation.
